# Supplementary material for: A taxonomy for detecting and preventing temporal data leakage in machine learning-based build prediction: A dual-platform empirical validation
Source: PLoS One. 2026 May 27;21(5):e0340167. doi: 10.1371/journal.pone.0340167 (PMC13215522; doi:10.1371/journal.pone.0340167)
Supplement: Attachment — Submitted filename: Reviewers Comments.pdf [file pone.0340167.s001.pdf]

**Title:** A taxonomy for detecting and preventing temporal data leakage in machine learning based build prediction: A dual-platform empirical validation

**Manuscript ID:** PONE-D-25-66966

**Article type:** Research article

PLOS ONE

**# Reviewer's Comments:**

1. Revise the abstract so that it is presented as a single, coherent paragraph.
2. The introduction section is overly lengthy and should be revised to more clearly highlight the contributions of previous studies in addressing the identified problem. Clearly articulate the existing gap or prevailing challenge, then introduce the proposed methodology with a logical justification explaining why it is suitable for addressing the problem.
3. Explicit research questions are not strictly necessary in a research article. You may remove this section and instead clearly state the main contributions of the paper.
4. Revise the related work section to be more concise, focusing primarily on recent and closely related studies published in reputable journals.
5. The presentation of several sections resembles a tutorial rather than a research article. Consider replacing lengthy textual explanations with figures, flowcharts, and tables where appropriate to improve clarity and conciseness.
6. There is an overreliance on accuracy as the primary performance metric despite the presence of class imbalance, particularly in the GHALogs dataset. This limits the operational interpretability of the results, as practical CI/CD deployment decisions often depend more on precision–recall trade-offs than on raw accuracy. Incorporate cost-sensitive analysis, precision–recall curves, and scenario-based error cost evaluations.
7. Although strong claims of generalizability are made, the evaluation is limited to only two CI/CD platforms. No validation is provided for self-hosted CI systems such as Jenkins, enterprise CI platforms such as GitLab CI and Azure DevOps, or monorepo-heavy environments.
8. The conclusion section currently reads more like a summary. Rewrite it to concisely restate the proposed approach and interpret the results, highlighting the significance of the findings, the impact of the study, and potential directions for future research.
