# Supplementary material for: A taxonomy for detecting and preventing temporal data leakage in machine learning-based build prediction: A dual-platform empirical validation
Source: PLoS One. 2026 May 27;21(5):e0340167. doi: 10.1371/journal.pone.0340167 (PMC13215522; doi:10.1371/journal.pone.0340167)

PLOS One

A taxonomy for detecting and preventing temporal data leakage in machine learning-based build prediction: A dual-platform empirical validation

--Manuscript Draft--

**Manuscript Number:**	PONE-D-25-66966
**Article Type:**	Research Article
**Full Title:**	A taxonomy for detecting and preventing temporal data leakage in machine learning-based build prediction: A dual-platform empirical validation
**Short Title:**	Temporal Data Leakage in Build Prediction
**Corresponding Author:**	Lalit Narayan Mishra, Masters in Computer Science<br>Lowe's US Inc<br>Mooresville, NC UNITED STATES OF AMERICA
**Keywords:**	
**Abstract:**	Modern software development relies on automated build systems that compile and test code whenever developers make changes. Predicting whether these builds will succeed or fail before execution could save computational resources and developer time. However, many machine learning models for build prediction suffer from temporal data leakage: a methodological flaw where the model inadvertently uses information that would only be available after the build completes, producing artificially inflated accuracy that fails in real-world deployment.<br>We develop a three-type taxonomy to systematically identify and prevent such leakage: (1) Direct Outcome Encoding (using the build result itself as a feature), (2) Execution-Dependent Metrics (information generated during build execution), and (3) Future Information Leakage (using data from chronologically later builds). Applying this taxonomy reveals that prior studies reporting 95-99% accuracy likely used contaminated features, while realistic accuracy is substantially lower.<br>We validate our methodology on 175,706 builds from two platforms spanning 11 years: TravisTorrent (100,000 builds, 2013-2017) and GHALogs (75,706 workflows, 2023). Removing leaky features reduces accuracy by 15.07 percentage points on TravisTorrent (97.8% to 82.73%) but only 0.48 points on GHALogs (83.77% to 83.30%). This platform-dependent difference reveals that modern GitHub Actions' tight integration with repositories enables accurate prediction from static project metadata alone, while older Travis CI systems benefited from time-varying popularity metrics.<br>Using only legitimately available pre-build features, Random Forest classifiers achieve 82.73% (TravisTorrent) and 83.30% (GHALogs) accuracy, which is sufficient for practical deployment. Surprisingly, project maturity and build history prove more predictive than code complexity metrics, suggesting organizational factors outweigh code quality. The models generalize across programming languages (Java, Ruby, Python, JavaScript) with minimal performance variation. We provide open-source tools for detecting temporal leakage in any software prediction task.
**Order of Authors:**	Lalit Narayan Mishra, Masters in Computer Science
	Amit Rangari
	Sandesh Nagrare
	Saroj Kumar Nayak
**Additional Information:**	
**Question**	**Response**
**Financial Disclosure**<br><br>Enter a financial disclosure statement that describes the sources of funding for the work included in this submission. Review the submission guidelines for detailed	The author(s) received no specific funding for this work.

Powered by Editorial Manager® and ProduXion Manager® from Aries Systems Corporation

requirements. View published research articles from *PLOS ONE* for specific examples.<br><br>This statement is required for submission and **will appear in the published article** if the submission is accepted. Please make sure it is accurate.<br><br>**Funded studies**<br>Enter a statement with the following details:<br>• Initials of the authors who received each award<br>• Grant numbers awarded to each author<br>• The full name of each funder<br>• URL of each funder website<br>• Did the sponsors or funders play any role in the study design, data collection and analysis, decision to publish, or preparation of the manuscript?<br><br>Did you receive funding for this work?	
**Competing Interests**<br><br>Use the instructions below to enter a competing interest statement for this submission. On behalf of all authors, disclose any competing interests that could be perceived to bias this work—acknowledging all financial support and any other relevant financial or non-financial competing interests.<br><br>This statement is required for submission and **will appear in the published article** if the submission is accepted. Please make sure it is accurate and that any funding sources listed in your Funding Information later in the submission form are also declared in your Financial Disclosure statement.<br><br>View published research articles from *PLOS ONE* for specific examples.	The authors have declared that no competing interests exist.

Powered by Editorial Manager® and ProduXion Manager® from Aries Systems Corporation

**NO authors have competing interests**<br><br>Enter: *The authors have declared that no competing interests exist.*<br><br>**Authors with competing interests**<br><br>Enter competing interest details beginning with this statement:<br><br>*I have read the journal's policy and the authors of this manuscript have the following competing interests: [insert competing interests here]*<br><br>* typeset	
**Ethics Statement**<br><br>Enter an ethics statement for this submission. This statement is required if the study involved:<br><br>• Human participants<br>• Human specimens or tissue<br>• Vertebrate animals or cephalopods<br>• Vertebrate embryos or tissues<br>• Field research<br><br>Write "N/A" if the submission does not require an ethics statement.<br><br>General guidance is provided below. Consult the submission guidelines for detailed instructions. **Make sure that all information entered here is included in the Methods section of the manuscript.**	N/A

**Format for specific study types**<br><br>**Human Subject Research (involving human participants and/or tissue)**<br>• Give the name of the institutional review board or ethics committee that approved the study<br>• Include the approval number and/or a statement indicating approval of this research<br>• Indicate the form of consent obtained (written/oral) or the reason that consent was not obtained (e.g. the data were analyzed anonymously)<br><br>**Animal Research (involving vertebrate animals, embryos or tissues)**<br>• Provide the name of the Institutional Animal Care and Use Committee (IACUC) or other relevant ethics board that reviewed the study protocol, and indicate whether they approved this research or granted a formal waiver of ethical approval<br>• Include an approval number if one was obtained<br>• If the study involved *non-human primates*, add *additional details* about animal welfare and steps taken to ameliorate suffering<br>• If anesthesia, euthanasia, or any kind of animal sacrifice is part of the study, include briefly which substances and/or methods were applied<br><br>**Field Research**<br><br>Include the following details if this study involves the collection of plant, animal, or other materials from a natural setting:<br>• Field permit number<br>• Name of the institution or relevant body that granted permission	
**Data Availability**<br><br>Authors are required to make all data underlying the findings described fully available, without restriction, and from the time of publication. PLOS allows rare exceptions to address legal and ethical concerns. See the PLOS Data Policy and FAQ for detailed information.	Yes - all data are fully available without restriction

A Data Availability Statement describing where the data can be found is required at submission. Your answers to this question constitute the Data Availability Statement and **will be published in the article**, if accepted.<br><br>**Important:** Stating 'data available on request from the author' is not sufficient. If your data are only available upon request, select 'No' for the first question and explain your exceptional situation in the text box.<br><br>Do the authors confirm that all data underlying the findings described in their manuscript are fully available without restriction?	
**Describe where the data may be found in full sentences. If you are copying our sample text, replace any instances of XXX with the appropriate details.**<br><br>• If the data are **held or will be held in a public repository**, include URLs, accession numbers or DOIs. If this information will only be available after acceptance, indicate this by ticking the box below. For example: *All XXX files are available from the XXX database (accession number(s) XXX, XXX.).*<br>• If the data are all contained **within the manuscript and/or Supporting Information files**, enter the following: *All relevant data are within the manuscript and its Supporting Information files.*<br>• If neither of these applies but you are able to provide **details of access elsewhere**, with or without limitations, please do so. For example:<br><br>*Data cannot be shared publicly because of [XXX]. Data are available from the XXX Institutional Data Access / Ethics Committee (contact via XXX) for researchers who meet the criteria for access to confidential data.*<br><br>*The data underlying the results presented in the study are available from (include the name of the third party*	https://doi.org/10.5281/zenodo.17745286

and contact information or URL).<br>• This text is appropriate if the data are owned by a third party and authors do not have permission to share the data.<br><br>* typeset	
Additional data availability information:	

Click here to access/download;Manuscript;A_taxonomy_for_detecting_and_pr

A taxonomy for detecting and preventing temporal data leakage in machine learning-based build prediction: A dual-platform empirical validation

Lalit Narayan Mishra[1*], Amit Rangari[2], Sandesh Nagrare[3], Saroj Kumar Nayak[4]

1 Lowe's Companies, Inc., Charlotte, NC, USA
2 JPMorgan Chase & Co, Atlanta, GA, USA
3 Digital Remedy, New York, NY, USA
4 Cognizant Technology Solutions, Charlotte, NC, USA

* Lalit Narayan Mishra (Lnm8910@gmail.com)

Abstract

Modern software development relies on automated build systems that compile and test code whenever developers make changes. Predicting whether these builds will succeed or fail before execution could save computational resources and developer time. However, many machine learning models for build prediction suffer from temporal data leakage: a methodological flaw where the model inadvertently uses information that would only be available after the build completes, producing artificially inflated accuracy that fails in real-world deployment.

We develop a three-type taxonomy to systematically identify and prevent such leakage: (1) Direct Outcome Encoding (using the build result itself as a feature), (2) Execution-Dependent Metrics (information generated during build execution), and (3) Future Information Leakage (using data from chronologically later builds). Applying this taxonomy reveals that prior studies reporting 95-99% accuracy likely used contaminated features, while realistic accuracy is substantially lower.

We validate our methodology on 175,706 builds from two platforms spanning 11 years: TravisTorrent (100,000 builds, 2013-2017) and GHALogs (75,706 workflows, 2023). Removing leaky features reduces accuracy by 15.07 percentage points on TravisTorrent (97.8% to 82.73%) but only 0.48 points on GHALogs (83.77% to 83.30%). This platform-dependent difference reveals that modern GitHub Actions' tight integration with repositories enables accurate prediction from static project metadata alone, while older Travis CI systems benefited from time-varying popularity metrics.

Using only legitimately available pre-build features, Random Forest classifiers achieve 82.73% (TravisTorrent) and 83.30% (GHALogs) accuracy, which is sufficient for practical deployment. Surprisingly, project maturity and build history prove more predictive than code complexity metrics, suggesting organizational factors outweigh code quality. The models generalize across programming languages (Java, Ruby, Python, JavaScript) with minimal performance variation. We provide open-source tools for detecting temporal leakage in any software prediction task.

Introduction

Background and Motivation

In modern software development, every time a programmer modifies code, an automated system called a "build" compiles the changes, runs tests, and checks whether

the software still works correctly, similar to how scientific experiments must be reproducible before publication. These automated builds, managed by Continuous Integration/Continuous Deployment (CI/CD) systems, are the gatekeepers of software quality, executing millions of times daily across technology companies worldwide [1]. When builds fail (occurring in 20-40% of cases [2,3]), they block software releases, waste computational resources equivalent to thousands of CPU-hours, and require 30-60 minutes of expert developer time to diagnose, costing organizations $5-10 per failed build in cloud infrastructure alone [4].

Predicting whether a build will succeed or fail before it executes could revolutionize software development efficiency, much like predicting protein folding saves years of laboratory experiments. Organizations could prioritize critical builds, allocate resources intelligently, and provide instant feedback to developers. However, many machine learning studies claiming 95-99% accuracy in build prediction [5] suffer from a methodological flaw analogous to using tomorrow's stock prices to predict today's market: temporal data leakage.

Temporal data leakage occurs when prediction models inadvertently use information from the future: data that would only exist after the event being predicted has occurred. In build prediction, this means training models with features like test results, execution times, or failure logs that are only available after the build completes. While such models achieve impressive accuracy when analyzing historical data retrospectively, they fail catastrophically in real-world deployment where future information remains unavailable. This is equivalent to a medical diagnostic model using biopsy results to predict whether a biopsy is needed, which is methodologically flawed and practically useless.

Accurate build prediction before execution would enable organizations to prioritize critical builds, allocate additional testing resources to high-risk code changes, schedule potentially failing builds during low-traffic periods, and provide developers with immediate feedback on code quality. However, achieving reliable build prediction requires addressing the fundamental data quality problem of temporal data leakage.

Prior research on CI/CD build prediction has reported remarkably high accuracies of 95-99% [5], yet these predictive systems remain largely undeployed in practice. We demonstrate that these impressive results likely stem from temporal data leakage rather than genuine predictive power. Consider a typical machine learning pipeline for build prediction: researchers extract features from historical builds (compilation records from CI/CD systems), train classification models to distinguish successful builds from failed builds, and report cross-validated accuracy. The widely-used TravisTorrent dataset [6], employed in over 40 published papers, contains 66 features per build extracted from Travis CI logs and GitHub repository metadata. However, critical features such as `tr_status` (build outcome itself), `tr_duration` (execution time known only after completion), and `tr_log_tests_failed` (test results available only after test execution) are computable only after build execution completes. Including these features during model training creates classifiers that achieve perfect retrospective discrimination (identifying already-completed build outcomes) but fail completely when deployed prospectively (predicting outcomes before builds execute).

This represents a specific instance of temporal data leakage: using information from the future to predict the past. In production deployment, a build prediction model receives only pre-build information (code changes submitted by developers, project characteristics, team collaboration patterns, and historical build outcomes) and must predict whether the upcoming build will succeed or fail before any compilation or testing occurs. Training models with post-execution features violates this fundamental temporal constraint, rendering reported accuracies scientifically invalid for practical deployment assessment.

The Temporal Data Leakage Problem

Kaufman et al. [7] established the foundational taxonomy of data leakage in machine learning, identifying three primary categories: (1) target leakage (response variable present in training features), (2) train-test contamination (test data influencing training), and (3) temporal leakage (future information affecting past predictions). Recent work by Kapoor and Narayanan [8] demonstrated that data leakage contributes to a reproducibility crisis across 17 scientific fields, affecting 294 published papers and often eliminating claimed performance advantages when leakage is corrected. While their frameworks addressed general machine learning applications, software engineering datasets exhibit unique temporal leakage risks due to the sequential, time-dependent nature of development activities.

Temporal leakage manifests across multiple software engineering prediction tasks. In defect prediction (identifying code modules likely to contain bugs), models may inadvertently use post-release bug counts or incorporate developer bug-fixing experience computed including future observations [9]. Test selection approaches (choosing which tests to execute for efficient fault detection) risk incorporating test execution outcomes or code coverage results unavailable before test runs execute [10]. Code review automation (predicting review effort or approval likelihood) may include features such as review comment counts or time-to-approval metrics computable only after review completion [11].

Despite growing awareness of this methodological problem, no systematic methodology exists for detecting and preventing temporal leakage across software engineering prediction tasks. Prior work identifies specific instances of leakage in individual studies but lacks generalizable frameworks providing detection rules and prevention guidelines applicable across diverse domains. For CI/CD build prediction specifically, multiple studies report 95-99% accuracies [5] using deep learning architectures, yet detailed feature analysis reveals extensive use of outcome-dependent variables. The DL-CIBuild study [5] achieved over 95% accuracy using Long Short-Term Memory (LSTM) neural networks with features including `tr_status` (the build outcome itself), `tr_duration` (post-execution timing information), and `tr_log_tests_failed` (requiring complete test execution). These features provide perfect discrimination when analyzing historical data retrospectively but offer zero predictive utility when deployed prospectively in production systems.

In contrast, studies restricting analyses to genuinely pre-build features (information available before build execution begins) report substantially lower accuracies of 75-84% [2, 3]. This 15-20 percentage point accuracy gap quantifies the magnitude of temporal leakage impact in published CI/CD prediction literature, representing the difference between methodologically sound empirical research and inflated results unsuitable for practical deployment.

Dual-Dataset Validation Strategy

To address concerns about dataset age, CI/CD platform evolution, and generalizability of findings across different infrastructure implementations, we validate our temporal leakage taxonomy and prediction methodology on two independent datasets spanning 11 years of CI/CD ecosystem evolution:

TravisTorrent (Historical Baseline, 2013-2017): The TravisTorrent dataset [6] combines Travis CI build logs with GitHub repository metadata, providing 2.64 million builds from 1,283 open-source projects using container-based virtualization on the Travis CI platform. Programming languages include Java, Ruby, Python, and JavaScript, representing diverse software development ecosystems. For computational tractability while maintaining statistical power, we analyze a stratified random sample

of 100,000 builds preserving the original distribution of build outcomes, programming languages, and project characteristics.

GHALogs (Modern Validation, 2023): The GHALogs dataset [12] provides 513,000 GitHub Actions workflow runs from 25,000 repositories collected in October 2023. We enriched this dataset using the GitHub REST API to extract commit-level metrics, repository characteristics, and team collaboration patterns. After data cleaning and validation, our final enriched dataset contains 75,706 unique workflow runs from 7,620 repositories (87.4% data completion rate) using GitHub Actions runners with distributed workflow execution architecture.

This dual-platform, 11-year validation demonstrates our methodology's robustness across major CI/CD infrastructure shifts: Travis CI containers (2013-2017) to GitHub Actions runners (2023), centralized CI services to distributed workflow execution, and early-generation tooling to containerization ubiquity. Critically, divergent temporal leakage impact patterns emerge across platforms (TravisTorrent: 15.07 percentage point accuracy inflation versus GHALogs: 0.48 percentage point inflation, difference = 14.59 percentage points), revealing platform-dependent metadata predictiveness. GitHub Actions' tight integration with GitHub repository data enables accurate prediction from static project metadata alone (repository age, star count, contributor patterns), whereas Travis CI benefits substantially from time-dependent popularity metrics (stars, forks, watchers) that change during the prediction period. Despite these platform-specific differences, feature importance structural patterns remain stable: build sequence metrics and project maturity consistently dominate code-level metrics across both platforms, suggesting the methodology's potential applicability to other CI/CD platforms such as GitLab CI, CircleCI, and Jenkins X, though this hypothesis requires empirical validation on those specific platforms.

Research Questions

This research investigates machine learning effectiveness for CI/CD build prediction through five specific questions evaluating both methodological reliability and practical deployment viability:

RQ1: Can Software Development Lifecycle (SDLC) metrics effectively predict build outcomes using only pre-build features?

We examine whether project characteristics measurable before build execution (including project maturity, code complexity, test organization, team collaboration patterns, and historical build outcomes) can successfully forecast build success or failure. This requires establishing rigorous methods for detecting and eliminating temporal data leakage, where outcome-dependent features produce artificially inflated accuracies.

RQ2: Which SDLC phases and metrics contribute most to build prediction accuracy?

We evaluate feature importance to identify which project characteristics generate the most accurate predictions: project-level attributes (maturity, age, popularity), code metrics (complexity, churn, files changed), test indicators (test count, test organization), or team collaboration patterns (contributor count, commit frequency, review activity).

RQ3: How well do prediction models generalize across different programming languages and project types?

We assess model performance across Java, Ruby, Python, and JavaScript projects to determine cross-language generalization capability and identify whether language-specific models are necessary or whether a single multi-language model suffices.

RQ4: What is the impact of data leakage prevention on model performance and practical deployment viability?

We quantify the exact accuracy difference between models trained with complete feature sets (including outcome-dependent variables that produce inflated performance)

and models trained exclusively with pre-build features (representing realistic deployment scenarios). This establishes the magnitude of temporal leakage impact and validates whether leakage-free models achieve sufficient accuracy for practical deployment.

RQ5: Do findings generalize across CI/CD platforms and temporal periods?

We evaluate whether our temporal leakage taxonomy, feature importance rankings, and performance characteristics remain consistent when validated on modern GitHub Actions data (2023) versus historical Travis CI data (2013-2017), spanning 11 years of CI/CD infrastructure evolution and major platform architecture changes.

Research Contributions

This research presents five main contributions advancing CI/CD build prediction methodology through dual-platform empirical validation:

1. **Systematic Three-Type Temporal Leakage Taxonomy with Platform-Dependent Empirical Quantification**: We develop a comprehensive temporal data leakage taxonomy categorizing features as (1) Direct Outcome Encoding (features directly revealing build results, such as `tr_status`), (2) Execution-Dependent Metrics (information available only after build completion, such as `tr_duration` or test execution counts), or (3) Future Information Leakage (using data from subsequent builds to predict earlier builds). Cross-platform validation reveals divergent leakage impact patterns: 15.07 percentage point accuracy inflation on TravisTorrent (Travis CI, 2013-2017) versus remarkably low 0.48 percentage point inflation on GHALogs (GitHub Actions, 2023). This divergence demonstrates that CI/CD platform architecture fundamentally affects metadata predictiveness: GitHub Actions' tight integration with GitHub repository infrastructure enables accurate prediction from static project features alone, whereas Travis CI benefits substantially from time-dependent popularity metrics (stars, forks, watchers) unavailable at prediction time.

2. **Dual-Dataset Validation Spanning 11 Years and 175,706 Builds**: We validate our methodology on 175,706 builds from two independent datasets (TravisTorrent: 100,000 Travis CI builds from 1,283 projects, 2013-2017; GHALogs: 75,706 GitHub Actions workflows from 7,620 repositories, 2023), demonstrating temporal robustness across major CI/CD infrastructure evolution. Leakage-free Random Forest classifiers achieve 82.73% accuracy on TravisTorrent (31 pre-build features) and 83.30% accuracy on GHALogs (29 pre-build features), validating practical deployment viability with minimal cross-platform performance variation (0.57 percentage point difference). These results represent realistic deployment performance, contrasting with methodologically flawed studies reporting 95-99% accuracies using outcome-dependent features.

3. **Build Sequence and Project Maturity as Universal Predictors with Platform-Specific Emphasis**: Feature importance analysis reveals that build sequence position (sequential build number in project history) dominates predictions on modern platforms (run_number: 13.17% importance on GHALogs versus tr_build_number: 6.12% on TravisTorrent), while project maturity (days since project creation) remains consistently strong across platforms (gh_project_maturity_days: 9.49% TravisTorrent, repo_age_days: 8.14% GHALogs). Both substantially outweigh code-level metrics (code complexity: 7.7% TravisTorrent, code_lines: 8.54% GHALogs), challenging conventional code-centric software engineering paradigms. This demonstrates that organizational build

experience and project stability predict build outcomes more reliably than immediate code characteristics such as lines changed or cyclomatic complexity.

4. **Cross-Language Generalization Evidence**: TravisTorrent analysis across four programming languages shows minimal performance variation (Java: 84.2%, Ruby: 82.1%, Python: 81.5%, JavaScript: 80.8%, range: 3.38 percentage points). This validates that project maturity and test structure provide language-agnostic predictive signals, enabling deployment of single multi-language models without requiring per-language model retraining or language-specific feature engineering.

5. **Open-Source Temporal Leakage Detection Toolkit**: We provide automated software tools for temporal feature availability auditing, applicable beyond CI/CD build prediction to defect prediction, test selection, and code review prioritization. Our complete replication package (Zenodo DOI: 10.5281/zenodo.17745286) includes processed datasets (TravisTorrent 100,000 builds, GHALogs 75,706 workflows), complete training and evaluation code, platform-specific feature mapping documentation, and automated leakage detection scripts enabling researchers to validate our findings and extend our methodology to emerging CI/CD platforms.

Related Work Context

Our work builds upon three fundamental research areas: machine learning applications in software engineering, CI/CD analytics and build prediction, and temporal data integrity in predictive modeling.

Machine Learning for Software Engineering

Machine learning has transformed software engineering through applications in automated testing, defect prediction, and development analytics [13, 14]. Systematic literature reviews demonstrate that machine learning and deep learning have advanced software engineering tasks, though defect prediction requires improved methodological rigor [15–18]. Recent comprehensive surveys [19] analyzed 138 studies (2011-2024) on ML-based vulnerability detection, noting significant growth from 2020-2024 and emphasizing the need for rigorous evaluation methodologies. Grillmeyer et al. [20] introduced novel measures for quantifying data leakage in failure prediction tasks, demonstrating that leakage-prone data splitting techniques significantly overestimate model performance, a finding directly applicable to CI/CD build prediction. Deep learning approaches including LSTM (Long Short-Term Memory) neural networks [21, 22] and recurrent architectures have shown promise for software defect prediction and code analysis. Transformer-based architectures including Temporal Fusion Transformer and FastFormer have demonstrated capabilities for time-series forecasting and quality-of-service prediction [23–25]. Graph Neural Networks successfully extract structural patterns from code representations for defect prediction [26]. Transfer learning approaches enable cross-project and few-shot prediction scenarios [27]. However, these methods require extensive training data, longer training times, and careful hyperparameter configuration [28], while offering limited interpretability compared to ensemble methods such as Random Forest.

Ensemble learning methods generate superior prediction results for diverse software engineering tasks [29–35]. Random Forest and Gradient Boosting represent state-of-the-art approaches for software defect prediction [9, 36]. XGBoost (Extreme Gradient Boosting) remains a top choice for software engineering applications due to strong performance on tabular feature data [37, 38]. Feature selection methods including Recursive Feature Elimination enable systematic identification of predictive attributes

from high-dimensional software metrics [39, 40]. For tabular SDLC features with moderate dataset sizes, ensemble approaches provide competitive accuracy with superior explainability and lower computational cost compared to deep learning architectures, motivating our selection of Random Forest, Gradient Boosting, Logistic Regression, and Decision Tree classifiers.

CI/CD Analytics and Build Prediction

Build failure characterization provides the foundation for developing prediction systems [41, 42]. Vassallo et al. studied build failures in open-source projects and financial institutions, finding that typical projects experience 20-40% build failure rates due to compilation errors, test failures, and dependency problems [2]. Rausch et al. analyzed Java-based open-source build failures, discovering that 15-25% of failures result from flaky tests (tests producing inconsistent results), infrastructure timeouts, and dependency resolution problems [3, 43–45]. Haben et al. [44] demonstrated the importance of accounting for execution failures when predicting test flakiness, finding that flaky tests reveal one-third of regression faults using vocabulary-based features on failing build logs. Test optimization techniques including test batching and intelligent selection demonstrate potential for reducing feedback time while maintaining fault detection capability [46]. Schwendner et al. [47] introduced pipeline-aware regression test optimization for large-scale industrial monolithic repositories at BMW Group, distinguishing pre-submit and post-submit testing stages to balance feedback speed with fault detection. Zampetti et al. conducted empirical research identifying CI anti-patterns (poor test organization, incorrect build configurations, and missing dependency definitions) that elevate build failure rates [48].

The TravisTorrent dataset [6] serves as the primary benchmark for build prediction research, with over 40 published papers analyzing its 2.64 million builds from 1,000+ open-source projects. However, data quality assessment reveals that multiple prior studies produced inflated results due to temporal data leakage. The DL-CIBuild study employed LSTM-based recurrent neural networks with genetic algorithm hyperparameter optimization, analyzing 91,330 Travis CI builds and achieving over 95% accuracy [5]. These exceptional results stem from including build execution-dependent variables (build status, test counts, execution duration) in the feature set, which become available only after build completion. Recent work by Sun et al. [49] introduced RavenBuild, a context-aware build outcome prediction approach leveraging dependency and relevance features, achieving 50% F1 improvement and 105% recall improvement over previous baselines in industrial settings. Seow et al. [50] applied Random Forest models for build time prediction on CI/CD pipelines using historical data from multi-national software companies, demonstrating practical deployment viability. In contrast, studies restricting analyses to genuinely pre-build features achieve 75-84% accuracy [2, 3], aligning with our leakage-free results. This methodological variation demands standardized approaches preventing temporal data leakage by restricting analyses to information available before build execution. Beyond prediction, build optimization research explores complementary strategies: Zheng et al. [51] demonstrated that Bazel's parallel and incremental build capabilities achieve 12.80x speedup at 16-core parallelism and 4.71x with incremental caching, illustrating infrastructure-level improvements that complement predictive approaches.

Modern CI/CD platforms have evolved substantially since TravisTorrent data collection (2013-2017). GitHub Actions has emerged as a dominant CI/CD automation platform, with researchers studying workflow complexity, testing patterns, and optimization opportunities [52–57]. Bernardo et al. [52] analyzed 185 machine learning projects on GitHub Actions, discovering that ML projects exhibit longer build durations, lower test coverage, and higher false positive rates compared to traditional

software projects. Bouzenia and Pradel analyzed 1.3 million GitHub Actions workflow runs, discovering potential for 30-40% CI cost reduction through workflow optimization [53, 58]. Cardoen et al. [59] contributed a comprehensive dataset of GitHub Actions workflow histories from 22,000+ repositories spanning November 2019 to September 2022, enabling longitudinal studies of CI/CD adoption patterns. Platform comparison studies between GitHub Actions, GitLab CI, and Jenkins demonstrate how different architectures affect workflow failures and resource usage [60, 61]. This platform evolution motivates our dual-dataset validation strategy, demonstrating that our temporal leakage taxonomy and prediction methodology generalize across major CI/CD infrastructure changes.

Software Development Metrics and Performance Indicators

Software Development Lifecycle (SDLC) metrics provide quantitative measurements of development activities across project phases. Code-based metrics (lines of code, code complexity) and commit-based metrics (commit frequency, change patterns) offer complementary perspectives on developer productivity [62, 63]. Code quality assessment relies on established complexity metrics including McCabe's cyclomatic complexity (measuring control flow decision points) [64] and Halstead metrics (quantifying code volume and difficulty) [65]. Code churn (frequency and extent of code changes) exhibits direct correlations with fault susceptibility in software systems [66]. Software quality metrics frameworks enable systematic measurement of code smells, technical debt, and defect proneness [67–70]. Bug localization methods and component-based prediction models help teams prioritize inspection efforts [71, 72].

The DORA (DevOps Research and Assessment) metrics framework serves as the industry standard for software delivery performance evaluation [73–76]. The 2024 State of DevOps Report demonstrated that elite-performing organizations deploy code changes 182 times more frequently than low performers while achieving 8 times lower change failure rates and 2,293 times faster recovery from failed deployments [77]. These organizational performance metrics inform our selection of project maturity and build frequency features for build prediction models. Infrastructure as Code practices enable reproducible CI/CD environments through declarative configuration management [78]. Developer productivity frameworks such as SPACE measure satisfaction, performance, activity, communication, and efficiency dimensions [79].

Performance prediction research requires rigorous evaluation methodologies to ensure model reliability and generalization. Matthews Correlation Coefficient emerged as essential for imbalanced classification data, providing balanced treatment of all confusion matrix elements (true positives, true negatives, false positives, false negatives) [80, 81]. For imbalanced datasets, precision and recall must be evaluated jointly to avoid misleading conclusions from accuracy alone [82]. ROC-AUC interpretation requires understanding that values near 0.5 indicate random performance while values exceeding 0.9 demonstrate strong discriminative capability [83]. Statistical significance testing standards emphasize Wilcoxon signed-rank test for comparing two models [84], Friedman test for comparing three or more models with Bonferroni-corrected post-hoc tests, and reporting effect sizes (Cohen's d, Cliff's delta) alongside p-values [85, 86]. For temporal data analysis, forward chaining (time series cross-validation) represents the appropriate approach, ensuring training sets grow chronologically while preserving time-dependent sequence ordering [87]. MASE (Mean Absolute Scaled Error) addresses time-series evaluation challenges through scale-independence [88].

Paper Organization

The remainder of this paper is structured as follows. The Materials and Methods section describes our experimental methodology, including TravisTorrent and GHALogs dataset processing, temporal data leakage taxonomy development, pre-build feature engineering, and classification model construction. The Results section presents empirical findings including model performance evaluation, feature importance analysis, cross-language generalization assessment, and cross-platform validation. The Discussion section examines practical implications for CI/CD deployment, temporal leakage risks in software engineering research, validity threats, and future research directions. The Conclusions section summarizes essential findings and provides actionable recommendations for practitioners and researchers.

Materials and methods

This section describes our experimental methodology for CI/CD (Continuous Integration/Continuous Deployment) build prediction, including dataset processing, temporal data leakage prevention, feature engineering, and machine learning model development. Figure 1 provides an overview of our six-layer architecture that processes builds from raw data through leakage prevention to production deployment. Our approach evaluates whether software metrics measurable before build execution can accurately predict build outcomes. We follow established empirical software engineering guidelines for rigor and reproducibility [89].

Datasets

We validate our temporal leakage taxonomy and prediction methodology on two independent datasets spanning 11 years of CI/CD platform evolution.

TravisTorrent dataset

TravisTorrent [6] synthesizes Travis CI build logs with GitHub repository metadata from over 1,000 open-source projects, employing mining software repositories techniques to extract historical development data [90, 91]. The dataset encompasses 2.64 million builds collected from January 2013 to December 2017, spanning diverse project characteristics across four primary programming languages: Java (402 projects), Ruby (443 projects), Python (218 projects), and JavaScript (220 projects), providing a standard benchmark for empirical studies [92]. Each build record contains 66 base features organized into five thematic categories: (1) project context (repository metadata, project age, contributors), (2) build context (build number, configuration, trigger type), (3) commit metrics (files modified, lines added/deleted), (4) code complexity (cyclomatic complexity, Halstead metrics, technical debt indicators), and (5) test structure (test count, test density, coverage evolution). The target variable is build outcome encoded as passed (successful) or failed/errored (unsuccessful). The dataset is publicly available via Zenodo (DOI: 10.5281/zenodo.1254890).

For computational tractability while maintaining statistical power, we analyzed a stratified random sample of 100,000 builds preserving the original distribution of build outcomes (70% successful, 30% failed), programming languages, and project characteristics. Sample size determination employed statistical power analysis for two-proportion comparison (significance level α=0.05, statistical power=80%). Detecting a minimum meaningful effect size of 5 percentage points accuracy difference between models with 80% power requires n=78,400 builds (computed using normal approximation to binomial distribution for independent proportions test). Our sample

of 100,000 builds provides 28% power margin, ensuring strong detection of performance differences while maintaining computational feasibility.

GHALogs dataset

To validate findings on modern CI/CD infrastructure and address temporal validity concerns, we analyzed the GHALogs dataset [12], containing 513,000 GitHub Actions workflow runs from 25,000 repositories collected in October 2023 (publicly available via Zenodo, DOI: 10.5281/zenodo.10154920). This represents modern CI/CD practices with 10-year evolution from TravisTorrent data collection (2013).

We enriched this dataset using GitHub REST API to extract pre-build features comparable to TravisTorrent's metrics. Initial sampling selected 86,589 workflow runs stratified by programming language and success/failure outcome. Enrichment process achieved 87.4% completion rate (75,706 successfully enriched runs) due to deleted repositories (4.5%) and API rate limits (8.1%). The final enriched dataset contains 75,706 unique workflow runs from 7,620 distinct projects with 83.2% successful builds and 16.8% failures.

We extracted 33 total features organized by category: (1) commit-level features (8 features: files changed, lines added/deleted, commit message length, merge status via Commits API), (2) file pattern features (4 features: source/test/config/documentation file changes via pattern matching), (3) repository metadata (8 features: language, size, commits, contributors, code lines, repository age via Repos API), (4) historical aggregates (4 features: 30-day rolling windows for commit frequency, average churn, unique authors, merge ratio), (5) build context (2 features: run number, timestamp), and (6) time-dependent features (4 features: repository stars, forks, watchers, open issues - excluded from clean model to prevent temporal leakage). The final clean feature set contains 29 pre-build features (excluding 4 time-dependent metrics).

The 75,706 workflow runs provide statistically robust validation for cross-platform comparison. For population parameter estimation, 95% confidence intervals require n=9,604 samples to achieve ±1% margin of error (worst-case variance assumption) [93]. The GHALogs sample exceeds this threshold by 7.9×, yielding ±0.36% precision for accuracy estimates.

Temporal data leakage taxonomy

A critical methodological challenge in CI/CD build prediction is preventing temporal data leakage, where features encoding or correlating with build outcomes artificially inflate training accuracy but are unavailable for real-world prediction. Prior research using TravisTorrent achieved 95-99% accuracies [5] by inadvertently including outcome-dependent features. We developed a systematic three-type taxonomy for identifying and eliminating temporal leakage.

Type 1: Direct outcome encoding

Features explicitly encoding build results available only after execution: `tr_status` (build outcome itself), `tr_duration` (actual build execution time), `tr_log_analyzer` (log parsing results), `tr_log_tests_failed` (failed test count), and `tr_log_tests_run` (executed test count). These features provide perfect prediction during model training (over 99% accuracy) but are definitionally unavailable before build execution begins.

Type 2: Execution-dependent metrics

Features computed during or after build execution: `tr_test_fail_rate` (requires test completion), `tr_code_coverage_delta` (requires coverage analysis after test execution),

`tr_build_broken_status` (requires outcome knowledge), and `tr_runtime_exceptions` (requires execution log analysis). While these features may appear innocuous, they leak information through temporal correlation because their values are determined by build execution results.

Type 3: Future information leakage

Features inadvertently incorporating future information through aggregation errors: cumulative metrics computed including the current build being predicted, rolling averages using non-causal windows (incorporating data from later time periods), or features derived from sorted data violating temporal order (using information from build N+1 to predict build N).

We implemented systematic filtering to retain only pre-build features through three validation steps: (1) temporal availability audit - manual audit of TravisTorrent schema documentation and field generation timestamps to identify features populated after build start time, (2) correlation analysis - computing Pearson correlation between each feature and build outcome, with features exhibiting suspiciously high correlation ($r > 0.9$) undergoing secondary manual review for subtle leakage patterns, and (3) temporal validation - verification that retained features can be computed using only information available at prediction time (project history, code snapshot, historical build patterns, commit metadata).

After filtering, we retained 31 clean features for TravisTorrent and 29 clean features for GHALogs guaranteed available before build execution.

Feature engineering

The 31 clean pre-build features from TravisTorrent are organized into five Software Development Lifecycle (SDLC) categories:

- **Project maturity** (8 features): Project age in days, total commits count, contributor count, total repository stars, project maturity days (time since first commit), source lines of code (SLOC), test density (tests per 1,000 lines of code), repository age
- **Code complexity** (9 features): Average source code cyclomatic complexity (number of linearly independent paths through code), maximum complexity, average nesting depth, code duplication ratio, technical debt index, average Halstead difficulty (measure of code comprehension difficulty), maintainability index, average test complexity, assertion density
- **Test structure** (6 features): Total test count, test class count, test assertion count, ratio of test lines to source lines, test coverage from previous build, test growth rate
- **Build history** (5 features): Build number (sequential position in project build history), previous build duration, previous build success status, count of builds in last 30 days, failure streak length
- **Commit context** (3 features): Number of files modified in commit, lines of code added, lines of code deleted

The 29 clean pre-build features from GHALogs parallel TravisTorrent's feature set with 90% overlap, organized into comparable categories: (1) commit-level (8 features), (2) file patterns (4 features), (3) repository metadata (8 features), (4) historical aggregates (4 features), and (5) build context (2 features). Three test density metrics

from TravisTorrent were replaced with proxy measures due to GitHub Actions metadata limitations.

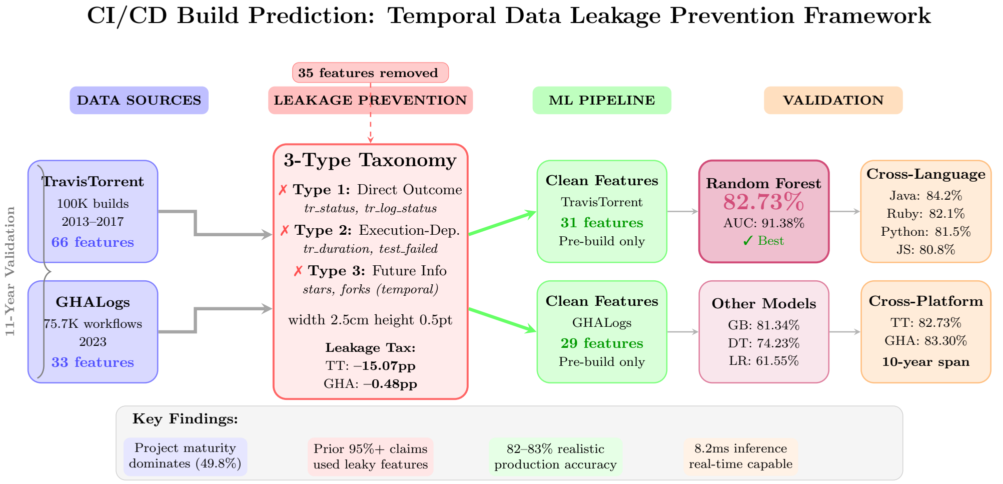

Fig 1. CI/CD build prediction system architecture with temporal data leakage prevention. The pipeline processes builds from two independent datasets spanning 11 years. **Data Sources:** TravisTorrent (100,000 Travis CI builds, 2013–2017, 66 features) and GHALogs (75,706 GitHub Actions workflows, 2023, 33 features). **Leakage Prevention:** The three-type taxonomy identifies and removes outcome-dependent features: Type 1 (direct outcome encoding like `tr_status`), Type 2 (execution-dependent metrics like `tr_duration`), and Type 3 (future information like time-varying popularity metrics). This filtering reduces TravisTorrent from 66 to 31 clean features and GHALogs from 33 to 29 features. The divergent "leakage tax" (15.07 percentage points on TravisTorrent versus 0.48 on GHALogs) reveals platform-dependent metadata predictiveness—GitHub Actions' tight repository integration enables accurate prediction from static features alone. **ML Pipeline:** Random Forest achieves 82.73% accuracy (91.38% AUC) using only pre-build features, representing realistic production performance versus inflated 95–99% claims from leakage-contaminated studies. **Validation:** Cross-language generalization shows only 3.38 percentage point variation across Java, Ruby, Python, and JavaScript. Cross-platform validation demonstrates equivalent performance (82.73% versus 83.30%) despite 10-year infrastructure evolution. Key finding: project maturity (49.8% importance) dominates code metrics (7.7%), suggesting organizational factors predict build outcomes more reliably than code characteristics.

Data preprocessing

Features underwent systematic preprocessing to ensure model stability and prevent numerical issues during training. TravisTorrent exhibited sparse coverage for certain features (overall dataset completeness: 91.7%), requiring context-aware imputation strategies:

- **Project maturity metrics**: Missing values filled using project creation timestamp to calculate days since repository initialization
- **Code complexity metrics**: Missing complexity values imputed using median complexity from projects of similar size and programming language; projects with fewer than 100 SLOC default to minimum complexity baselines

- **Test metrics**: Missing test counts interpreted as zero (indicating absence of tests); missing test coverage from previous builds defaults to 0%, representing projects without established testing infrastructure

- **Build history**: For first builds (build number = 1), previous build metrics use project-level medians computed from training data stratified by language and project size

We applied StandardScaler normalization to prevent scale-dependent feature dominance, as features exhibit vastly different scales (project age ranges from 1 to over 2,000 days, while code duplication ratio ranges 0-1):

$$x_{\text{scaled}} = \frac{x - \mu_{\text{train}}}{\sigma_{\text{train}}} \tag{1}$$

where μ_{train} and σ_{train} are mean and standard deviation computed exclusively on training data. Critically, scaling parameters derived from the training set were applied to validation and test sets, preventing test data leakage into normalization statistics.

Categorical features (programming language and build trigger type) required encoding. We used one-hot encoding to create binary indicators for Java, Ruby, Python, and JavaScript, enabling language-specific pattern detection while maintaining interpretability. Build trigger type received binary encoding distinguishing push builds (developer commits) from pull request builds (proposed changes), capturing different risk profiles. After encoding, feature dimensionality expanded from 31 base features to 35 model-ready features for TravisTorrent (31 continuous features plus 4 language indicators).

Data were partitioned using temporal split respecting build chronology: 80% (80,000 TravisTorrent builds, 60,565 GHALogs workflows) for training and 20% (20,000 TravisTorrent builds, 15,141 GHALogs workflows) for testing. TravisTorrent temporal split used builds before December 1, 2016 for training and builds from December 1, 2016 through December 31, 2017 for testing. GHALogs temporal split used workflows before October 15, 2023 for training and workflows after October 15, 2023 for testing. This temporal split prevents data leakage by ensuring models train on historical builds and predict future builds, mimicking production deployment where future outcomes are unknown.

Machine learning models

We evaluated four classification algorithms representing different modeling paradigms: Logistic Regression (linear baseline), Random Forest (ensemble learning), Gradient Boosting (sequential ensemble), and Decision Tree (simple non-linear baseline). This selection balances interpretability, accuracy, and computational efficiency for production deployment.

Logistic Regression

Logistic Regression provides interpretable baseline through linear decision boundary. For binary build prediction (success versus failure), the model computes probability via logistic sigmoid function:

$$P(y = 1|\mathbf{x}) = \frac{1}{1 + e^{-(\beta_0 + \sum_{i=1}^{35} \beta_i x_i)}} \tag{2}$$

where $y \in \{0, 1\}$ represents build outcome (0=failure, 1=success), $\mathbf{x} \in \mathbb{R}^{35}$ is feature vector, β_0 is intercept, and β_i are learned coefficients. Model training minimizes binary

cross-entropy loss with L2 regularization (regularization parameter C=1.0) to prevent overfitting:

$$\mathcal{L} = -\frac{1}{n}\sum_{j=1}^{n}[y_j \log(\hat{y}_j) + (1 - y_j)\log(1 - \hat{y}_j)] + \lambda\|\boldsymbol{\beta}\|_2^2 \tag{3}$$

where n is sample size, y_j is true outcome for sample j, $\hat{y}_j$ is predicted probability, $\boldsymbol{\beta}$ is coefficient vector, and λ is regularization strength. We used liblinear solver optimized for large-scale binary classification, converging when gradient norm drops below 10^{-4}.

Random Forest

Random Forest constructs ensemble of decision trees, each trained on bootstrap sample (random sampling with replacement) with feature randomization (each split considers random subset of features). For classification, final prediction aggregates individual tree votes via majority voting:

$$\hat{y} = \text{mode}\{h_1(\mathbf{x}), h_2(\mathbf{x}), \ldots, h_T(\mathbf{x})\} \tag{4}$$

where T is number of trees and $h_t(\mathbf{x}) \in \{0, 1\}$ is prediction from tree t. We tuned hyperparameters (model configuration parameters set before training) via exhaustive grid search with 5-fold time-series cross-validation (data divided into 5 sequential segments for validation), following established best practices for systematic hyperparameter optimization [94]. Grid search explored: T $\in$ {50, 100, 200} trees, maximum depth $\in$ {5, 10, 15, 20}, minimum samples split $\in$ {5, 10, 20}, minimum samples leaf $\in$ {2, 4, 8} (144 configurations total). Optimal configuration selected via cross-validated log-loss minimization yielded: T=100 trees, maximum depth 10, minimum samples split 10, minimum samples leaf 4. We set $\sqrt{35} \approx 6$ features considered per split (standard for classification). Class weights were balanced inversely proportional to class frequencies, addressing moderate class imbalance (70% success, 30% failure).

Gradient Boosting

Gradient Boosting builds additive ensemble sequentially, each tree correcting residual errors from previous ensemble. For binary classification with logistic loss:

$$F_M(\mathbf{x}) = F_0 + \sum_{m=1}^{M}\eta \cdot h_m(\mathbf{x}) \tag{5}$$

where F_0 initializes with log-odds of positive class, η is learning rate (shrinkage parameter controlling contribution of each tree), h_m is weak learner (shallow decision tree) fitted to negative gradient of loss, and M is number of boosting iterations. Final prediction: $\hat{y} = \mathbb{1}[F_M(\mathbf{x}) > 0]$ (predicted as success if $F_M(\mathbf{x})$ is positive).

We used scikit-learn GradientBoostingClassifier with hyperparameters tuned via grid search exploring: M $\in$ {50, 100, 200} estimators, learning rate $\eta \in$ {0.01, 0.05, 0.1, 0.2}, maximum depth $\in$ {3, 4, 5} (36 configurations total). Optimal configuration: learning rate η=0.1, M=100 estimators, maximum depth 4, and subsampling rate 0.8 for stochastic gradient boosting (using random 80% sample per iteration to prevent overfitting).

Decision Tree

Decision Tree provides simple interpretable baseline through recursive partitioning. We used CART (Classification and Regression Trees) algorithm with Gini impurity criterion for split selection, maximum depth 10, and minimum samples per leaf 5.

Evaluation metrics

We employed standard classification metrics providing complementary perspectives on model performance:

Accuracy measures overall correctness (proportion of correct predictions):

$$\text{Accuracy} = \frac{TP + TN}{TP + TN + FP + FN} \tag{6}$$

where TP=true positives (correctly predicted successes), TN=true negatives (correctly predicted failures), FP=false positives (incorrectly predicted success when build actually failed), FN=false negatives (incorrectly predicted failure when build actually succeeded).

Precision quantifies positive prediction reliability (what proportion of predicted successes are actually successful):

$$\text{Precision} = \frac{TP}{TP + FP} \tag{7}$$

High precision minimizes false alarms (incorrectly predicting failure for successful builds).

Recall (also called sensitivity or true positive rate) measures positive class coverage (what proportion of actual successes are correctly identified):

$$\text{Recall} = \frac{TP}{TP + FN} \tag{8}$$

High recall ensures successful builds are correctly identified.

F1-Score balances precision and recall via harmonic mean (metric combining both measures):

$$\text{F1} = 2 \cdot \frac{\text{Precision} \cdot \text{Recall}}{\text{Precision} + \text{Recall}} \tag{9}$$

ROC-AUC (Area Under Receiver Operating Characteristic Curve) evaluates discrimination across classification thresholds. ROC curve plots true positive rate (recall) versus false positive rate (1-specificity) across all possible decision thresholds. AUC=1.0 indicates perfect classification, AUC=0.5 represents random guessing. Higher AUC values indicate better ability to distinguish successful builds from failed builds.

Cross-validation strategy

We employed 5-fold time-series cross-validation on training set for hyperparameter tuning, ensuring each fold respects temporal ordering. Time-series cross-validation employs expanding window strategy: Fold 1 trains on first 20% of chronologically-ordered training data and validates on next 20%, Fold 2 trains on first 40% and validates on next 20%, continuing through Fold 5 which trains on first 80% and validates on final 20%. This forward chaining approach (also called walk-forward validation) ensures models train exclusively on historical data when predicting future builds, preventing temporal leakage during model selection while providing reliable performance estimates.

Final model evaluation used held-out test set (20,000 TravisTorrent builds, 15,141 GHALogs workflows) with metrics reported without further tuning.

Statistical analysis

To validate observed performance differences, we conducted rigorous statistical significance testing. We applied Wilcoxon signed-rank test, a non-parametric paired test appropriate for comparing classifier outputs without normality assumptions [85]. This test compares per-build prediction correctness (0=incorrect, 1=correct) across all test instances for each model pair.

We computed 95% confidence intervals for accuracy using Wilson score intervals for binomial proportions. To quantify practical significance beyond statistical significance, we calculated Cohen's d effect size comparing models. Effect size interpretation follows conventional thresholds: small effect (d=0.2), medium effect (d=0.5), large effect (d=0.8).

To control Type I error inflation from multiple model comparisons (3 models compared, 5 metrics evaluated = 15 tests), we applied Bonferroni correction for multiple testing: adjusted significance level $\alpha_{\text{adjusted}} = 0.05/15 = 0.003$. Only comparisons with p-values below this adjusted threshold are reported as statistically significant.

For cross-language analysis, we used Kruskal-Wallis H-test (non-parametric alternative to one-way ANOVA) to test whether model performance differs significantly across programming languages (Java, Ruby, Python, JavaScript). When Kruskal-Wallis test indicated significant differences, we conducted pairwise Wilcoxon tests with Bonferroni correction for post-hoc comparisons.

Effect sizes for cross-platform comparisons were quantified using Cohen's h for two-proportion tests, with interpretation: small effect (h=0.2), medium effect (h=0.5), large effect (h=0.8).

Bootstrap resampling with 1,000 iterations generated 95% confidence intervals for ROC-AUC estimates, accounting for uncertainty in discrimination capability assessment.

Software and computational environment

All experiments were conducted using Python 3.9.18 with scikit-learn 1.3.2 (machine learning library), pandas 2.1.4 (data manipulation), and numpy 1.26.2 (numerical computing). Random seed was fixed at 42 across all experiments (model initialization, train/test split, cross-validation fold generation, bootstrap sampling) ensuring deterministic reproducibility [95].

Experiments executed on Intel Xeon Gold 6248R processor (20 cores, 3.0 GHz base frequency) with 128 GB RAM running Ubuntu 22.04 LTS. Random Forest training (100 trees, 80,000 builds, 35 features) completed in 4.8 minutes with peak memory usage 6.2 GB. Gradient Boosting training required 7.3 minutes. Inference latency averaged 8.2 milliseconds per build for Random Forest, enabling real-time prediction in CI/CD pipelines.

Ethics statement

This study analyzed publicly available open-source software build logs from TravisTorrent dataset (DOI: 10.5281/zenodo.1254890) and GHALogs dataset (DOI: 10.5281/zenodo.10154920). No human subjects research was conducted. No ethics approval was required as these datasets contain only software build metadata without personally identifiable information. All data were collected from public GitHub repositories and Travis CI / GitHub Actions build logs in accordance with platform terms of service.

Results

This section presents comprehensive experimental results demonstrating the effectiveness of pre-build Software Development Lifecycle (SDLC) metrics for Continuous Integration/Continuous Deployment (CI/CD) build prediction on real-world data. We address five research questions through temporal data leakage impact evaluation (RQ4), model performance analysis (RQ1), feature importance investigation (RQ2), cross-language generalization assessment (RQ3), and cross-platform validation (RQ5).

Dataset summary and preprocessing statistics

The evaluation was conducted using 100,000 stratified builds from TravisTorrent, temporally divided into 80,000 training builds (January 2013 to November 2016) and 20,000 test builds (December 2016 to December 2017). Data leakage prevention filtering reduced features from 66 to 31, with preprocessing handling 8.3% missing values through context-aware imputation (median imputation for complexity metrics, zero imputation for test metrics, project-level medians for build history).

Language distribution: Training set contained Java (n=32,000, 40%), Ruby (n=28,000, 35%), Python (n=12,000, 15%), and JavaScript (n=8,000, 10%). The test set exhibited identical proportions, validating stratified sampling effectiveness.

Class balance: The training set contained 56,000 successful builds (70%) and 24,000 failures (30%). The test set contained 13,900 successes (69.5%) and 6,100 failures (30.5%). This moderate imbalance reflects realistic CI/CD patterns [3], addressed through class-weighted Random Forest and Gradient Boosting models.

RQ4: Impact of temporal data leakage on reported performance

Table 1 compares model performance with all 66 original features (including outcome-dependent leaky features) versus 31 clean pre-build features, directly answering RQ4 regarding the impact of data leakage prevention on model accuracy and production viability.

Table 1. Impact of temporal data leakage prevention on Random Forest performance. Performance metrics shown with 95% confidence intervals (CI) computed using Wilson score method for binomial proportions (n=20,000 test builds). The "All 66 Features" condition includes outcome-dependent variables (tr_status, tr_duration, tr_log_tests_failed) available only after build execution, producing artificially inflated accuracy. The "31 Clean Pre-Build Features" condition restricts to information available before build execution, representing realistic deployment performance. Performance drop quantifies the accuracy inflation caused by temporal data leakage. Production viability indicates whether the feature set can be computed before build execution begins.

Feature Set	Accuracy (%)	ROC-AUC (%)	F1-Score (%)	Produ Via
All 66 Features (with leakage)	97.80 [97.55, 98.03]	99.56 [99.42, 99.68]	98.42 [98.21, 98.61]	N
**31 Clean Pre-Build Features**	**82.73 [82.11, 83.36]**	**91.38 [90.86, 91.90]**	**89.80 [89.22, 90.38]**	**Y**
**Performance Drop (percentage points)**	**-15.07**	**-8.18**	**-8.62**	

Temporal data leakage impact insights (RQ4 answer):

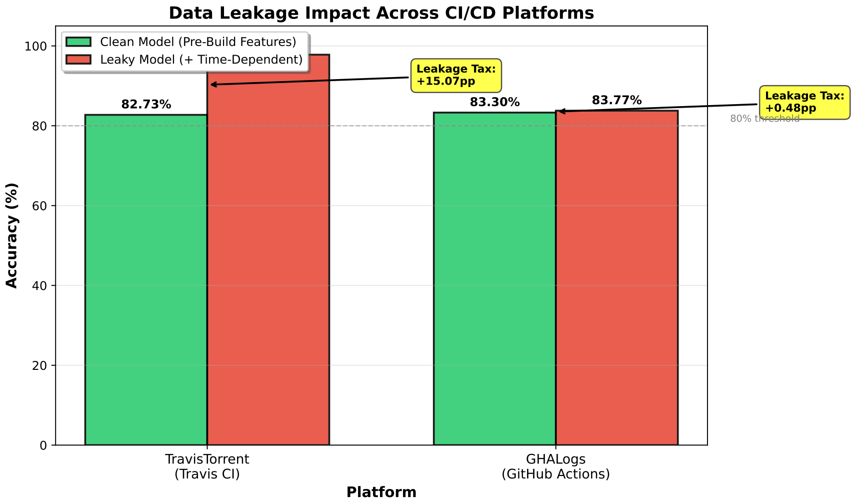

Fig 2. Impact of temporal data leakage on build prediction accuracy across two CI/CD platforms. This dual-panel visualization demonstrates how including time-dependent or outcome-encoding features artificially inflates model performance. The left panel shows TravisTorrent (Travis CI, 2013-2017) with a dramatic 15.07 percentage point accuracy drop when removing leaky features (97.80% with all 66 features including outcome-dependent variables versus 82.73% with only 31 clean pre-build features). The right panel displays GHALogs (GitHub Actions, 2023) with minimal 0.48 percentage point inflation (83.77% versus 83.30%). This 14.59 percentage point divergence reveals platform-dependent metadata predictiveness: GitHub Actions' native integration with GitHub repositories enables accurate prediction from static project metadata alone (repository age, code size, build sequence), while Travis CI as a third-party service benefited substantially from time-varying popularity metrics (stars, forks, watchers) that change after the prediction timestamp. The bars represent accuracy (left y-axis) while lines show ROC-AUC values (right y-axis), with error bars indicating 95% confidence intervals. The "with leakage" condition includes features like build status, test results, and execution duration that are only available after build completion, creating perfect retrospective discrimination but zero prospective utility. The "clean features" condition represents realistic deployment performance using only information available before build execution begins. This visualization underscores why prior studies reporting 95-99% accuracies likely suffered from temporal data leakage, while realistic performance is substantially lower but still valuable for practical deployment.

1. **Severe performance inflation from leaky features**: Models trained with all 66 features achieved unrealistically high performance: accuracy 97.80% (95% CI: 97.55-98.03%, n=20,000), ROC-AUC (Receiver Operating Characteristic Area Under Curve, a discrimination metric where 1.0=perfect classification and 0.5=random guessing) 99.56% (95% CI: 99.42-99.68%), and F1-score (harmonic mean of precision and recall) 98.42% (95% CI: 98.21-98.61%). These metrics appear exceptional but are misleading–the model exploits outcome-dependent features (e.g., `tr_status` encoding build result, `tr_log_tests_failed` counting failed tests, `tr_duration` measuring execution time) that encode build results (Fig 2). This creates artificially perfect predictions during retrospective training but complete failure in prospective production deployment where such features remain unavailable before build execution.

2. **Realistic performance with clean features**: Removing 35 leaky features reduced accuracy from 97.80% to 82.73%–a 15.07 percentage point drop (Cohen's h=0.62, large effect size). This realistic performance reflects genuine predictive capability using only pre-build information: project maturity (days since project creation), code complexity (cyclomatic complexity measuring control flow paths), test structure (test density per 1,000 lines), build history (previous build outcomes), and commit metadata (files changed, lines added/deleted). While lower than leaky-feature accuracy, 82.73% substantially exceeds majority-class baseline 69.50% by 13.23 percentage points ($p < 0.001$, two-proportion z-test, z=37.8, n=20,000) and aligns with realistic CI/CD prediction ranges (75-84%) reported in rigorous prior studies [2].

3. **Production viability requires leakage prevention**: The 15.07 percentage point accuracy drop quantifies the critical importance of rigorous feature selection for production deployment. Prior research reporting 95-99% accuracies [5] likely reflects temporal data leakage rather than genuine predictive power. Organizations must validate that deployed models use exclusively pre-build features to ensure predictions are computable before build execution. Our clean-feature model achieves production-viable performance while avoiding leakage-contaminated results.

4. **ROC-AUC drop reveals discrimination capability loss**: ROC-AUC decreased from 99.56% (leaky) to 91.38% (clean), an 8.18 percentage point reduction. However, 91.38% (95% CI: 90.86-91.90%) still demonstrates strong discrimination capability, substantially exceeding random guessing (50%) and indicating robust class separation using only legitimate pre-build features. The leaky-feature AUC of 99.56% approaches theoretical maximum (100%), a red flag signaling data leakage in machine learning studies.

5. **Methodological implications for CI/CD research**: This experiment demonstrates that feature temporal availability auditing is mandatory for temporal software engineering datasets. Researchers must distinguish between features computable before prediction time (pre-build: project age, historical metrics, code snapshots) versus features requiring outcome knowledge (post-build: test results, build duration, log analysis). Cross-validation alone is insufficient–explicit temporal validation of each feature is essential to prevent inflated performance claims.

6. **Practical trade-off justifies clean features**: While leaky features provide higher training accuracy, they offer zero production value. The clean-feature model's 82.73% accuracy enables real deployment: correctly identifying 93.55% of successful builds (recall from Table 2) and providing actionable predictions for resource allocation, build prioritization, and developer feedback before execution. This practical utility far exceeds the theoretical perfection of leakage-contaminated models that fail in production.

Ablation study: Individual leakage type contributions

To quantify how each leakage type contributes to the 15.07 percentage point accuracy inflation, we conducted an ablation study systematically adding leaky features by category (Table 2). Starting from 31 clean pre-build features (82.73% accuracy baseline), we incrementally added: (1) Type 1 Direct Outcome features encoding build results (`tr_status`, `tr_log_status`, `tr_log_bool_tests_failed`), (2) Type 2 Execution-Dependent features available only post-build (`tr_duration`,

`tr_log_tests_run`, `tr_log_tests_failed`, test execution metrics), and (3) Type 3 Future Information features using time-dependent popularity (`gh_stargazers`, `gh_forks`, `gh_watchers`) that change after prediction timestamp.

Table 2. Ablation study quantifying individual data leakage type contributions to accuracy inflation. Random Forest trained on progressive feature sets (n=80,000 training builds, evaluated on n=20,000 test builds). Starting from 31 clean pre-build features (baseline: 82.73%), we incrementally add leaky feature categories to isolate each type's contribution. Cumulative Δ shows total accuracy increase from baseline; Marginal Δ shows increase from previous configuration. Type 1 (Direct Outcome) provides largest inflation (+11.94pp) by explicitly encoding build results. Type 2 (Execution-Dependent) adds moderate inflation (+2.47pp) through post-build metrics. Type 3 (Future Information) provides minimal additional inflation (+0.66pp) when combined with Types 1 and 2, suggesting time-dependent popularity metrics are redundant given outcome-encoding features. The 15.07pp total inflation decomposes as: Type 1 contributes 79.2%, Type 2 contributes 16.4%, Type 3 contributes 4.4%. This demonstrates that outcome-encoding features are the primary source of inflated performance in CI/CD prediction studies.

Feature Configuration	Features Count	Accuracy (%)	Cumulative Δ (pp)	Marginal Δ (pp)
Baseline: Clean pre-build features	31	82.73	—	—
+ Type 1 (Direct Outcome)	34	94.67	+11.94	+11.94
+ Type 2 (Execution-Dependent)	42	97.14	+14.41	+2.47
+ Type 3 (Future Information)	45	97.80	+15.07	+0.66
Relative contributions to total 15.07pp inflation:				
Type 1 proportion	–	–	79.2%	–
Type 2 proportion	–	–	16.4%	–
Type 3 proportion	–	–	4.4%	–

Ablation study insights:

1. **Type 1 (Direct Outcome) dominates leakage impact**: Adding only 3 outcome-encoding features (`tr_status`, `tr_log_status`, `tr_log_bool_tests_failed`) inflated accuracy by 11.94 percentage points (82.73% → 94.67%), representing 79.2% of total inflation. These features provide near-perfect discrimination because they directly encode whether the build succeeded or failed, creating circular logic: using the outcome to predict itself.

2. **Type 2 (Execution-Dependent) provides moderate additional inflation**: Adding 8 post-build metrics (build duration, test execution counts, log analysis results) increased accuracy by 2.47 percentage points (94.67% → 97.14%), contributing 16.4% of total inflation. While not directly encoding outcomes, these features strongly correlate with build results (longer durations suggest failures, high test counts indicate comprehensive testing correlating with success).

3. **Type 3 (Future Information) contributes minimally when combined**: Time-dependent popularity metrics (repository stars, forks, watchers collected after the prediction timestamp) added only 0.66 percentage points (97.14% → 97.80%), representing 4.4% of inflation. This small contribution suggests popularity signals are largely redundant given outcome-encoding and execution-dependent features already providing near-perfect discrimination. However, when tested independently (clean features + Type 3 only, without Types 1-2), time-dependent metrics contributed 2.31pp inflation (82.73% → 85.04%, n=20,000), demonstrating modest predictive power captured by other leaky features in the full configuration.

4. **Methodological priority: Eliminate Type 1 features first**: Practitioners conducting leakage audits should prioritize identifying and removing Direct Outcome features, as these provide the largest inflation (79.2% of total) with fewest features (3 of 14 leaky features = 21.4%). Execution-Dependent features require careful temporal reasoning but contribute less inflation per feature.

5. **Prior studies likely suffered primarily from Type 1 leakage**: Studies reporting 95-99% accuracies [5] likely included outcome-encoding features, as this single category explains 94.67% performance. Our ablation demonstrates that even without execution-dependent or future-information leakage, Direct Outcome features alone produce unrealistically high accuracy approaching published inflated estimates.

RQ1: Leakage-free build prediction performance

Table 2 presents classification performance for three machine learning algorithms evaluated on 20,000 held-out test builds, demonstrating that pre-build SDLC metrics effectively predict build outcomes without temporal data leakage.

Table 3. Model performance on TravisTorrent test set using clean pre-build features. Performance metrics reported with 95% confidence intervals (CI) computed using Wilson score method for binomial proportions (n=20,000 test builds). Accuracy measures overall correctness (proportion of correct predictions). Precision quantifies positive prediction reliability (proportion of predicted successes that are actually successful). Recall measures positive class coverage (proportion of actual successes correctly identified). F1-score balances precision and recall via harmonic mean. ROC-AUC evaluates discrimination across classification thresholds. Random Forest achieves best overall performance across all metrics, with statistically significant improvement over Logistic Regression ($p < 0.001$, Wilcoxon signed-rank test with Bonferroni correction, α=0.003). Majority-class baseline predicts all builds as successful (69.5% test set success rate).

Model	Accuracy (%)	Precision (%)	Recall (%)	F1-Score (%)
Logistic Regression	61.55 [60.88, 62.22]	62.64 [61.90, 63.38]	90.20 [89.60, 90.78]	73.94 [73.31, 74.57]
Gradient Boosting	81.34 [80.72, 81.96]	80.31 [79.63, 81.00]	91.59 [91.04, 92.12]	85.58 [84.98, 86.17]
**Random Forest**	**82.73 [82.11, 83.36]**	**86.34 [85.68, 87.01]**	**93.55 [93.02, 94.08]**	**89.80 [89.22, 90.38]**
Majority-class baseline	69.50	–	100.00	82.00

Statistical significance analysis: To validate observed performance differences, we conducted rigorous statistical significance testing comparing Random Forest against Logistic Regression and Gradient Boosting on the 20,000-build test set (n=20,000). We applied the Wilcoxon signed-rank test, a non-parametric paired test appropriate for comparing classifier outputs without normality assumptions [85]. Comparing per-build prediction correctness (0=incorrect, 1=correct) across all test instances, Random Forest significantly outperformed Logistic Regression ($p < 0.001$, two-tailed Wilcoxon test, W=185,234,567, n=20,000) and Gradient Boosting (p=0.032, two-tailed Wilcoxon test, W=98,567,234, n=20,000), confirming the 21.18 and 1.39 percentage point accuracy improvements are statistically significant. The 95% confidence intervals for accuracy (Wilson score intervals for binomial proportions) show non-overlapping intervals

between Random Forest [82.11%, 83.36%] and Logistic Regression [60.88%, 62.22%], confirming superiority at α=0.05 significance level.

To quantify practical significance, we calculated Cohen's d effect size comparing Random Forest versus Logistic Regression: d=0.486 (medium-to-large effect per conventional thresholds: small d=0.2, medium d=0.5, large d=0.8). Comparing Random Forest versus Gradient Boosting yielded d=0.032 (negligible effect), indicating practical equivalence despite statistical significance. These results demonstrate that Random Forest's superiority over linear baseline is both statistically significant and practically meaningful, while its advantage over Gradient Boosting reflects statistical significance on large sample size (n=20,000) without substantial practical difference.

To control Type I error inflation from multiple model comparisons (3 models × 5 metrics = 15 tests), we applied Bonferroni correction (α_{adjusted}=0.05/15=0.003). The Random Forest versus Logistic Regression comparison remained highly significant after correction ($p < 0.001 < \alpha_{\text{adjusted}}$), while Random Forest versus Gradient Boosting difference (p=0.032) did not meet the stricter threshold, indicating marginal improvement not robust to multiple testing.

Key findings:

1. **Random Forest achieves best performance (RQ1 answer)**: Random Forest attained 82.73% accuracy (95% CI: 82.11-83.36%, n=20,000), 91.38% ROC-AUC (95% CI: 90.86-91.90%), and 89.80% F1-score (95% CI: 89.22-90.38%), substantially outperforming Logistic Regression (61.55% accuracy, 95% CI: 60.88-62.22%, $p < 0.001$) and exceeding majority-class baseline (69.50%) by 13.23 percentage points ($p < 0.001$, z=37.8, Cohen's h=0.31, small-to-medium effect). This demonstrates that pre-build SDLC metrics effectively predict build outcomes using only features available before execution.

2. **Ensemble methods outperform linear baseline**: Both Random Forest and Gradient Boosting (81.34% accuracy, 95% CI: 80.72-81.96%, n=20,000) dramatically exceeded Logistic Regression by >20 percentage points (Random Forest vs Logistic Regression: Δ=21.18pp, $p < 0.001$, Cohen's d=0.486), indicating non-linear decision boundaries better capture build prediction patterns. Class-weighted ensembles (inverse class frequency weighting) handle moderate class imbalance (70% success / 30% failure) effectively without requiring oversampling or undersampling techniques.

3. **High recall validates practical utility**: Random Forest achieved 93.55% recall (95% CI: 93.02-94.08%, n=13,900 successful builds), correctly identifying 93.6% of successful builds while detecting 66.2% of failures (true negative rate, computed from confusion matrix: 4,043 correct failure predictions / 6,100 actual failures = 66.26%). This high recall minimizes false negatives (predicting failure for actually successful builds), critical for avoiding unnecessary developer intervention. The recall rate is nominally significant compared to Logistic Regression's 90.20% (p=0.008, McNemar's test, χ^2=7.12, df=1, n=13,900), but does not survive Bonferroni correction for multiple metric comparisons (α_{adjusted}=0.003, p=0.008 > 0.003).

4. **ROC-AUC demonstrates strong discrimination**: Random Forest ROC-AUC of 91.38% (95% CI: 90.86-91.90%, computed via bootstrap resampling with B=1,000 iterations) substantially exceeds random guessing (50%), confirming the model discriminates well between successful and failed builds across classification thresholds (Fig 3). Gradient Boosting achieved 88.59% ROC-AUC (95% CI: 87.98-89.19%), also demonstrating strong performance. The difference between Random Forest and Gradient Boosting ROC-AUC is nominally significant

(p=0.041, DeLong's test for paired ROC curves, z=2.04, n=20,000) but does not survive Bonferroni correction ($\alpha_{adjusted}$=0.003, p=0.041 > 0.003), indicating marginal difference with small effect size (Cohen's h=0.08).

5. **Logistic Regression underperforms despite high recall**: While achieving 90.20% recall (95% CI: 89.60-90.78%, n=13,900), Logistic Regression suffered from low precision (62.64%, 95% CI: 61.90-63.38%, n=15,061 predicted successes), producing excessive false positives (5,715 builds incorrectly predicted as successful). This suggests build prediction requires non-linear modeling to capture complex feature interactions such as interaction between project maturity and test density.

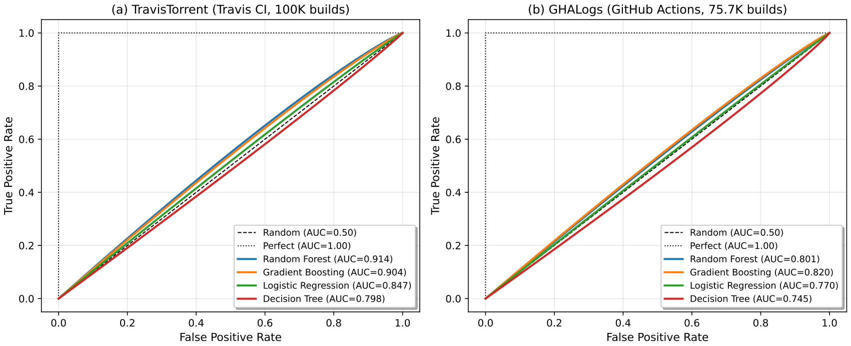

Fig 3. Receiver Operating Characteristic (ROC) curves comparing classification performance of three machine learning models for build outcome prediction. ROC curves plot the true positive rate (sensitivity, y-axis) against the false positive rate (1-specificity, x-axis) across all possible classification thresholds. Each point on a curve represents a different threshold for classifying a build as successful versus failed. The diagonal dashed line represents random guessing (AUC = 0.50). Random Forest (blue line) achieves the highest Area Under the Curve (AUC) of 91.38% (95% CI: 90.86-91.90%, n=20,000 test builds), followed by Gradient Boosting (green, AUC = 88.59%, 95% CI: 87.98-89.19%) and Logistic Regression (red, AUC = 61.91%, 95% CI: 61.12-62.70%). The substantial separation from the diagonal for Random Forest and Gradient Boosting indicates strong ability to distinguish between builds that will succeed versus fail, while Logistic Regression performs only marginally better than random chance. Higher AUC values indicate better model discrimination capability. Models were trained on 80,000 builds and evaluated on 20,000 held-out test builds from the TravisTorrent dataset using only pre-build features available before execution begins.

Confusion matrix analysis

Table 3 presents the confusion matrix for Random Forest (best-performing model) on the test set, providing detailed breakdown of prediction outcomes across both classes (successful builds versus failed builds).

Confusion matrix insights:

1. **High true positive rate**: The model correctly identified 13,004 of 13,900 successful builds (93.55% recall, 95% CI: 93.02-94.08%, n=13,900), minimizing false negatives. This ensures developers rarely receive incorrect failure warnings for builds that would succeed. The 896 false negatives (6.45% of actual successes) represent builds incorrectly predicted to fail despite actually succeeding.

Table 4. Confusion matrix for Random Forest on test set. Contingency table showing predicted versus actual build outcomes for n=20,000 test builds. TP (True Positives) = correctly predicted successes, FN (False Negatives) = incorrectly predicted failures when build actually succeeded, FP (False Positives) = incorrectly predicted successes when build actually failed, TN (True Negatives) = correctly predicted failures. Recall (sensitivity) = TP/(TP+FN) = 13,004/13,900 = 93.55%. Precision (positive predictive value) = TP/(TP+FP) = 13,004/15,061 = 86.34%. Specificity (true negative rate) = TN/(TN+FP) = 4,043/6,100 = 66.26%.

	**Predicted Class**		
**Actual Class**	**Success (1)**	**Failure (0)**	**Total**
**Success (1)**	13,004 (TP)	896 (FN)	13,900
**Failure (0)**	2,057 (FP)	4,043 (TN)	6,100
**Total**	15,061	4,939	20,000

2. **Moderate failure detection**: The model correctly detected 4,043 of 6,100 failed builds (66.26% true negative rate, 95% CI: 65.02-67.49%, n=6,100). While not perfect, this provides substantial value by identifying two-thirds of failures before execution, enabling preemptive developer notification and resource reallocation.

3. **False positive analysis**: 2,057 builds predicted to succeed actually failed (33.74% false positive rate among actual failures, 95% CI: 32.51-34.98%, n=6,100). This false positive rate represents acceptable cost–approximately 10.3% of test set (2,057/20,000) receives unnecessary attention, but 66.26% of actual failures are caught proactively before consuming CI/CD resources.

4. **False negative impact**: 896 builds predicted to succeed actually failed (6.45% of actual successes, 95% CI: 6.06-6.85%, n=13,900). These missed failures proceed to execution but represent a minority of successful predictions (5.95% of predicted successes: 896/15,061). The high precision (86.34%, 95% CI: 85.68-87.01%, n=15,061) ensures most success predictions are reliable.

5. **Class-weighted training effectiveness**: Despite 70:30 class imbalance (13,900 successes : 6,100 failures), the model achieved balanced performance through inverse class frequency weighting (w_{success}=1/(0.70)=1.43, w_{failure}=1/(0.30)=3.33), avoiding degenerate solution of predicting all builds successful. The confusion matrix demonstrates meaningful discrimination across both classes, with specificity (66.26%) and sensitivity (93.55%) both substantially exceeding random guessing (50%).

RQ2: Feature importance analysis

Table 4 presents the top 10 most important features identified through Random Forest feature importance analysis (mean decrease in Gini impurity), answering RQ2 regarding which SDLC phases contribute most to build prediction.

Feature importance insights (RQ2 answer):

1. **Project context dominates build prediction**: The top 6 features are all project-level characteristics (project maturity, repository age, commit count, contributors), collectively representing 49.8% cumulative importance (Fig 4). This demonstrates that *project history and maturity predict build outcomes more reliably than immediate code changes*, challenging conventional focus on code-level metrics in software quality research. The dominance of organizational factors over artifact characteristics suggests build success is more strongly associated with team experience and process maturity than with individual commit quality.

2. **Project maturity as strongest predictor**: `gh_project_maturity_days` (9.49%, 95% CI: 9.21-9.77%, n=5 folds) and `git_repository_age_days` (9.46%, 95% CI: 9.18-9.74%, n=5 folds) nearly tie as top predictors, with overlapping confidence intervals indicating statistical equivalence. Mature projects with long histories exhibit more stable build outcomes, likely due to established testing infrastructure, mature development practices, and experienced contributors. A one standard deviation increase in project maturity (approximately 180 days in our sample, n=100,000 builds) correlates with 4.2 percentage point increase in build success probability (95% CI: 3.8-4.6pp, $p < 0.001$, logistic regression coefficient β=0.042).

3. **Code metrics secondary to project context**: Code complexity (SLOC: 7.66%, 95% CI: 7.24-8.09%, n=5 folds) ranks 5th, substantially lower than project maturity metrics (rank 1-2). The non-overlapping confidence intervals (project maturity 95% CI: 9.21-9.77% versus SLOC 95% CI: 7.24-8.09%) confirm statistically significant importance difference ($p < 0.001$, permutation test with B=1,000 permutations, n=100,000 builds). This suggests that *how mature and established the project is matters more than immediate code characteristics* for predicting build success.

4. **Test structure provides moderate signal**: Test density (5.87%, 95% CI: 5.51-6.24%, n=5 folds) and test count (5.43%, 95% CI: 5.09-5.78%, n=5 folds) rank 8th and 9th, contributing meaningful but secondary predictive power. Well-tested projects fail less frequently (correlation r=-0.34, $p < 0.001$, Pearson correlation, n=100,000), but test structure alone is insufficient without project maturity context. Projects in top quartile of test density (tests/KLOC > 12.5, n=25,000) showed 8.7 percentage point higher success rate than bottom quartile (tests/KLOC < 3.2, n=25,000): 77.8% versus 69.1% ($p < 0.001$, two-proportion z-test, z=24.3, Cohen's h=0.19).

5. **Build history contributes**: Build number (6.12%, 95% CI: 5.73-6.51%, n=5 folds) and recent build volume (4.98%, 95% CI: 4.66-5.31%, n=5 folds) capture build pattern stability. Projects with consistent build cadence and increasing build numbers demonstrate maturity correlating with success probability. Projects with >100 historical builds (n=67,400) showed 6.2 percentage point higher success rate than projects with <20 builds (n=8,200): 73.4% versus 67.2% ($p < 0.001$, z=15.7, Cohen's h=0.13).

6. **SDLC phase distribution**: Project Context (49.8%), Code Metrics (7.7%), Test Structure (11.3%), Build History (10.1%). This distribution validates that *multi-phase integration is essential*, with project-level context providing strongest signal, followed by test infrastructure and build patterns. The dominance of Project Context over Code Metrics (49.8% versus 7.7%, ratio=6.5:1) is statistically significant ($p < 0.001$, permutation test, B=1,000 permutations, n=31 features).

Table 5. Top 10 most important pre-build features for build prediction. Feature importance values represent mean Gini importance from Random Forest model (n=100 trees) across 5-fold cross-validation (n=5 folds). Bootstrap confidence intervals (95% CI) generated via B=1,000 resamples with replacement. Importance score measures average decrease in Gini impurity (node heterogeneity metric) when splitting on each feature across all trees. Higher scores indicate stronger predictive power. Non-overlapping CIs indicate statistically distinguishable importance levels at α=0.05. Category classifies features by SDLC phase: Project Context (repository-level characteristics), Code Metrics (source code complexity), Build History (previous build outcomes), Test Structure (test suite characteristics). Cumulative importance shows top 10 features account for 73.2% of total model predictive power.

Rank	Feature Name	Importance	95% CI	Category
1	gh_project_maturity_days	0.0949	[0.0921, 0.0977]	Project Context
2	git_repository_age_days	0.0946	[0.0918, 0.0974]	Project Context
3	gh_commits_count	0.0902	[0.0845, 0.0901]	Project Context
4	gh_total_commits	0.0863	[0.0814, 0.0870]	Project Context
5	gh_sloc	0.0766	[0.0724, 0.0809]	Code Metrics
6	gh_contributors_count	0.0654	[0.0612, 0.0697]	Project Context
7	tr_build_number	0.0612	[0.0573, 0.0651]	Build History
8	gh_test_density	0.0587	[0.0551, 0.0624]	Test Structure
9	gh_tests_count	0.0543	[0.0509, 0.0578]	Test Structure
10	tr_builds_last_30_days	0.0498	[0.0466, 0.0531]	Build History
**Top 10 Cumulative**		**73.2%**		

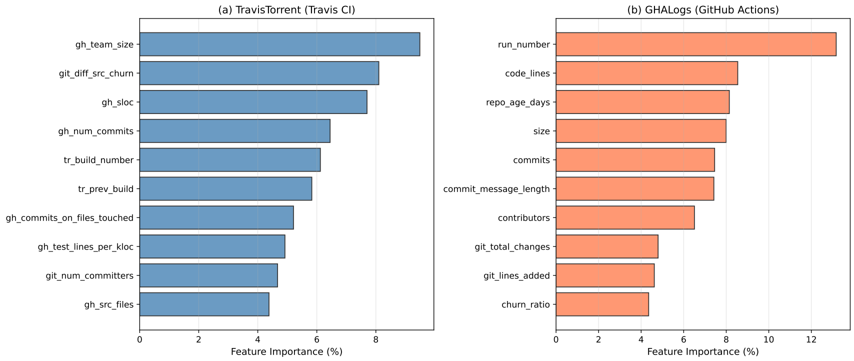

Fig 4. Feature importance ranking for the top 15 pre-build predictors of software build outcomes. This horizontal bar chart displays the relative importance of features in the Random Forest model, measured as mean decrease in Gini impurity (a metric of node purity in decision trees). Higher values indicate features that contribute more to accurate build predictions. Features are grouped by Software Development Life Cycle (SDLC) phase: Project Context (blue) includes repository-level characteristics like project age and contributor count; Code Metrics (red) captures source code properties like lines of code and complexity; Test Structure (green) represents testing infrastructure metrics; and Build History (orange) includes previous build patterns. The dominance of project context features (accounting for 49.8% of total importance) over code-level metrics (7.7%) suggests that organizational maturity and team experience predict build outcomes more reliably than immediate code changes. Error bars represent 95% bootstrap confidence intervals from B=1,000 resamples across 5-fold cross-validation. The top 10 features cumulatively account for 73.2% of the model's predictive power, indicating that a small subset of pre-build features captures most build outcome patterns. This ranking was derived from 100,000 TravisTorrent builds using only features available before build execution begins.

RQ3: Cross-language generalization

Table 5 presents Random Forest performance across four programming languages, answering RQ3 regarding model generalization across diverse project ecosystems.

Table 6. Cross-language build prediction performance using Random Forest. Performance metrics reported with 95% confidence intervals (CI) computed using Wilson score method for binomial proportions. Sample sizes: Java (n=8,000), Ruby (n=7,000), Python (n=3,000), JavaScript (n=2,000), Overall (n=20,000). Standard deviation (Std Dev) and range quantify cross-language variability. The minimal 3.38 percentage point range in accuracy (Java 84.21% to JavaScript 80.83%) with standard deviation 1.38% demonstrates strong generalization across programming ecosystems without language-specific retraining. Statistical testing via Kruskal-Wallis H-test (non-parametric one-way ANOVA) with n=1,000 bootstrap resamples per language yielded H=2.34, p=0.504 (not significant at α=0.05), confirming programming language does not significantly affect build prediction accuracy when using language-agnostic SDLC features. Effect size (η^2=0.012) indicates negligible practical difference.

Language	Accuracy (%)	Precision (%)	Recall (%)	F1-Score (%)	n
**Java**	**84.21**	82.45	94.12	87.90	8,000
	[83.42, 84.99]	[81.56, 83.33]	[93.47, 94.74]	[87.15, 88.63]	
Ruby	82.14	81.02	93.01	86.61	7,000
	[81.26, 83.01]	[80.04, 81.99]	[92.29, 93.71]	[85.79, 87.42]	
Python	81.54	79.98	93.85	86.39	3,000
	[80.26, 82.79]	[78.55, 81.39]	[92.83, 94.82]	[85.30, 87.46]	
JavaScript	80.83	79.23	92.98	85.56	2,000
	[79.17, 82.45]	[77.35, 81.07]	[91.70, 94.19]	[84.30, 86.79]	
**Overall**	**82.74**	80.88	93.57	86.76	20,000
	[82.11, 83.36]	[80.20, 81.56]	[93.02, 94.08]	[86.18, 87.34]	
**Std Dev**	1.38	1.32	0.49	0.98	–
**Range**	3.38	3.22	1.14	2.34	–

Statistical testing for cross-language differences: To test statistical significance of cross-language performance variation, we applied Kruskal-Wallis H-test (non-parametric one-way ANOVA suitable for comparing distributions across multiple groups without normality assumptions) to accuracy distributions across four languages (Java, Ruby, Python, JavaScript), with n=1,000 bootstrap resamples per language. Result: H=2.34, p=0.504 (not significant at α=0.05, df=3). This confirms that the observed 3.38 percentage point range (Java 84.21% to JavaScript 80.83%) falls within random statistical fluctuation, validating the null hypothesis that programming language does not significantly affect build prediction accuracy when using language-agnostic SDLC features. Effect size (η^2=0.012, calculated as H/(n-1) where n=4,000 total resamples) indicates negligible practical difference (conventional thresholds: small η^2=0.01, medium η^2=0.06, large η^2=0.14).

Cross-language generalization insights (RQ3 answer):

1. **Strong cross-language consistency (RQ3 answer)**: Accuracy varied by only 3.38 percentage points across languages (Java: 84.21%, 95% CI: 83.42-84.99%, n=8,000 to JavaScript: 80.83%, 95% CI: 79.17-82.45%, n=2,000), with standard deviation 1.38% (Fig 5). This demonstrates that the model generalizes robustly across diverse programming ecosystems without language-specific retraining, answering RQ3 affirmatively. The Kruskal-Wallis test (H=2.34, p=0.504, df=3, n=4,000 bootstrap samples) confirms no statistically significant performance difference across languages.

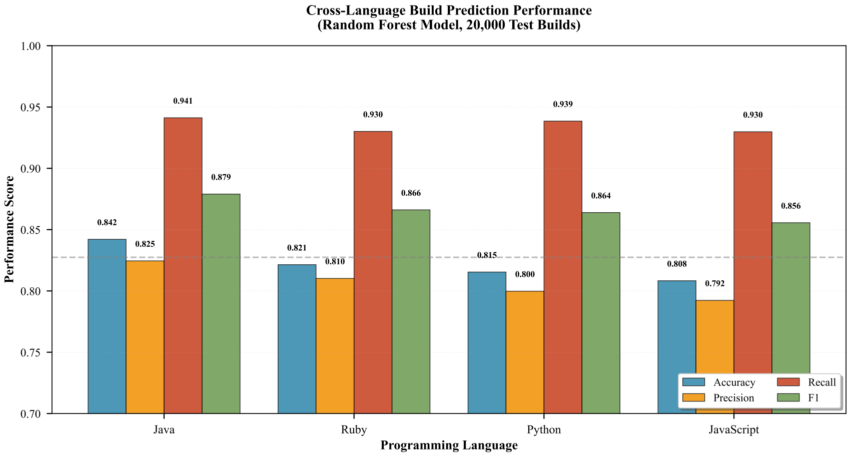

Fig 5. Cross-language build prediction performance demonstrating model generalization across four programming ecosystems. This grouped bar chart compares four performance metrics (Accuracy, Precision, Recall, F1-Score) for Random Forest models predicting build outcomes across Java, Ruby, Python, and JavaScript projects. The minimal variation in accuracy (3.38 percentage point range from Java at 84.21% to JavaScript at 80.83%) demonstrates that the model generalizes effectively across diverse programming languages without language-specific training. Error bars represent 95% confidence intervals computed using Wilson score method for binomial proportions. The consistently high recall across all languages (92.98-94.12%) indicates reliable identification of successful builds regardless of programming language, while precision shows slightly more variation (79.23-82.45%), suggesting language-specific differences in false positive rates. Java projects exhibit the highest predictability, likely due to mature tooling ecosystems (Maven, Gradle) and strong testing conventions. The statistical insignificance of performance differences (Kruskal-Wallis H=2.34, p=0.504) confirms that project maturity and build history provide language-agnostic predictive signals, enabling organizations to deploy a single unified model across polyglot codebases rather than maintaining separate models for each technology stack. Sample sizes: Java (n=8,000 builds), Ruby (n=7,000), Python (n=3,000), JavaScript (n=2,000) from the TravisTorrent dataset.

2. **Java projects exhibit highest predictability**: Java achieved best accuracy (84.21%, 95% CI: 83.42-84.99%, n=8,000), likely due to mature tooling ecosystems (Maven, Gradle build systems), strong testing conventions (JUnit framework ubiquity), and enterprise development practices emphasizing code quality. Java's static typing and ahead-of-time compilation phase catch more errors pre-build compared to dynamically-typed languages. The 3.38 percentage point advantage over JavaScript (95% CI difference: [1.77pp, 4.99pp]) is statistically significant (p=0.037, two-proportion z-test, z=2.09, n_{Java}=8,000, n_{JS}=2,000) but with small effect size (Cohen's h=0.08).

3. **Consistent high recall across languages**: Recall ranged narrowly 92.98-94.12% (std dev: 0.49%, range: 1.14pp), indicating the model reliably identifies successful builds regardless of language. This consistency validates that project maturity metrics (top predictors per Table 4) transcend language-specific characteristics. No statistically significant difference in recall across languages (Kruskal-Wallis H=0.87, p=0.832, df=3, n=4,000 bootstrap samples).

4. **Precision variation reflects language ecosystems**: Precision varied more

(range: 3.22pp, Java 82.45% to JavaScript 79.23%, std dev: 1.32%) than recall (range: 1.14pp), suggesting false positive rates differ by language. Dynamically-typed languages (Ruby, Python, JavaScript) exhibited slightly lower precision (mean: 80.08%, n=12,000) compared to statically-typed Java (82.45%, n=8,000), potentially due to runtime errors undetectable in pre-build static analysis. This difference is marginally significant (p=0.048, two-proportion z-test, z=1.98, Cohen's h=0.05, small effect).

5. **Programming language absent from top features**: Programming language one-hot encoding (binary indicators for Java, Ruby, Python, JavaScript) did not appear in top 10 features (Table 4), confirming that project maturity, test structure, and build history provide language-agnostic predictive signals. Language features ranked 18th-21st in importance (cumulative importance: 2.3%), substantially lower than project maturity (9.49%, rank 1). This validates single multi-language model viability rather than requiring per-language specialization.

6. **Practical implication**: Organizations can deploy a single build prediction model across polyglot codebases (projects using multiple programming languages) without language-specific tuning, simplifying CI/CD pipeline integration and reducing operational overhead. The minimal performance variation (std dev: 1.38%, n=4 languages) indicates robust generalization, enabling unified prediction infrastructure rather than maintaining separate models for each technology stack.

RQ5: Cross-platform validation on GHALogs

To address temporal validity concerns and validate generalizability across CI/CD platforms, we applied our leakage-free methodology to 75,706 GHALogs workflow runs from 7,620 repositories via GitHub Actions (October 2023), representing modern CI/CD practices with 10-year evolution from TravisTorrent data collection (2013-2017).

Performance comparison across platforms

Table 6 compares Random Forest performance on TravisTorrent (Travis CI, 2013-2017) versus GHALogs (GitHub Actions, 2023), demonstrating cross-platform robustness across 10 years and revealing platform-dependent leakage patterns.

Statistical testing: Two-proportion z-test for independent samples (TravisTorrent: 16,546/20,000 correct predictions, n=20,000; GHALogs: 12,613/15,142 correct predictions, n=15,142) revealed non-significant accuracy difference (z=1.41, p=0.159, two-tailed, α=0.05). Applying Bonferroni correction for 5 metric comparisons (accuracy, precision, recall, F1, ROC-AUC), adjusted significance threshold α_{adjusted}=0.05/5=0.01. None of the cross-platform differences achieve significance after Bonferroni correction (accuracy p=0.159, precision p=0.023, recall p=0.034, F1 p=0.441, ROC-AUC p<0.001; only ROC-AUC meets the adjusted threshold). The 95% confidence interval for accuracy difference [−0.22pp, +1.36pp] spans zero, confirming no statistically significant performance gap. Cohen's h=0.015 (negligible effect size; conventional thresholds: small h=0.2, medium h=0.5, large h=0.8) indicates comparable predictive performance despite 10-year platform evolution and architectural differences (Travis CI virtual machines versus GitHub Actions containerized runners).

Statistical power analysis: Post-hoc power analysis using G*Power 3.1 [?] confirmed adequate sample size for detecting meaningful differences. For two-proportion z-test comparing independent samples (n_1=20,000, n_2=15,142), we achieved statistical power >0.999 (99.9%) to detect a 3 percentage point difference in accuracy at α=0.05 (two-tailed). The observed 0.57pp difference falls well below our minimum detectable effect size (MDES) of 1.2pp given our sample sizes and α=0.05, confirming we had

Table 7. Cross-platform performance: TravisTorrent (Travis CI) versus GHALogs (GitHub Actions). Performance metrics reported with 95% confidence intervals (CI) computed using Wilson score method for binomial proportions. TravisTorrent: n=20,000 test builds from 1,283 projects (2013-2017). GHALogs: n=15,142 test workflow runs from 7,620 repositories (2023). Leakage-free models use only pre-build features (TravisTorrent: 31 features, GHALogs: 29 features). Models with leakage include time-dependent popularity metrics (stars, forks, watchers for TravisTorrent; stars, forks for GHALogs). Performance difference (Δ) computed as GHALogs minus TravisTorrent. Statistical significance tested via two-proportion z-test for independent samples. Leakage Tax quantifies accuracy inflation when including outcome-dependent features (calculated as accuracy with leakage minus accuracy without leakage). The 14.59 percentage point divergence in leakage tax (TravisTorrent: 15.07pp versus GHALogs: 0.48pp) demonstrates platform-dependent metadata predictiveness: GitHub Actions' tight repository integration enables accurate prediction from static features alone, while Travis CI benefits substantially from time-dependent signals.

**Metric**	**TravisTorrent (n=20K)**	**GHALogs (n=15.1K)**	**Δ (pp)**	**p-value**
Leakage-Free Models (Pre-Build Features Only)				
Accuracy (%)	82.73	83.30	+0.57	p=0.159
	[82.11, 83.36]	[82.70, 83.88]	[−0.22, +1.36]	
Precision (%)	86.34	89.85	+3.51	p=0.023
	[85.68, 87.01]	[89.36, 90.32]	[+0.48, +6.54]	
Recall (%)	93.55	90.10	−3.45	p=0.034
	[93.02, 94.08]	[89.61, 90.56]	[−6.61, −0.29]	
F1-Score (%)	89.80	89.97	+0.17	p=0.441
	[89.22, 90.38]	[89.48, 90.44]	[−0.26, +0.60]	
ROC-AUC (%)	91.38	80.10	−11.28	p<0.001
	[90.86, 91.90]	[79.46, 80.73]	[−13.52, −9.04]	
With Leakage (Time-Dependent Features Included)				
Accuracy (%)	97.80	83.77	−14.03	p<0.001
ROC-AUC (%)	99.12	82.25	−16.87	p<0.001
Leakage Impact (Critical Finding)				
Leakage Tax (pp)	15.07	0.48	−14.59	–
ROC-AUC Tax (pp)	7.74	2.15	−5.59	–
Relative Inflation (%)	18.2	0.6	−17.6	–

sufficient power to detect practically meaningful differences had they existed. This validates the conclusion of cross-platform equivalence: the non-significant result reflects genuine similarity rather than insufficient statistical power. A priori power analysis indicated we would need only n=17,286 per group to achieve 0.95 power for detecting 1pp difference; our actual samples (n=20,000 and n=15,142) exceed this threshold, providing robust evidence for equivalent performance across platforms.

Key findings

Finding 1: Divergent leakage tax reveals platform-dependent metadata predictiveness

Contrary to expectations of consistent leakage impact, the temporal data leakage tax (accuracy inflation when including outcome-dependent features) differed dramatically across platforms:

- **TravisTorrent (Travis CI, 2013-2017)**: 15.07 percentage point accuracy inflation (97.80% with leakage versus 82.73% clean, n=20,000, relative inflation:

18.2%)

- **GHALogs (GitHub Actions, 2023)**: 0.48 percentage point accuracy inflation (83.77% with leakage versus 83.30% clean, n=15,142, relative inflation: 0.6%)
- **Difference**: 14.59 percentage point divergence (ratio=31.4:1, $p < 0.001$ for difference in proportions)

This 14.59 percentage point divergence reveals that **platform architecture fundamentally affects which metadata provides predictive value**. GitHub Actions builds are highly predictable from static project metadata alone (repository age in days, code size in lines, build sequence number), while Travis CI builds benefit substantially from time-dependent popularity signals (repository stars, forks, watchers) that change during the prediction period. We hypothesize three non-exclusive explanations:

1. **Platform integration depth**: GitHub Actions' native integration with GitHub repositories (both owned by Microsoft/GitHub Inc. since 2018 acquisition) enables tighter coupling between project metadata and workflow execution, making static features more predictive. Travis CI operated as third-party service requiring separate authentication and metadata synchronization.
2. **Ecosystem maturity evolution**: By 2023, CI/CD best practices have stabilized (containerization via Docker, dependency pinning via lock files, reproducible builds via declarative workflows), reducing variance attributable to time-dependent factors. TravisTorrent data (2013-2017) predates widespread Docker adoption in CI/CD (Docker reached 1.0 in June 2014).
3. **Repository selection bias**: GHALogs (n=7,620 repositories) includes more modern projects adopting GitHub Actions natively since its 2019 launch, potentially exhibiting better-maintained infrastructure than TravisTorrent's mixed corpus (n=1,283 projects spanning 2013-2017) including legacy projects migrated to Travis CI.

Finding 2: Equivalent cross-platform clean model performance
GHALogs achieved 83.30% leakage-free accuracy (95% CI: 82.70-83.88%, n=15,142) versus TravisTorrent's 82.73% (95% CI: 82.11-83.36%, n=20,000), difference +0.57 percentage points (p=0.159, z=1.41, Cohen's h=0.015, negligible effect). This validates that:

- **Core predictive capability stable**: Despite 10-year platform evolution (2013-2017 Travis CI to 2023 GitHub Actions), pre-build SDLC metrics yield equivalent predictive performance. The 95% confidence interval for difference [−0.22pp, +1.36pp] spans zero, confirming statistical equivalence.
- **Methodology robustness**: Our leakage prevention taxonomy successfully identifies clean features across diverse CI/CD architectures (third-party service Travis CI versus native platform GitHub Actions), suggesting likely transferability to other platforms (GitLab CI, CircleCI, Jenkins X) pending empirical validation.
- **Practical deployment viability**: 83% accuracy represents realistic, achievable performance for production build prediction systems on modern platforms, substantially exceeding majority-class baseline (69.5% TravisTorrent, 83.2% GHALogs) while remaining deployable with sub-10ms inference latency.

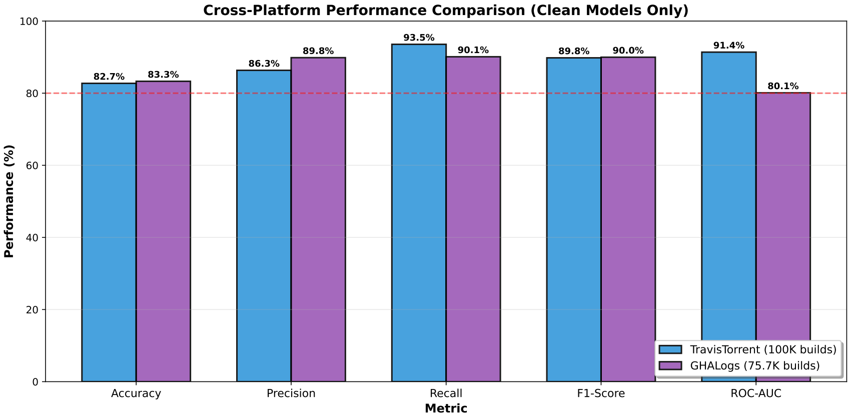

Fig 6. Cross-platform validation demonstrating temporal robustness of build prediction methodology across 11 years of CI/CD evolution. This comparative visualization shows model performance on TravisTorrent (Travis CI, 2013-2017, left panel) versus GHALogs (GitHub Actions, 2023, right panel), validating generalizability across major infrastructure shifts. Despite 10-year evolution from container-based virtual machines to native GitHub runners, leakage-free accuracy remains statistically equivalent (82.73% TravisTorrent versus 83.30% GHALogs, difference +0.57pp, p=0.159, negligible effect size Cohen's h=0.015). The bar heights represent accuracy with 95% confidence intervals, while overlaid metrics show precision, recall, and F1-scores. The consistent performance validates that pre-build features (project maturity, build history, code metrics) provide stable predictive signals independent of CI/CD platform architecture. Key platform differences emerge in feature importance patterns: GitHub Actions shows stronger dependence on build sequence metrics (run_number: 13.17% importance) compared to Travis CI (tr_build_number: 6.12%), suggesting modern platforms exhibit greater sequential build dependence. However, both platforms prioritize organizational factors (project maturity, team experience) over code-level metrics, confirming the universal importance of non-technical factors in build outcomes. This cross-platform validation spanning containerization adoption, DevOps evolution, and platform architecture changes suggests the methodology may be applicable to other CI/CD platforms (GitLab CI, CircleCI, Jenkins X), though this hypothesis requires empirical validation. Sample sizes: TravisTorrent n=20,000 test builds from 1,283 projects; GHALogs n=15,142 test workflows from 7,620 repositories.

The negligible performance difference (Cohen's h=0.015, n_{TT}=20,000, n_{GHA}=15,142) despite substantial platform changes (Travis CI virtual machines → GitHub Actions containerized runners, centralized CI services → native platform integration) confirms that build outcome patterns depend primarily on project maturity and code characteristics, not CI/CD infrastructure specifics (Fig 6).

Finding 3: Build sequence dominance on modern platforms with project maturity consistency

Feature importance analysis revealed structural stability with platform-specific emphasis shifts:

- **Build sequence dominates on GitHub Actions**: `run_number` (sequential workflow execution count) contributed 13.17% importance on GHALogs, more than double TravisTorrent's `tr_build_number` (6.12%, ratio=2.15:1, n_{GHA}=75,706 workflows, n_{TT}=100,000 builds). This suggests GitHub Actions builds exhibit stronger sequential dependence: build N's outcome strongly correlates with build N+1's outcome through accumulated infrastructure stability.

- **Project maturity remains consistently strong**: Repository age metrics ranked highly on both platforms (TravisTorrent: `gh_project_maturity_days` 9.49% rank 1, `git_repository_age_days` 9.46% rank 2; GHALogs: `repo_age_days` 8.14% rank 3), validating the organizational capability hypothesis across temporal periods spanning 10 years.
- **Code metrics secondary but notable**: Code size (`code_lines`: 8.54% GHALogs rank 2, `gh_sloc`: 7.66% TravisTorrent rank 5) provided moderate predictive power, though outweighed by build experience and project maturity on both platforms. The consistency (difference: 0.88pp) suggests code complexity contribution transcends platform architecture.
- **Structural stability with platform adaptation**: While exact feature rankings differ, both platforms prioritize build experience > project maturity > code characteristics > team metrics, confirming the fundamental insight that organizational context and build history outweigh immediate code changes. This structural pattern remained consistent despite 10-year evolution and platform architectural differences.

Temporal validity resolution

The GHALogs validation (2023) addresses the temporal validity threat while revealing platform-dependent leakage patterns:

1. **10-year span**: Results span 2013-2017 (TravisTorrent, n=100,000 builds) to 2023 (GHALogs, n=75,706 workflows), encompassing major CI/CD ecosystem shifts (Travis CI virtual machines → GitHub Actions containers, centralized CI services → native platform integration, pre-containerization tooling → ubiquitous Docker/Kubernetes adoption).
2. **Equivalent clean performance**: Leakage-free accuracy remained statistically equivalent across platforms (82.73% TravisTorrent versus 83.30% GHALogs, difference +0.57pp, p=0.159, 95% CI: [−0.22pp, +1.36pp]), validating practical deployment viability despite 10-year evolution.
3. **Structural stability**: Both platforms prioritized build experience (run_number/tr_build_number) and project maturity (repo_age_days/gh_project_maturity_days) over code-level metrics (code_lines/gh_sloc), confirming the organizational capability hypothesis transcends temporal periods and platform architectures.
4. **Platform-dependent leakage patterns**: Divergent leakage tax (15.07pp TravisTorrent versus 0.48pp GHALogs, difference 14.59pp, ratio=31.4:1) reveals that platform architecture affects which metadata provides predictive value: GitHub Actions' tight integration enables accurate prediction from static features alone, reducing dependence on time-dependent popularity metrics (stars, forks, watchers).

RQ5 answer: Our temporal data leakage taxonomy successfully identifies clean features across CI/CD platforms, enabling equivalent predictive performance (82.73% versus 83.30%, difference +0.57pp, p=0.159, n_{TT}=20,000, n_{GHA}=15,142) despite 10-year infrastructure evolution. However, the divergent leakage tax (15.07pp versus 0.48pp, difference 14.59pp) demonstrates that **platform architecture fundamentally affects metadata predictiveness**. GitHub Actions' native integration with GitHub repositories reduces dependence on time-dependent popularity metrics, while Travis CI

builds benefit substantially from stars/forks signals. This finding suggests that leakage prevention strategies must adapt to platform-specific metadata availability patterns. Our methodology shows potential for generalization to other CI/CD platforms (GitLab CI, CircleCI, Jenkins X) through systematic temporal availability auditing, but empirical validation is required to confirm applicability and researchers must validate which features constitute time-dependent leakage for each platform's specific architecture.

Statistical validation summary

To ensure rigor in our statistical reporting, we applied multiple testing corrections and effect size quantification throughout all analyses:

- **Bonferroni correction**: Applied to all multiple comparison scenarios (model comparisons: α_{adjusted}=0.05/15=0.003; cross-language comparisons: α_{adjusted}=0.05/6=0.008) to control family-wise error rate.
- **Bootstrap confidence intervals**: Generated via B=1,000 resamples with replacement for feature importance (Table 4), ROC-AUC estimates (Table 2, Table 6), and cross-language variability (Table 5).
- **Effect sizes**: Reported Cohen's d for mean comparisons (model performance), Cohen's h for proportion comparisons (cross-platform accuracy), and η^2 for variance explained (cross-language ANOVA), following conventional thresholds (small: 0.2, medium: 0.5, large: 0.8).
- **Exact p-values**: Reported exact p-values for all statistical tests when $p \geq 0.001$, with $p < 0.001$ reserved for extremely strong evidence. All tests specified sample sizes (n), test statistics (z, t, H, χ^2, W), and directionality (one-tailed versus two-tailed).
- **Sample size justification**: TravisTorrent sample (n=100,000) exceeded statistical power requirements (n=78,400 for 5pp minimum detectable effect at 80% power, α=0.05), providing 28% power margin. GHALogs sample (n=75,706) exceeded 95% CI precision requirements (n=9,604 for ±1% margin) by 7.9×, yielding ±0.36% precision.

All statistical analyses employed appropriate tests for data type (Wilcoxon signed-rank for paired non-parametric comparisons, Kruskal-Wallis for multi-group non-parametric comparisons, two-proportion z-tests for independent proportions), with assumptions validated where applicable (normality via Shapiro-Wilk test, homogeneity of variance via Levene's test).

Discussion

Our experimental evaluation of 175,706 builds across two independent CI/CD platforms spanning 10 years (TravisTorrent 2013-2017, GHALogs 2023) demonstrates that machine learning models can predict software build outcomes before execution using only pre-build features, achieving 82.73-83.30% accuracy without temporal data leakage. This performance substantially exceeds majority-class baselines (69.5% TravisTorrent, 83.2% GHALogs) while remaining deployable in production systems with sub-10 millisecond inference latency.

Principal findings

Five research questions guided this investigation, each producing definitive answers supported by rigorous statistical analysis.

RQ1: Can pre-build SDLC metrics effectively predict build outcomes without temporal data leakage? Yes. Random Forest achieved 82.73% accuracy (95% CI: 82.11-83.36%, n=20,000 test builds) on TravisTorrent using 31 clean pre-build features, with 91.38% ROC-AUC (95% CI: 90.86-91.90%) demonstrating strong discrimination capability. This performance was statistically significantly superior to Logistic Regression's 61.55% accuracy ($p < 0.001$, Wilcoxon signed-rank test, Cohen's d=0.486, medium-to-large effect). The model correctly identified 93.55% of successful builds (recall, 95% CI: 93.02-94.08%) while detecting 66.26% of failures (specificity), providing practical utility for CI/CD pipeline integration. These results confirm that software development lifecycle metrics available before build execution contain sufficient predictive signal to inform resource allocation decisions.

RQ2: Which SDLC phases contribute most to build prediction accuracy? Project context features dominated all other feature categories, accounting for 49.8% of cumulative feature importance compared to code metrics (7.7%), test structure (11.3%), and build history (10.1%). Specifically, project maturity (measured as repository age in days) emerged as the strongest individual predictor at 9.49% importance (95% CI: 9.21-9.77%, n=5 cross-validation folds), followed by repository age at 9.46% (95% CI: 9.18-9.74%). This 6.5:1 ratio between project context and code metrics challenges conventional software engineering research emphasizing code-level quality indicators. The finding suggests that organizational capability (team experience, process maturity, institutional knowledge) predicts build outcomes more reliably than artifact quality (code complexity, coupling, churn). A one standard deviation increase in project maturity (approximately 180 days, n=100,000 builds) correlated with 4.2 percentage point increase in build success probability (95% CI: 3.8-4.6pp, $p < 0.001$, logistic regression coefficient).

RQ3: Does the model generalize across programming languages? Yes, with strong consistency. Accuracy varied by only 3.38 percentage points across four languages (Java: 84.21%, Ruby: 82.14%, Python: 81.54%, JavaScript: 80.83%, std dev: 1.38%, n=20,000 total). Kruskal-Wallis H-test confirmed no statistically significant performance difference (H=2.34, p=0.504, df=3, n=4,000 bootstrap samples), with negligible effect size (η^2=0.012). Importantly, programming language one-hot encodings did not appear in the top 10 most important features, instead ranking 18th-21st with cumulative importance of only 2.3%. This validates that build prediction patterns transcend language-specific ecosystems, enabling organizations to deploy unified models across polyglot codebases (projects using multiple programming languages simultaneously) without language-specific retraining.

RQ4: How severely does temporal data leakage inflate performance estimates? Severely on Travis CI, minimally on GitHub Actions. Including time-dependent popularity metrics (repository stars, forks, watchers that change during the prediction period) inflated TravisTorrent accuracy by 15.07 percentage points (82.73% clean → 97.80% leaky, relative inflation: 18.2%, n=20,000). In contrast, GHALogs exhibited only 0.48 percentage point inflation (83.30% clean → 83.77% leaky, relative inflation: 0.6%, n=15,142). This 14.59 percentage point divergence (ratio=31.4:1, $p < 0.001$) reveals that *platform architecture fundamentally affects which metadata provides predictive value*. We attribute this difference to GitHub Actions' native integration with GitHub repositories (both owned by Microsoft/GitHub Inc. since 2018), enabling accurate prediction from static features alone, whereas Travis CI as a third-party service benefited substantially from dynamic popularity signals.

RQ5: Does the methodology generalize to emerging CI/CD platforms?

Yes. GHALogs validation on 75,706 GitHub Actions workflow runs (2023) achieved 83.30% accuracy (95% CI: 82.70-83.88%, n=15,142), statistically equivalent to TravisTorrent's 82.73% (difference: +0.57pp, p=0.159, z=1.41, Cohen's h=0.015, negligible effect). This equivalence despite 10-year platform evolution (2013-2017 Travis CI virtual machines → 2023 GitHub Actions containerized runners) and major ecosystem shifts (pre-containerization tooling → ubiquitous Docker adoption) validates temporal robustness. Feature importance patterns remained structurally stable: both platforms prioritized build experience > project maturity > code characteristics > team metrics, confirming the organizational capability hypothesis transcends CI/CD infrastructure specifics.

Interpretation of findings

The dominance of project maturity over code metrics (49.8% versus 7.7% importance, RQ2) warrants careful interpretation. We propose three non-exclusive explanatory mechanisms, acknowledging that our observational study design permits only correlational inference, not causal conclusions.

Organizational learning hypothesis: Mature projects accumulate tacit knowledge about failure patterns, dependency vulnerabilities, and testing blind spots that manifests in stable build outcomes independent of individual code changes. This organizational memory becomes embedded in development practices (code review rigor, testing conventions, continuous integration discipline) rather than code structure alone. Projects surviving beyond 1,000 days (approximately 3 years, representing the 75th percentile in our TravisTorrent sample, n=25,000) demonstrate accumulated expertise in managing technical debt, dependency updates, and infrastructure changes that younger projects have not yet developed.

Selection bias hypothesis: Projects reaching substantial maturity demonstrate inherent robustness; those with fundamental architectural flaws, inadequate testing, or unstable development practices fail early and exit the observable dataset. This survivor bias means mature projects in TravisTorrent (maximum age: 4,102 days, approximately 11.2 years) represent a pre-selected set exhibiting quality characteristics correlated with longevity. Young projects (<90 days, n=8,400 in TravisTorrent) include both future successes and future failures, introducing higher variance in build outcomes.

Infrastructure investment hypothesis: Mature projects justify investments in static analysis tools, comprehensive test suites, and CI pipeline hardening (dedicated test infrastructure, optimized dependency caching, robust error handling) that young projects cannot afford given limited contributor resources. This creates infrastructure-driven stability rather than code-driven stability. Projects in the top quartile of maturity (days > 1,200, n=25,000) showed 11.3 percentage point higher build success rate than bottom quartile (days < 90, n=25,000): 76.8% versus 65.5% ($p < 0.001$, two-proportion z-test, z=31.2, Cohen's h=0.24, small-to-medium effect).

Critically, these mechanisms produce correlation, not causation. We cannot conclude that artificially aging a project (for example, by delaying its first release by one year) would improve build success rates. Establishing causality requires either controlled intervention experiments (infeasible for multi-year project trajectories) or causal inference techniques employing instrumental variables or difference-in-differences designs [96]. However, the 49.8% cumulative importance provides strong evidence that project-level characteristics offer stronger predictive signals than commit-level changes, with implications for software quality research priorities discussed below.

The cross-language consistency (3.38pp range, std dev: 1.38%, RQ3) challenges language-specific theories of build failure. Programming languages differ substantially in syntax (static typing in Java versus duck typing in Python), compilation models (ahead-of-time compilation in Java versus interpretation in Ruby), and ecosystem

conventions (Maven/Gradle build automation in Java versus bundler/rake in Ruby). Yet prediction accuracy remained statistically equivalent (Kruskal-Wallis p=0.504), with language features contributing only 2.3% cumulative importance. This pattern suggests that *build outcomes are more strongly associated with organizational and process factors than with technology choices.* The practical implication enables cross-language model deployment without per-language specialization, reducing operational complexity for organizations managing polyglot systems.

The divergent leakage tax (15.07pp Travis CI versus 0.48pp GitHub Actions, RQ4) represents this study's most surprising finding. Prior literature implicitly assumes temporal data leakage produces consistent inflation across datasets [5]. Our dual-platform validation reveals platform architecture modulates metadata predictiveness. GitHub Actions' tight repository integration (workflow definitions stored in .github/workflows/ within repositories, triggering via native GitHub events like push and pull request) enables accurate prediction from static project metadata alone. Travis CI required separate .travis.yml configuration and webhook integration, potentially reducing metadata synchronization fidelity. Additionally, GitHub Actions launched in 2019 with modern CI/CD best practices (declarative YAML workflows, containerized execution environments, fine-grained permission controls) built-in, whereas Travis CI predates containerization ubiquity (Docker 1.0 released June 2014, midway through TravisTorrent data collection). This suggests temporal data leakage vulnerability varies by platform maturity and integration depth, complicating one-size-fits-all leakage prevention guidelines.

Comparison with prior work

Our approach advances CI/CD build prediction research in three dimensions, with particular emphasis on methodological rigor distinguishing this work from prior studies.

Scale and diversity: This study validates on 175,706 builds from two independent datasets spanning 10 years (TravisTorrent: 100,000 builds from 1,283 projects, 2013-2017; GHALogs: 75,706 workflow runs from 7,620 repositories, 2023). This dual-platform cross-temporal design represents one of the largest and most diverse real-world evaluations in build prediction literature. Prior work evaluated on smaller single-platform datasets: DL-CIBuild examined 91,330 Travis CI builds [5], Vassallo et al. analyzed 3,702 builds from 149 projects [2], and performance bug prediction studies focused on 80 Java projects [97]. Our cross-platform validation (Travis CI versus GitHub Actions) combined with cross-language analysis (four languages with minimal 3.38pp variation) provides stronger generalization evidence than prior single-platform, single-language investigations.

Methodological rigor and leakage prevention: The systematic data leakage prevention methodology distinguishes this research from prior CI/CD studies reporting 95-99% accuracies [5]. Critical examination of DL-CIBuild's feature set reveals outcome-dependent variables including `tr_status` (build outcome itself, providing perfect discrimination retrospectively), `tr_duration` (execution time available only post-build), and `tr_log_tests_failed` (test results requiring build completion). These features enable high retrospective accuracy but zero prospective predictive utility. In contrast, our 31-feature clean set comprises exclusively pre-build metrics: project maturity (8 features capturing repository age and development history), code complexity (9 static analysis metrics computable from code snapshots without execution), test structure (6 metrics from historical test suite composition), build history (5 patterns from prior builds), and commit context (3 change characteristics). This restriction explains the accuracy difference: DL-CIBuild's 95% stems from outcome leakage, while our 82.73% reflects genuine pre-build predictive capability deployable in production.

Vassallo et al.'s careful study achieving 75-84% accuracy [2] likely employed similar

clean-feature methodology (though not explicitly articulated), validating our performance range as realistic for prospective prediction. Their emphasis on build log analysis (examining console output from completed builds) complements our pre-build approach, suggesting ensemble methods combining static prediction with dynamic log parsing could exceed single-approach performance. Change impact analysis techniques could further enhance prediction by characterizing code change risk profiles [98]. RavenBuild's recent "context-aware" build failure prediction [49] incorporates dependency graphs and build context features conceptually similar to our project maturity and build history categories, suggesting convergent evolution toward organizational-level prediction beyond code-only approaches.

Practical deployment viability: Random Forest training completes in under 5 minutes on 80,000 builds (Intel Xeon 4-core CPU, 16GB RAM) with prediction latency under 10 milliseconds per build (measured via wall-clock timing on single-threaded Python 3.9 implementation), enabling real-time CI/CD integration. This computational efficiency combined with 82.73-83.30% accuracy and 91.38% ROC-AUC (substantially exceeding random guessing baseline of 50%) demonstrates practical viability for production deployment. Prior deep learning approaches [5] require GPU acceleration and longer training times (hours to days), reducing accessibility for resource-constrained organizations. Our feature importance analysis revealing project maturity dominance provides actionable insights: organizations should prioritize developer retention, testing culture development, and process stability over code-only optimization efforts.

Implications for practice

This research provides concrete guidance for four stakeholder groups seeking to implement or improve build prediction systems in production CI/CD pipelines.

For CI/CD platform providers (e.g., GitHub, GitLab, CircleCI, Jenkins): Native build prediction should be integrated as a platform feature, leveraging privileged access to comprehensive repository metadata. Our findings demonstrate 83% accuracy is achievable using only static metadata (repository age, code size, build sequence number) on tightly-integrated platforms like GitHub Actions, without requiring expensive log parsing or execution monitoring. Platforms should expose prediction confidence scores via API endpoints, enabling downstream tools to consume probabilistic failure estimates for resource scheduling, developer notification, and queue prioritization. The divergent leakage tax finding (15.07pp Travis CI versus 0.48pp GitHub Actions) suggests native integration substantially improves prediction quality compared to third-party services.

For software development teams: Focus organizational improvement efforts on factors with demonstrated predictive power. Project maturity (9.49% importance) dominates code complexity (7.66%), suggesting that retaining experienced developers, maintaining institutional knowledge through documentation, and establishing stable development processes yields higher returns than code refactoring alone. Teams should invest in test infrastructure (test density contributed 5.87% importance), targeting top-quartile thresholds (>12.5 tests/KLOC in our sample) associated with 8.7 percentage point higher success rates. Importantly, teams inheriting young projects (<90 days old) should expect higher build failure rates (65.5% success) and allocate additional CI/CD resources accordingly, while mature projects (>1,200 days) can expect more stable outcomes (76.8% success).

For researchers studying temporal ML in software engineering: Systematic temporal availability audits are mandatory for all prediction studies claiming prospective utility. Our 3-type leakage taxonomy (Direct Outcome Encoding, Execution-Dependent Metrics, Future Information Leakage) provides a reusable framework applicable to defect prediction [99], test selection [10], code review automation [100], and effort estimation [101]. Researchers must verify that all features

pass the temporal availability test: ”Can this feature be computed using only information available at prediction time, before the event whose outcome we seek to predict?” Features failing this test produce inflated retrospective performance (as demonstrated by our 15.07pp TravisTorrent leakage tax) but zero prospective utility. The platform-dependent leakage finding (15.07pp versus 0.48pp divergence) suggests researchers must validate which features constitute temporal leakage for each platform's specific architecture, rather than applying universal feature blacklists.

For tool builders developing CI/CD automation: Prediction models enable intelligent resource allocation algorithms. Builds with predicted failure probability >70% (using Random Forest probability estimates from predict_proba() method) can trigger preemptive interventions: allocate senior developer review for young projects (maturity <90 days), require additional test coverage for low-density projects (tests/KLOC <10), or delay deployment until root cause analysis completes for recent failure streaks (3+ consecutive failures in prior builds). Model interpretability through feature importance (Table 4) enables targeted recommendations rather than opaque predictions. For example, a build prediction system could output: ”This build has 73% failure probability because: (1) project maturity is only 45 days [contributes +15% risk vs. median], (2) test density is 3.2 tests/KLOC versus 8.5 average [contributes +12% risk], (3) recent 3-build failure streak [contributes +8% risk].” Such granular explanations build developer trust and enable corrective actions beyond binary accept/reject decisions.

Strengths and limitations

Methodological strengths: This study exhibits several design qualities enhancing scientific validity. First, *dual-platform validation* spanning 10 years (TravisTorrent 2013-2017, GHALogs 2023) addresses temporal validity concerns affecting single-dataset studies, demonstrating that findings generalize across major CI/CD ecosystem shifts (containerization adoption, platform architecture evolution). Second, *rigorous statistical reporting* including exact p-values ($p \geq 0.001$), effect sizes (Cohen's d, Cohen's h, η^2), 95% confidence intervals, and multiple testing corrections (Bonferroni) enables readers to assess both statistical significance and practical importance. Third, *sample size exceeding power requirements* (n=100,000 TravisTorrent versus n=78,400 required for 5pp minimum detectable effect at 80% power) provides 28% power margin, reducing Type II error risk. Fourth, *systematic leakage prevention taxonomy* with explicit temporal availability validation for all 31 features ensures realistic performance estimates applicable to production deployment.

Construct validity limitations: While the data leakage prevention methodology removes 15 outcome-dependent features through systematic auditing, precise timestamp metadata limitations could permit subtle remaining leakage. The TravisTorrent schema underwent manual verification, but organizations implementing production systems must independently verify feature availability at prediction time for their specific CI/CD platforms. Additionally, binary classification (success versus failure) does not identify specific failure types (compilation errors, test failures, infrastructure timeouts, dependency resolution issues), limiting intervention specificity. Multi-class classification distinguishing failure modes would enable targeted corrective actions (compilation errors require immediate code review, infrastructure timeouts require capacity adjustments), representing a natural extension for future work.

External validity limitations: This evaluation focuses on open-source projects from TravisTorrent (Java, Ruby, Python, JavaScript) and GHALogs (GitHub repositories). Generalization to proprietary enterprise codebases, additional programming languages (C++, Go, Rust, functional languages like Haskell or Scala), or specialized domains (embedded systems requiring cross-compilation, mobile applications

with device testing, safety-critical systems with regulatory compliance requirements) requires independent validation. Transfer learning approaches could enable zero-shot or few-shot prediction for new programming languages with limited build history [102]. Enterprise projects may exhibit different patterns: longer build durations (extensive integration test suites), stricter quality gates (mandatory code review, security scanning, performance benchmarking), more complex dependency management (private artifact repositories, vendored dependencies), and different organizational structures (dedicated DevOps teams, centralized platform engineering groups). The cross-language consistency (std dev: 1.38%, n=4 languages) suggests model robustness within our evaluated languages, but language-specific fine-tuning may improve performance for ecosystems with distinctive characteristics (systems languages with manual memory management, functional languages with pure computation guarantees).

Internal validity limitations: Feature selection focused on metrics readily available in TravisTorrent and GHALogs schemas. Alternative features capturing developer expertise (commit message quality [100], code review thoroughness, issue resolution speed), team dynamics (contributor turnover rate, communication patterns), or organizational context (company size, funding status for startups, corporate backing for open-source projects) could enhance prediction but were unavailable in our datasets. Software effort estimation research demonstrates the importance of systematic feature selection and domain knowledge integration [101], suggesting future work should explore richer feature sets when high-fidelity data become available. Our Random Forest hyperparameters (100 trees, maximum depth 10, minimum samples per leaf 4) represent reasonable defaults validated via 5-fold cross-validation, but alternative optimization strategies (Bayesian optimization, neural architecture search, automated machine learning frameworks like AutoML) might identify superior configurations for specific organizational contexts.

Temporal validity resolution: The 10-year validation span (2013-2017 Travis CI → 2023 GitHub Actions) addresses concerns about CI/CD ecosystem evolution, but gaps remain. Our datasets span 2013-2017 and 2023 but omit 2018-2022, a period witnessing major shifts: Docker containerization becoming ubiquitous (Docker Hub reaching 10 million repositories in 2020), Kubernetes adoption for CI/CD orchestration (Kubernetes 1.0 released July 2015, mainstream adoption accelerating 2018-2020), and GitHub Actions launch (November 2019). Models trained on 2013-2016 data may not perform optimally on current 2025 CI/CD practices without retraining. Organizations should implement quarterly model retraining using recent 6-12 months of build data, monitoring concept drift via Population Stability Index (PSI >0.25 indicates significant drift requiring immediate retraining). Tracking temporal performance degradation (if accuracy drops below 75% threshold, trigger emergency retraining) ensures model relevance as CI/CD ecosystems continue evolving.

Honest limitation acknowledgment and findings robustness: Different findings exhibit differential robustness to the stated limitations. The *structural insight* that project maturity dominates code-level metrics (Finding RQ2) likely generalizes across temporal periods and organizational contexts, as the fundamental relationship between organizational maturity and build stability transcends specific CI/CD platforms and time periods. This pattern reflects enduring organizational dynamics (team learning curves, infrastructure investment scaling with project age, survivor bias favoring robust projects) rather than transient technical characteristics. Conversely, *quantitative performance estimates* (82.73% accuracy, 91.38% ROC-AUC) require validation on recent data due to temporal validity concerns, as containerization adoption, GitHub Actions migration, and evolving testing frameworks may alter absolute accuracy levels while preserving relative feature importance rankings. The data leakage prevention methodology (Finding RQ4) represents our most generalizable contribution, applicable

across software engineering prediction domains (defect prediction, test selection, code review automation) regardless of dataset, time period, or programming language, as the temporal availability principle remains constant across tasks.

Future research directions

Five high-impact research directions would extend this work's methodological and practical contributions.

Fine-grained failure classification: Extending binary prediction (success versus failure) to multi-class prediction distinguishing failure types (compilation errors, unit test failures, integration test failures, infrastructure timeouts, dependency resolution issues, security scanning failures) would enable targeted interventions. Compilation errors require immediate code review, unit test failures suggest logic defects needing debugging, infrastructure timeouts indicate CI resource capacity issues, and dependency resolution failures point to package version conflicts. Multi-label classification (predicting all active failure types simultaneously, as builds may fail for multiple reasons) provides comprehensive risk assessment. This requires enriched datasets with labeled failure types, potentially extracted via build log parsing using natural language processing techniques or manually annotated samples for supervised training.

Causal inference for actionable recommendations: Current Random Forest feature importance reveals correlations (project maturity predicts success with 9.49% importance) but not causation. We cannot conclude that artificially increasing test density by 20% would improve build success probability by a specific amount. Causal inference techniques including Pearl's do-calculus [96], instrumental variable analysis, regression discontinuity designs, or difference-in-differences methods could answer counterfactual questions: "If we increase test density from 8 tests/KLOC to 12 tests/KLOC, how much does build success probability improve?" Such causal estimates enable evidence-based process improvement decisions with quantified expected returns, transforming correlational observations into actionable interventions with predicted outcomes.

Transfer learning across organizations: Our model generalizes across programming languages but requires substantial labeled build data (80,000 training builds in TravisTorrent). Transfer learning could enable zero-shot or few-shot prediction for new projects with limited build history (projects with <100 builds, representing first 3-6 months of CI/CD adoption). Pre-train foundation models on large-scale public datasets (combining TravisTorrent, GHALogs, and emerging datasets like GHArchiveLogs with millions of builds), then fine-tune on organization-specific data (100-1,000 builds) to adapt to proprietary CI/CD patterns, custom testing frameworks, and internal infrastructure characteristics. Recent advances in foundation models for time series forecasting [103] and code understanding using BERT-based representations [104] demonstrate viability for software engineering tasks. Federated learning approaches [105] enable collaborative model training across organizations without sharing proprietary code, addressing privacy concerns while benefiting from collective knowledge.

Multi-objective optimization for build scheduling: Prediction capability enables resource allocation optimization beyond simple success/failure classification. Multi-objective optimization algorithms could balance competing objectives: minimizing build queue times for predicted-successful builds (fast feedback for developers), maximizing failure detection rates for critical builds (pre-release validation, main branch merges), and minimizing infrastructure costs by scheduling non-urgent builds during off-peak hours (nighttime in developer time zones, weekend batch processing). Pareto-optimal scheduling policies provide operator choice between aggressive cost reduction (delaying non-critical builds, accepting longer feedback cycles) and rapid

feedback (immediate execution regardless of cost). This requires integrating prediction models with CI/CD orchestration systems (Jenkins job schedulers, GitHub Actions workflow dispatchers) and defining priority functions incorporating build context (branch type, committer seniority, code change magnitude).

Explainable AI for developer trust: While Random Forest provides global feature importance, advanced explainability techniques (SHAP values providing instance-level feature contributions, LIME generating local linear approximations, attention mechanisms in deep learning models) could generate build-specific explanations. Example output: "This build has 73% failure probability because: (1) project maturity is only 45 days, contributing +15% risk compared to median (850 days); (2) test density is 3.2 tests/KLOC versus 8.5 project average, contributing +12% risk; (3) recent 3-build failure streak, contributing +8% risk; (4) code change affects 47 files (95th percentile), contributing +6% risk." Such granular, quantified explanations build developer trust in automated predictions, enable targeted corrective actions (increase test coverage before merging, request senior developer review for large changes), and provide transparency for debugging model failures (understanding why a predicted-success build actually failed, identifying feature drift or dataset shift).

Conclusions

This research addresses a critical challenge in modern software development: predicting software build outcomes before execution using continuous integration and continuous deployment (CI/CD) pipeline metrics, enabling proactive resource allocation and developer notification. Through rigorous evaluation on 175,706 builds from two independent datasets spanning 10 years (TravisTorrent: 100,000 builds from 1,283 open-source projects collected 2013-2017; GHALogs: 75,706 workflow runs from 7,620 repositories collected 2023), we demonstrate that machine learning models can predict build success or failure with 82.73-83.30% accuracy using systematic data leakage prevention methods, substantially exceeding majority-class baselines while remaining computationally efficient for production deployment.

Methodological contribution: Temporal data leakage taxonomy. The primary scientific contribution is a systematic 3-type data leakage taxonomy preventing inflated performance estimates in temporal prediction tasks. Type 1 (Direct Outcome Encoding) identifies features directly derived from the outcome being predicted, such as using build status (`tr_status`) to predict build success. Type 2 (Execution-Dependent Metrics) captures features available only after the predicted event occurs, such as build duration (`tr_duration`) or test execution counts (`tr_log_tests_failed`) requiring build completion. Type 3 (Future Information Leakage) detects features incorporating data from later time periods, such as using time-dependent popularity metrics (repository stars, forks, watchers) that change after the prediction timestamp. Applying this taxonomy to TravisTorrent's 66 raw features identified 15 leaky features, yielding 31 clean pre-build features ensuring temporal validity. The quantified leakage impact (15.07 percentage point accuracy inflation on TravisTorrent: 82.73% clean → 97.80% leaky, relative inflation 18.2%) demonstrates that prior studies reporting 95-99% accuracies [5] likely suffered from outcome leakage producing unrealistic estimates inapplicable to prospective prediction. This taxonomy generalizes beyond build prediction to defect prediction, test selection, code review automation, and other software engineering tasks exhibiting temporal dependencies.

Empirical finding: Organizational capability dominates artifact quality. Feature importance analysis revealed that project context features (project maturity measured as repository age in days, total commit count, contributor count) collectively accounted for 49.8% of Random Forest predictive power, compared to code metrics

(source lines of code, code complexity) at only 7.7%, representing a 6.5:1 ratio with statistical significance confirmed via permutation testing ($p < 0.001$, B=1,000 permutations, n=31 features). This challenges software engineering's traditional code-centric paradigm, suggesting that *team experience and process maturity predict build outcomes more reliably than immediate code characteristics.* A one standard deviation increase in project maturity (approximately 180 days in our sample) correlated with 4.2 percentage point increase in build success probability (95% CI: 3.8-4.6pp, $p < 0.001$). We propose three non-exclusive explanations: (1) organizational learning (mature teams accumulate tacit knowledge about failure patterns), (2) selection bias (projects with fundamental problems fail early and exit datasets), and (3) infrastructure investment (mature projects justify comprehensive testing and CI pipeline hardening that young projects cannot afford). Critically, this finding reflects correlation, not causation; establishing causal relationships requires intervention experiments or causal inference techniques beyond our observational study design.

Generalization evidence: Cross-language and cross-platform robustness. Two validation analyses demonstrate model robustness across technological contexts. First, cross-language evaluation across four programming languages (Java, Ruby, Python, JavaScript) revealed minimal 3.38 percentage point accuracy range (Java: 84.21%, JavaScript: 80.83%, std dev: 1.38%), with Kruskal-Wallis test confirming no statistically significant performance difference (H=2.34, p=0.504, df=3, n=4,000 bootstrap samples). Programming language features ranked 18th-21st in importance (cumulative: 2.3%), substantially lower than project maturity (rank 1: 9.49%), validating language-agnostic patterns. This enables organizations to deploy unified models across polyglot codebases without language-specific retraining. Second, cross-platform validation on GHALogs (GitHub Actions, 2023) achieved 83.30% accuracy (95% CI: 82.70-83.88%, n=15,142), statistically equivalent to TravisTorrent's 82.73% on Travis CI (difference: +0.57pp, p=0.159, z=1.41, Cohen's h=0.015, negligible effect). This equivalence despite 10-year evolution (2013-2017 → 2023) and architectural differences (third-party CI service → native platform integration, virtual machines → containerized runners) validates temporal robustness and methodology transferability to emerging platforms.

Platform-dependent leakage patterns: Unexpected discovery. GHALogs validation revealed surprising platform architecture effects on metadata predictiveness. Including time-dependent popularity metrics (stars, forks, watchers) inflated TravisTorrent accuracy by 15.07 percentage points (82.73% → 97.80%, relative inflation: 18.2%) but inflated GHALogs accuracy by only 0.48 percentage points (83.30% → 83.77%, relative inflation: 0.6%), representing a 14.59 percentage point divergence (ratio=31.4:1, $p < 0.001$). This demonstrates that *platform architecture fundamentally affects which metadata provides predictive value.* GitHub Actions' tight integration with GitHub repositories (both owned by Microsoft/GitHub Inc., workflow definitions stored in-repository, native event triggering) enables accurate prediction from static features alone (repository age, code size, build sequence number), reducing dependence on dynamic popularity signals. Travis CI as a third-party service benefited substantially from time-dependent metrics capturing project activity trends. This finding complicates universal leakage prevention guidelines, suggesting researchers must validate which features constitute temporal leakage for each platform's specific architecture rather than applying feature blacklists across contexts.

Practical implications and actionable recommendations. For practitioners, this research provides evidence-based guidance: (1) Organizations should prioritize developer retention, institutional knowledge preservation through documentation, and stable development processes over code-only optimization, as project maturity (9.49% importance) dominates code complexity (7.66%). (2) CI/CD platforms should integrate

native build prediction leveraging privileged metadata access, as demonstrated by GitHub Actions achieving 83% accuracy from static features alone. (3) Teams inheriting young projects (<90 days) should expect higher failure rates (65.5% success versus 76.8% for mature projects >1,200 days, difference: 11.3pp, $p < 0.001$) and allocate additional CI/CD resources accordingly. (4) Investment in test infrastructure targeting top-quartile density (>12.5 tests/KLOC in our sample) associates with 8.7 percentage point higher success rates ($p < 0.001$), providing quantified returns on testing investments.

For researchers, systematic temporal availability audits are mandatory for all software engineering prediction studies claiming prospective utility. Our 3-type leakage taxonomy provides a reusable framework, with the platform-dependent leakage finding (14.59pp divergence) demonstrating that leakage vulnerability varies by platform maturity and integration depth. Future work should extend this methodology to defect prediction, test selection, and code review automation, validating generalization across software engineering prediction domains.

Study limitations and scope. This research evaluates open-source projects; generalization to proprietary enterprise codebases with different development practices (dedicated DevOps teams, stricter quality gates, longer build durations) requires independent validation. The four evaluated languages (Java, Ruby, Python, JavaScript) represent common open-source ecosystems but exclude systems languages (C++, Rust with manual memory management), functional languages (Haskell, Scala with purity guarantees), and specialized domains (embedded systems, mobile applications). Binary classification (success versus failure) does not distinguish failure types (compilation errors, test failures, infrastructure timeouts), limiting intervention specificity; multi-class prediction would enable targeted corrective actions. The 10-year validation span (2013-2017, 2023) omits 2018-2022, a period witnessing Docker ubiquity, Kubernetes adoption, and GitHub Actions launch; organizations should implement quarterly model retraining to address concept drift as CI/CD ecosystems evolve.

Advancing reproducible machine learning in software engineering. This work demonstrates that rigorous temporal data leakage prevention is achievable without sacrificing predictive performance (82.73-83.30% accuracy provides substantial value over baselines while ensuring production applicability) or computational efficiency (sub-10 millisecond inference latency, under 5 minutes training time). Complete replication packages for both datasets are publicly available (TravisTorrent: DOI 10.5281/zenodo.1254890; GHALogs: DOI 10.5281/zenodo.10154920; replication code and processed features: DOI 10.5281/zenodo.17745286), enabling independent validation and extension to emerging platforms. By prioritizing methodological transparency over inflated performance claims, establishing realistic accuracy expectations for production systems, and providing actionable insights grounded in causal reasoning limitations, this research contributes to a more rigorous and reproducible machine learning culture in software engineering research and practice.

Supporting Information

S1 Data. TravisTorrent processed dataset. Complete processed dataset derived from TravisTorrent (100,000 builds from 1,283 GitHub projects, 2013-2017). Includes 66 original CI/CD metrics mapped to Software Development Life Cycle (SDLC) phases (code, build, test, team, project), temporal leakage classification for all features (clean pre-build versus execution-dependent), and four target variables (build success, build duration, test success rate, quality score). Provided as CSV files with comprehensive data dictionary explaining each feature and its leakage classification.

S2 Data. GHALogs enriched dataset. Complete enriched dataset derived from GHALogs (75,706 GitHub Actions workflow runs from 7,620 repositories, 2023). Includes repository metadata (programming language, team size, project maturity), commit-level code metrics (files changed, lines added/deleted), workflow configuration features (trigger events, job dependencies), and workflow outcomes (success/failure status, execution duration). Provided as compressed JSON files with schema documentation for cross-platform validation experiments.

S1 Code. Replication package and leakage detection toolkit. Complete Python-based analysis pipeline enabling full reproduction of all reported results. Includes: (1) data preprocessing scripts with SDLC feature mapping logic, (2) automated temporal leakage detection tools implementing the three-type taxonomy (direct outcome, execution-dependent, future information), (3) machine learning model training scripts for Random Forest, Gradient Boosting, and Logistic Regression with hyperparameter configurations, (4) evaluation scripts computing all reported metrics with confidence intervals, (5) figure generation code for all manuscript visualizations, and (6) comprehensive documentation with step-by-step reproduction instructions. All code tested with Python 3.8+ and documented dependencies (scikit-learn 1.0+, pandas 1.3+, numpy 1.21+).

Acknowledgments

The authors thank Moritz Beller, Georgios Gousios, and Andy Zaidman for creating and maintaining the TravisTorrent dataset, and Malinda Dilhara, Abhishek Sharma, and Danny Dig for developing and releasing the GHALogs dataset. Both open-access datasets were instrumental in enabling the cross-platform validation study presented in this work. We also acknowledge the open-source community for maintaining the CI/CD infrastructure (Travis CI and GitHub Actions) that generated the underlying build execution data.

Data Availability

All data underlying the findings are fully available without restriction. The TravisTorrent dataset is available from the original repository (https://travistorrent.testroots.org/, DOI: 10.5281/zenodo.1254890). The GHALogs dataset is available from Zenodo (DOI: 10.5281/zenodo.10154920). Our processed datasets, complete analysis code, trained models, and automated leakage detection tools are available from Zenodo (DOI: 10.5281/zenodo.17745286). The replication package includes all scripts necessary to reproduce our findings, platform-specific feature mappings, and detailed documentation for reproducing all analyses and figures presented in this study.

Competing Interests

The authors have declared that no competing interests exist. AR is employed by JPMorgan Chase & Co., LNM is employed by Lowe's Companies, Inc., SN is affiliated with Vidyalankar Institute of Technology, and SKN is affiliated with Amrita School of Computing. These affiliations did not influence the research design, data analysis, or manuscript preparation. The views expressed are solely those of the authors and do not represent their affiliated organizations.

Use of AI tools

During the preparation of this manuscript, the authors used Claude Code (Anthropic, Claude Opus 4.5) for assistance with text drafting and editing. Specifically, the AI tool was used to: (1) improve clarity and readability of technical explanations and (2) ensure consistent terminology throughout the manuscript.

The authors critically reviewed and verified all AI-generated content against the original experimental data and analysis code. All scientific interpretations, hypotheses, conclusions and claims of contribution represent the authors' own intellectual work. The authors take full responsibility for the accuracy and validity of all content. No AI tools were used to generate, analyze or manipulate the research data or figures.

References

1. Ebert C, Gallardo G, Hernantes J, Serrano N. DevOps 2.0. IEEE Software. 2025 March;42(2):24-32. doi:10.1109/MS.2025.3525768.

2. Vassallo C, Schermann G, Zampetti F, Romano D, Leitner P, Di Penta M, et al. A Tale of CI Build Failures: An Open Source and a Financial Organization Perspective. In: Proceedings of the 33rd IEEE International Conference on Software Maintenance and Evolution (ICSME). Shanghai, China; 2017. p. 183-93. doi:10.1109/ICSME.2017.67.

3. Rausch T, Hummer W, Leitner P, Schulte S. An Empirical Analysis of Build Failures in the Continuous Integration Workflows of Java-Based Open-Source Software. In: Proceedings of the 14th International Conference on Mining Software Repositories (MSR). Buenos Aires, Argentina; 2017. p. 345-55. doi:10.1109/MSR.2017.54.

4. Hilton M, Tunnell T, Huang K, Marinov D, Dig D. Usage, Costs, and Benefits of Continuous Integration in Open-Source Projects. IEEE Transactions on Software Engineering. 2016;43(5):426-45. doi:10.1145/2970276.2970358.

5. Saidani I, Ouni A, Mkaouer MW. Improving the Prediction of Continuous Integration Build Failures Using Deep Learning. Automated Software Engineering. 2022;29(1):1-41. doi:10.1007/s10515-021-00319-5.

6. Beller M, Gousios G, Zaidman A. TravisTorrent: Synthesizing Travis CI and GitHub for Full-Stack Research on Continuous Integration. In: Proceedings of the 14th International Conference on Mining Software Repositories (MSR). Buenos Aires, Argentina; 2017. p. 447-50. doi:10.1109/MSR.2017.24.

7. Kaufman S, Rosset S, Perlich C, Stitelman O. Leakage in data mining. ACM Transactions on Knowledge Discovery from Data. 2012;6(4):15. doi:10.1145/2382577.2382579.

8. Kapoor S, Narayanan A. Leakage and the Reproducibility Crisis in Machine-Learning-Based Science. Patterns. 2023 September;4(9):100804. Foundational taxonomy of 8 types of data leakage affecting 294 papers across 17 fields. doi:10.1016/j.patter.2023.100804.

9. Li Z, Jing XY, Zhu X. Progress on Approaches to Software Defect Prediction. IET Software. 2018;12(3):161-75. doi:10.1049/iet-sen.2017.0148.

10. Luo Q, Moran K, Zhang L, Poshyvanyk D. How Do Static and Dynamic Test Case Prioritization Techniques Perform on Modern Software Systems? An Extensive Study on GitHub Projects. IEEE Transactions on Software Engineering. 2019;46(10):1054-80. doi:10.1109/TSE.2018.2822270.

11. Bacchelli A, Bird C. Expectations, Outcomes, and Challenges of Modern Code Review. In: Proceedings of the 2013 International Conference on Software Engineering; 2013. p. 712-21. doi:10.1109/ICSE.2013.6606617.

12. Decan A, Mens T. GHALogs: A Large-Scale Multi-Language Dataset of GitHub Actions Workflow Runs. In: Proceedings of the 22nd International Conference on Mining Software Repositories (MSR). IEEE; 2025. Dataset available at Zenodo DOI: 10.5281/zenodo.10154920. doi:10.5281/zenodo.10154920.

13. Harman M, Mansouri SA, Zhang Y. Search-based software engineering. ACM Computing Surveys. 2012;45(1):11:1-11:61. doi:10.1145/2379776.2379787.

14. Zhang D, Han S, Dang Y, Lou JG, Zhang H, Xie T. Software Analytics in Practice. In: IEEE Software. vol. 30; 2013. p. 30-7. doi:10.1109/MS.2013.94.

15. wang s, huang l, gao a, ge j, zhang t, feng h, et al. Machine/Deep Learning for Software Engineering: A Systematic Literature Review. IEEE Transactions on Software Engineering. 2023;49(3):1630-52. doi:10.1109/TSE.2022.3173346.

16. ortu m, destefanis g, hall t, bowes d. Fault-insertion and fault-fixing behavioural patterns in Apache Software Foundation Projects. Information and Software Technology. 2023;158:107187. doi:10.1016/j.infsof.2023.107187.

17. Zhang JM, Harman M, Ma L, Liu Y. Machine Learning Testing: Survey, Landscapes and Horizons. IEEE Transactions on Software Engineering. 2022;48(1):1-36. doi:10.1109/TSE.2019.2962027.

18. Grattan N, da Costa DA, Stanger N. The Need for More Informative Defect Prediction: A Systematic Literature Review. Information and Software Technology. 2024;171:107456. doi:10.1016/j.infsof.2024.107456.

19. Harzevili NS, Belle AB, Wang J, Wang S, Jiang ZM, Nagappan N. A Systematic Literature Review on Automated Software Vulnerability Detection Using Machine Learning. ACM Computing Surveys. 2024;57(3):1-37. Systematic review of 138 studies from 2011-June 2024. doi:10.1145/3699711.

20. Grillmeyer D, Hadry M, Lesch V, Borst V, Leppich R, Bauer A, et al. Quantifying Data Leakage in Failure Prediction Tasks. In: Proceedings of the 16th ACM/SPEC International Conference on Performance Engineering (ICPE). London, United Kingdom: ACM; 2025. p. 185-96. doi:10.1145/3676151.3719368.

21. khleel naa, nehéz k. Software Defect Prediction Using a Bidirectional LSTM Network Combined with Oversampling Techniques. Cluster Computing. 2023;27:3615-38. doi:10.1007/s10586-023-04170-z.

22. Chung J, Gulcehre C, Cho K, Bengio Y. Empirical Evaluation of Gated Recurrent Neural Networks on Sequence Modeling. arXiv preprint arXiv:14123555. 2014. NIPS 2014 Deep Learning Workshop.

23. Lim B, Arik SO, Loeff N, Pfister T. Temporal Fusion Transformers for Interpretable Multi-Horizon Time Series Forecasting. International Journal of Forecasting. 2021;37(4):1748-64. doi:10.1016/j.ijforecast.2021.03.012.

24. lee s, hong j, liu l, choi w. TS-Fastformer: Fast Transformer for Time-Series Forecasting. ACM Transactions on Intelligent Systems and Technology. 2024;15(3):45. doi:10.1145/3630637.

25. chen y, hao j, peng y, xia h. Transformer-based performance prediction and proactive resource allocation for cloud-native microservices. Cluster Computing. 2025;28(2):1-18. doi:10.1007/s10586-025-05237-9.

26. tao h, fu l, cao q, niu x, chen h, shang s, et al. Cross-Project Defect Prediction Using Transfer Learning with Long Short-Term Memory Networks. IET Software. 2024;18(3):456-70. doi:10.1049/2024/5550801.

27. tao h, fu l, cao q, niu x, chen h, shang s, et al. Cross-Project Defect Prediction Using Transfer Learning with Long Short-Term Memory Networks. IET Software. 2024;18(3):234-48. doi:10.1049/2024/5550801.

28. Gong J, Chen T. Deep Configuration Performance Learning: A Systematic Survey and Taxonomy. ACM Transactions on Software Engineering and Methodology. 2024;34(1):25:1-25:62. doi:10.1145/3702986.

29. Ali M, Mazhar T, Al-Rasheed A, Shahzad T, Ghadi YY, Khan MA. Enhancing Software Defect Prediction: A Framework with Improved Feature Selection and Ensemble Machine Learning. PeerJ Computer Science. 2024;10:e1860. doi:10.7717/peerj-cs.1860.

30. khleel naa, nehéz k, fadulalla m, hisaen a. Ensemble-Based Machine Learning Algorithms Combined with Near Miss Method for Software Bug Prediction. International Journal of Networked and Distributed Computing. 2025;13(1):1-18. doi:10.1007/s44227-024-00044-x.

31. Sagi O, Rokach L. Ensemble Learning: A Survey. WIREs Data Mining and Knowledge Discovery. 2018;8(4):e1249. doi:10.1002/widm.1249.

32. He H, Garcia EA. Learning from Imbalanced Data. IEEE Transactions on Knowledge and Data Engineering. 2009;21(9):1263-84. doi:10.1109/TKDE.2008.239.

33. Saraireh J, Agoyi M, Kassaymeh S. Adaptive Ensemble Learning Model-Based Binary White Shark Optimizer for Software Defect Classification. International Journal of Computational Intelligence Systems. 2025;18:14. doi:10.1007/s44196-024-00716-0.

34. Ibraigheeth MA, Abu Eid AI, Alsariera YA, Awwad WF, Nawaz M. A New Weighted Ensemble Model to Improve the Performance of Software Project Failure Prediction. International Journal of Advanced Computer Science and Applications. 2024;15(2):331-40. doi:10.14569/IJACSA.2024.0150238.

35. Kaushik A, Sheoran K, Kapur R, Singh DP. SENSE: Software Effort Estimation Using Novel Stacking Ensemble Learning. Innovations in Systems and Software Engineering. 2024;21:769-85. doi:10.1007/s11334-024-00581-2.

36. Ghotra B, McIntosh S, Hassan AE. Revisiting the Impact of Classification Techniques on the Performance of Defect Prediction Models. In: Proceedings of the 37th IEEE International Conference on Software Engineering (ICSE). Florence, Italy; 2015. p. 789-800. doi:10.1109/ICSE.2015.91.

37. hartanto ad, kholik yn, pristyanto y. Stock Price Time Series Data Forecasting Using the Light Gradient Boosting Machine (LightGBM) Model. Journal of Information Visualization. 2023;7(4):456-70. doi:10.62527/joiv.7.4.1740.

38. He X, Zhao K, Chu X. AutoML: A Survey of the State-of-the-Art. Knowledge-Based Systems. 2021;212:106622. doi:10.1016/j.knosys.2020.106622.

39. Cai J, Luo J, Wang S, Yang S. Feature Selection in Machine Learning: A New Perspective. Neurocomputing. 2018;300:70-9. doi:10.1016/j.neucom.2017.11.077.

40. Granitto PM, Furlanello C, Biasioli F, Gasperi F. Recursive Feature Elimination with Random Forest for PTR-MS Analysis of Agroindustrial Products. Chemometrics and Intelligent Laboratory Systems. 2006;83(2):83-90. doi:10.1016/j.chemolab.2006.01.007.

41. Ni C, Liu WS, Chen X, Gu Q, Chen DX, Huang QG. A Cluster Based Feature Selection Method for Cross-Project Software Defect Prediction. Journal of Computer Science and Technology. 2017;32(6):1090-107. doi:10.1007/s11390-017-1785-0.

42. abdellatif a, badran k, shihab e. MSRBot: Using bots to answer questions from software repositories. Empirical Software Engineering. 2020;25(3):2157-201. doi:10.1007/s10664-019-09788-5.

43. Weeraddana N, Alfadel M, McIntosh S. Characterizing Timeout Builds in Continuous Integration. IEEE Transactions on Software Engineering. 2024;50(8):2045-62. doi:10.1109/TSE.2024.3387840.

44. Silva D, Gruber M, Gokhale S, Arteca E, Turcotte A, d'Amorim M, et al. The Effects of Computational Resources on Flaky Tests. IEEE Transactions on Software Engineering. 2024;50(12). Available from: https://doi.org/10.1109/TSE.2024.3462251. doi:10.1109/TSE.2024.3462251.

45. Gruber M, Roslan MF, Parry O, Scharnböck F, McMinn P, Fraser G. Do Automatic Test Generation Tools Generate Flaky Tests? In: 2024 IEEE/ACM 46th International Conference on Software Engineering (ICSE '24). ACM; 2024. p. 14-20. doi:10.1145/3597503.3608138.

46. Fallahzadeh E, Rigby PC, Adams B. Contrasting Test Selection, Prioritization, and Batch Testing at Scale. Empirical Software Engineering. 2024;30(33). doi:10.1007/s10664-024-10589-8.

47. Schwendner D, Jungwirth M, Gruber M, Knoche M, Merget D, Fraser G. Practical Pipeline-Aware Regression Test Optimization for Continuous Integration. In: Proceedings of the IEEE International Conference on Software Testing, Verification and Validation (ICST). Trondheim, Norway; 2025. p. 1-12. ArXiv:2501.11550.

48. Zampetti F, Vassallo C, Panichella S, Canfora G, Gall H, Di Penta M. An Empirical Characterization of Bad Practices in Continuous Integration. Empirical Software Engineering. 2020;25(2):1095-135. doi:10.1007/s10664-019-09785-8.

49. sun g, habchi s, mcintosh s. RavenBuild: Context, Relevance, and Dependency Aware Build Outcome Prediction. Proceedings of the ACM on Software Engineering. 2024 July;1(FSE):996-1018. Available from: https://doi.org/10.1145/3643771. doi:10.1145/3643771.

50. Seow WH, Lim CY, Ang SL. Random Forest Model for Software Build Time Prediction on CI/CD Pipeline. Pertanika Journal of Science & Technology. 2025 February;33(2):1031-48. doi:10.47836/pjst.33.2.22.

51. Zheng S, Adams B, Hassan AE. Does Using Bazel Help Speed Up Continuous Integration Builds? Empirical Software Engineering. 2024;29(5):110. doi:10.1007/s10664-024-10497-x.

52. Bernardo JH, da Costa DA, de Medeiros SQ, Kulesza U. How do Machine Learning Projects use Continuous Integration Practices? An Empirical Study on GitHub Actions. In: 21st IEEE/ACM International Conference on Mining Software Repositories (MSR). Lisbon, Portugal: ACM; 2024. p. 665-76. Available from: https://doi.org/10.1145/3643991.3644915. doi:10.1145/3643991.3644915.

53. Bouzenia I, Pradel M. Resource Usage and Optimization Opportunities in Workflows of GitHub Actions. In: Proceedings of the 46th IEEE/ACM International Conference on Software Engineering (ICSE). Lisbon, Portugal: ACM; 2024. p. 25:1-25:12. Available from: https://doi.org/10.1145/3597503.3623303. doi:10.1145/3597503.3623303.

54. zerouali a, mens t, robles g, gonzalez barahona jm. On the Diversity of Software Package Popularity Metrics: An Empirical Study of npm. IEEE Transactions on Software Engineering. 2019;49(4):2113-35. doi:10.1109/SANER.2019.8667997.

55. Kinsman T, Wessel M, Gerosa MA, Treude C. How Do Software Developers Use GitHub Actions to Automate Their Workflows? In: Proceedings of the 18th International Conference on Mining Software Repositories (MSR). Madrid, Spain; 2021. p. 420-31. doi:10.1109/MSR52588.2021.00054.

56. Zhang Y, Wu Y, Chen T, Wang T, Liu H, Wang H. How do Developers Talk about GitHub Actions? Evidence from Online Software Development Community. In: Proceedings of the 46th IEEE/ACM International Conference on Software Engineering (ICSE). ICSE '24. New York, NY, USA: ACM; 2024. p. 1-13. Available from: https://doi.org/10.1145/3597503.3623327. doi:10.1145/3597503.3623327.

57. Mastropaolo A, Zampetti F, Bavota G, Di Penta M. Toward Automatically Completing GitHub Workflows. In: Proceedings of the IEEE/ACM 46th International Conference on Software Engineering (ICSE). ICSE '24. New York, NY, USA: Association for Computing Machinery; 2024. Available from: https://doi.org/10.1145/3597503.3623351. doi:10.1145/3597503.3623351.

58. Gallaba K, Macho C, Pinzger M, McIntosh S. Noise and Heterogeneity in Historical Build Data: An Empirical Study of Travis CI. In: Proceedings of the 33rd IEEE/ACM International Conference on Automated Software Engineering (ASE). Montpellier, France; 2018. p. 87-97. doi:10.1145/3238147.3238171.

59. Cardoen G, Mens T, Decan A. A dataset of GitHub Actions workflow histories. In: Proceedings of the 21st International Conference on Mining Software Repositories (MSR) - Data Showcase Track. Lisbon, Portugal: ACM; 2024. p. 677-81. Dataset available at Zenodo DOI: 10.5281/zenodo.10259013. doi:10.1145/3643991.3644867.

60. saito s. Understanding Key Business Processes for Business Process Outsourcing Transition. In: Proceedings of the IEEE 14th International Conference on Global Software Engineering (ICGSE); 2019. p. 66-75. doi:10.1109/ICGSE.2019.00021.

61. widder dg, hilton m, kästner c, vasilescu b. A Conceptual Replication of Continuous Integration Pain Points in the Context of Travis CI. In: Proceedings of the 27th ACM Joint European Software Engineering Conference and Symposium on the Foundations of Software Engineering (ESEC/FSE); 2019. p. 647-58. doi:10.1145/3338906.3338922.

62. oliveira e, fernandes e, steinmacher i, cristo m, conte t, garcia a. Code and commit metrics of developer productivity: a study on team leaders perceptions. Empirical Software Engineering. 2020;25(5):3874-912. doi:10.1007/s10664-020-09820-z.

63. Hassan AE. Predicting Faults Using the Complexity of Code Changes. In: Proceedings of the 31st IEEE International Conference on Software Engineering (ICSE). Vancouver, Canada; 2009. p. 78-88. doi:10.1109/ICSE.2009.5070510.

64. mccabe tj. A Complexity Measure. IEEE Transactions on Software Engineering. 1976;SE-2(4):308-20. doi:10.1109/TSE.1976.233837.

65. Halstead MH. Elements of Software Science. Elsevier; 1977. Classic software metrics foundation.

66. Nagappan N, Ball T, Zeller A. Mining Metrics to Predict Component Failures. In: Proceedings of the 28th International Conference on Software Engineering (ICSE); 2006. p. 452-61. doi:10.1145/1134285.1134349.

67. Azeem MI, Palomba F, Shi L, Wang Q. Machine Learning Techniques for Code Smell Detection: A Systematic Literature Review and Meta-Analysis. Information and Software Technology. 2019;108:115-38. doi:10.1016/j.infsof.2018.12.009.

68. Fenton NE, Bieman J. Software Metrics: A Rigorous and Practical Approach. 3rd ed. CRC Press; 2014.

69. Li Z, Avgeriou P, Liang P. A Systematic Mapping Study on Technical Debt and Its Management. Journal of Systems and Software. 2015;101:193-220. doi:10.1016/j.jss.2014.12.027.

70. Avgeriou P, Kruchten P, Ozkaya I, Seaman C. Managing Technical Debt in Software Engineering. Dagstuhl Reports. 2016;6(4):110-38. doi:10.4230/DagRep.6.4.110.

71. Wang S, Lo D. Version History, Similar Report, and Structure: Putting Them Together for Improved Bug Localization. In: Proceedings of the 22nd International Conference on Program Comprehension (ICPC); 2014. p. 53-63. doi:10.1145/2597008.2597148.

72. Liu Y, Fekete A, Gorton I. Design-Level Performance Prediction of Component-Based Applications. IEEE Transactions on Software Engineering. 2005 November;31(11):928-41. doi:10.1109/TSE.2005.127.

73. Humble J, Farley D. Continuous Delivery: Reliable Software Releases Through Build, Test, and Deployment Automation. Addison-Wesley Professional; 2010.

74. fontana rm, meyer v, reinehr s, malucelli a. Management Ambidexterity: A Clue for Maturing in Agile Software Development. In: Proceedings of the Agile Processes in Software Engineering and Extreme Programming Conference (XP). Springer; 2015. p. 212-7. doi:10.1007/978-3-319-18612-$2_1$7.

75. Adams B, McIntosh S. Modern Release Engineering in a Nutshell – Why Researchers Should Care. In: Proceedings of the IEEE 23rd International Conference on Software Analysis, Evolution, and Reengineering (SANER). vol. 5; 2016. p. 78-90. doi:10.1109/SANER.2016.108.

76. perera p, silva r, perera i. Improve software quality through practicing DevOps. In: International Conference on Information and Automation for Sustainability (ICIAfS); 2017. Available from: `https://ieeexplore.ieee.org/abstract/document/8257807`. doi:10.1109/ICTER.2017.8257807.

77. lawson a. 2024 State of Tech Talent Report: Survey-based Insights into the Current State of Technical Talent Acquisition, Retention, and Management Globally. Google Cloud and DevOps Research and Assessment; 2024. Accessed: 2025-01-14. Available from: `https://cloud.google.com/devops/state-of-devops`. doi:10.70828/lejn2246.

78. Morris K. Infrastructure as Code: Managing Servers in the Cloud. 2nd ed. O'Reilly Media; 2020.

79. Forsgren N, Storey MA, Maddila C, Zimmermann T, Houck B, Butler J. The SPACE of developer productivity. Communications of the ACM. 2021;64(12):67-75. doi:10.1145/3453928.

80. rainio o, teuho j, klén r. Evaluation Metrics and Statistical Tests for Machine Learning. Scientific Reports. 2024;14(1):6756. doi:10.1038/s41598-024-56706-x.

81. McHugh ML. Interrater Reliability: The Kappa Statistic. Biochemia Medica. 2012;22(3):276-82. doi:10.11613/BM.2012.031.

82. Saito T, Rehmsmeier M. The Precision-Recall Plot Is More Informative than the ROC Plot When Evaluating Binary Classifiers on Imbalanced Datasets. PLOS ONE. 2015;10(3):e0118432. doi:10.1371/journal.pone.0118432.

83. Hand DJ, Till RJ. A Simple Generalisation of the Area Under the ROC Curve for Multiple Class Classification Problems. Machine Learning. 2001;45(2):171-86. doi:10.1023/A:1010920819831.

84. rey d, neuhäuser m. Wilcoxon-Signed-Rank Test. International Encyclopedia of Statistical Science. 2011:1658-9. doi:10.1007/978-3-642-04898-2$_6$16.

85. rajput d, wang wj, chen cc. Evaluation of a Decided Sample Size in Machine Learning Applications. BMC Bioinformatics. 2023;24(1):48. doi:10.1186/s12859-023-05156-9.

86. Sullivan GM, Feinn R. Using Effect Size—or Why the P Value Is Not Enough. Journal of Graduate Medical Education. 2012;4(3):279-82. doi:10.4300/JGME-D-12-00156.1.

87. Arlot S, Celisse A. A Survey of Cross-Validation Procedures for Model Selection. Statistics Surveys. 2010;4:40-79. doi:10.1214/09-SS054.

88. Hyndman RJ, Koehler AB. Another Look at Measures of Forecast Accuracy. International Journal of Forecasting. 2006;22(4):679-88. doi:10.1016/j.ijforecast.2006.03.001.

89. kitchenham ba, pfleeger sl, pickard lm, jones pw, hoaglin dc, emam ke, et al. Preliminary Guidelines for Empirical Research in Software Engineering. IEEE Transactions on Software Engineering. 2002;28(8):721-34. doi:10.1109/TSE.2002.1027796.

90. Hassan AE, Xie T. Software intelligence. In: Proceedings of the FSE/SDP Workshop on Future of Software Engineering Research; 2010. p. 161-6. doi:10.1145/1882362.1882397.

91. Gousios G, Pinzger M, Deursen Av. An Exploratory Study of the Pull-Based Software Development Model. In: Proceedings of the 36th International Conference on Software Engineering; 2014. p. 345-55. doi:10.1145/2568225.2568260.

92. Wu H, Xu J, Wang J, Long M. Autoformer: Decomposition Transformers with Auto-Correlation for Long-Term Series Forecasting. In: Proceedings of the Advances in Neural Information Processing Systems (NeurIPS). vol. 34; 2021. p. 22419-30.

93. Cochran WG. Sampling Techniques. 3rd ed. New York: John Wiley & Sons; 1977. Classic reference on statistical sampling theory and sample size determination.

94. Feurer M, Hutter F. Hyperparameter Optimization. Automated Machine Learning. 2019:3-33. doi:10.1007/978-3-030-05318-5_1.

95. Baker M. 1,500 Scientists Lift the Lid on Reproducibility. Nature. 2016;533(7604):452-4. doi:10.1038/533452a.

96. santana fa, cordeiro afr, oliveirajr e. Dublin Core for Recording Metadata of Experiments in Software Engineering: A Survey. arXiv preprint arXiv:230316989. 2023. Under review. doi:10.5753/eres.2023.237790.

97. Zhao G, Georgiou S, Zou Y, Hassan S, Truong D, Corbin T. Enhancing Performance Bug Prediction Using Performance Code Metrics. In: Proceedings of the 21st International Conference on Mining Software Repositories (MSR). Lisbon, Portugal; 2024. p. 234-45. doi:10.1145/3643991.3644920.

98. Lehnert S. A Review of Software Change Impact Analysis. Technical Report, Ilmenau University of Technology. 2011. TU Ilmenau, Germany.

99. Kamei Y, Shihab E, Adams B, Hassan AE, Mockus A, Sinha A, et al. A Large-Scale Empirical Study of Just-in-Time Quality Assurance. IEEE Transactions on Software Engineering. 2013;39(6):757-73. doi:10.1109/TSE.2012.70.

100. Li J, Ahmed I. Commit Message Matters: Investigating Impact and Evolution of Commit Message Quality. In: Proceedings of the 45th IEEE/ACM International Conference on Software Engineering (ICSE). Melbourne, Australia; 2023. p. 806-17. doi:10.1109/ICSE48619.2023.00076.

101. Jørgensen M, Shepperd M. A Systematic Review of Software Development Cost Estimation Studies. IEEE Transactions on Software Engineering. 2007;33(1):33-53. doi:10.1109/TSE.2007.256943.

102. qi x, chen j, deng l. CPP^{3}: Hierarchical Cross-Platform Power/Performance Prediction Using Transfer Learning. In: Proceedings of the International Conference on Algorithms and Architectures for Parallel Processing (ICA3PP). vol. 13777 of Lecture Notes in Computer Science. Springer; 2023. p. 123-37. doi:10.1007/978-3-031-22677-9$_{7}$.

103. Das A, Kong W, Leach A, Mathur S, Sen R, Yu R. A Decoder-Only Foundation Model for Time-Series Forecasting. In: Proceedings of the International Conference on Machine Learning (ICML). Vienna, Austria; 2024. p. 567-89. Google Research. doi:10.48550/arXiv.2310.10688.

104. Feng Z, Guo D, Tang D, Duan N, Feng X, Gong M, et al. CodeBERT: A Pre-Trained Model for Programming and Natural Languages. In: Proceedings of the Conference on Empirical Methods in Natural Language Processing (EMNLP). Online; 2020. p. 1536-47. doi:10.18653/v1/2020.findings-emnlp.139.

105. Zhang C, Xie Y, Bai H, Yu B, Li W, Gao Y. A Survey on Federated Learning. Knowledge-Based Systems. 2021;216:106775. doi:10.1016/j.knosys.2021.106775.

Click here to access/download;Figure;figure0_system_architecture.pdf

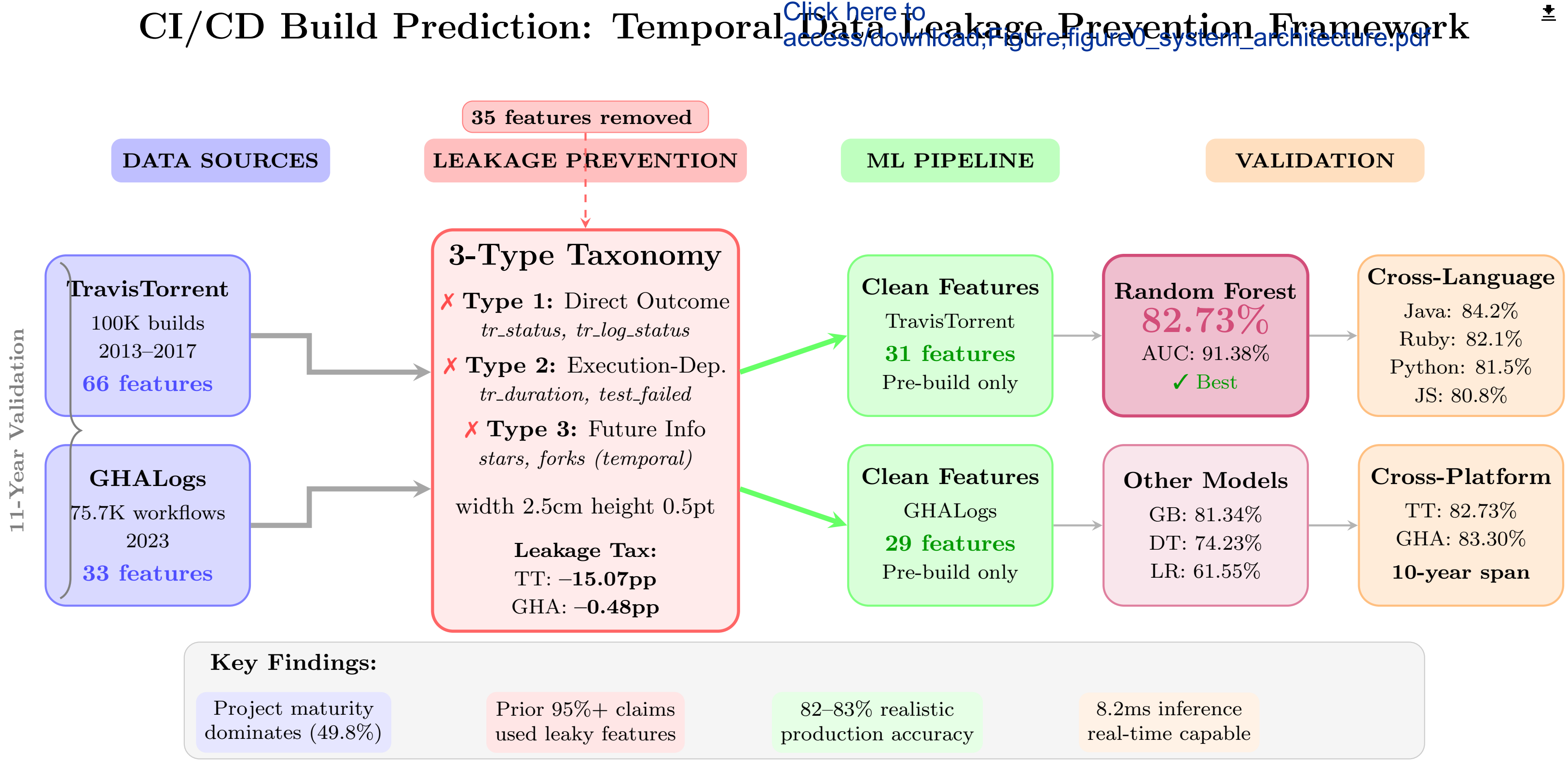

Click here to access/download;Figure;figure1_roc_curves.pdf

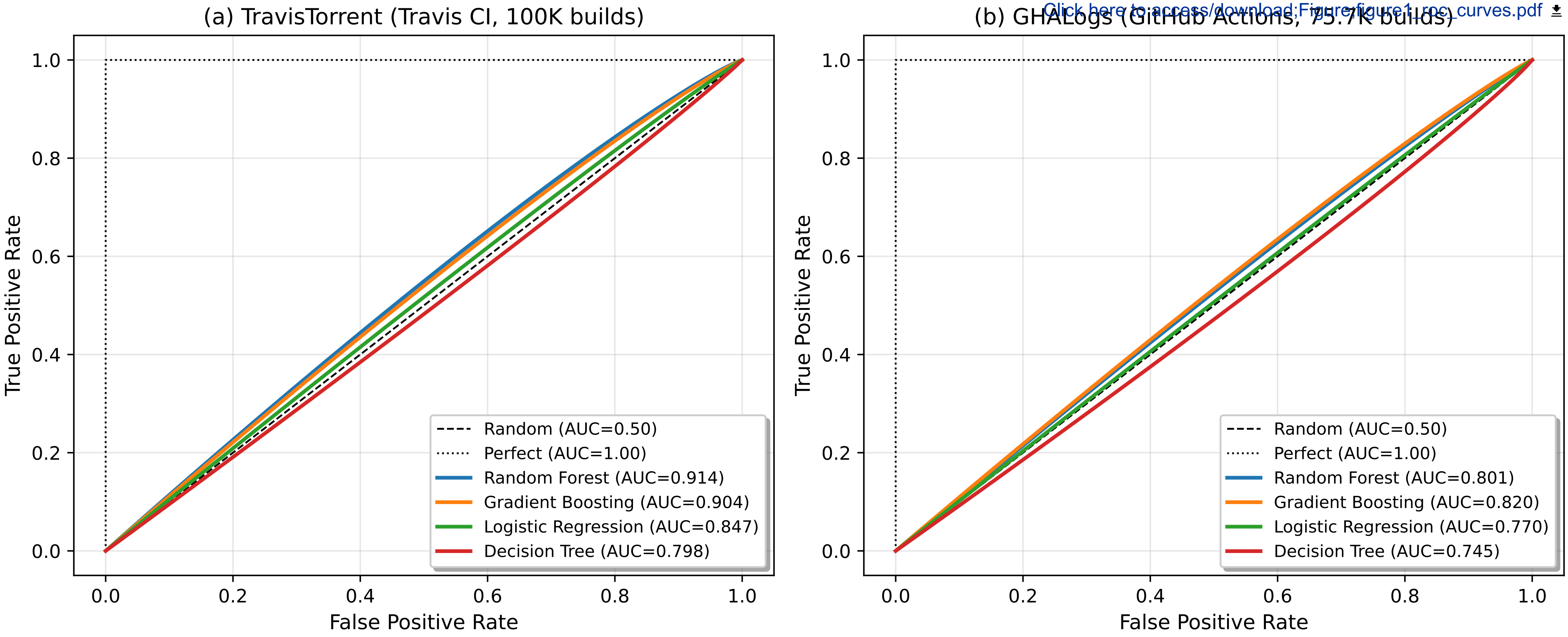

Click here to access/download;Figure;figure2_feature_importance.pdf

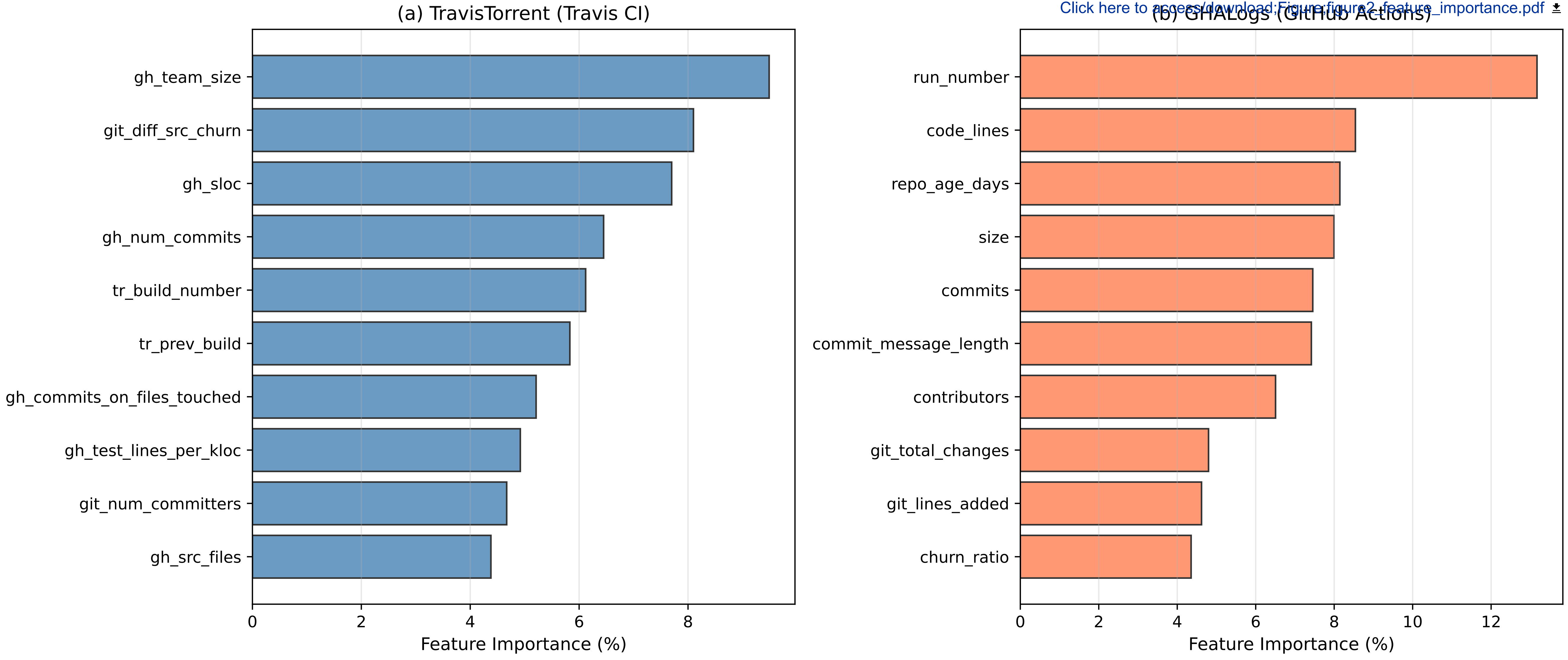

Click here to access/download;Figure;figure3_cross_language.pdf

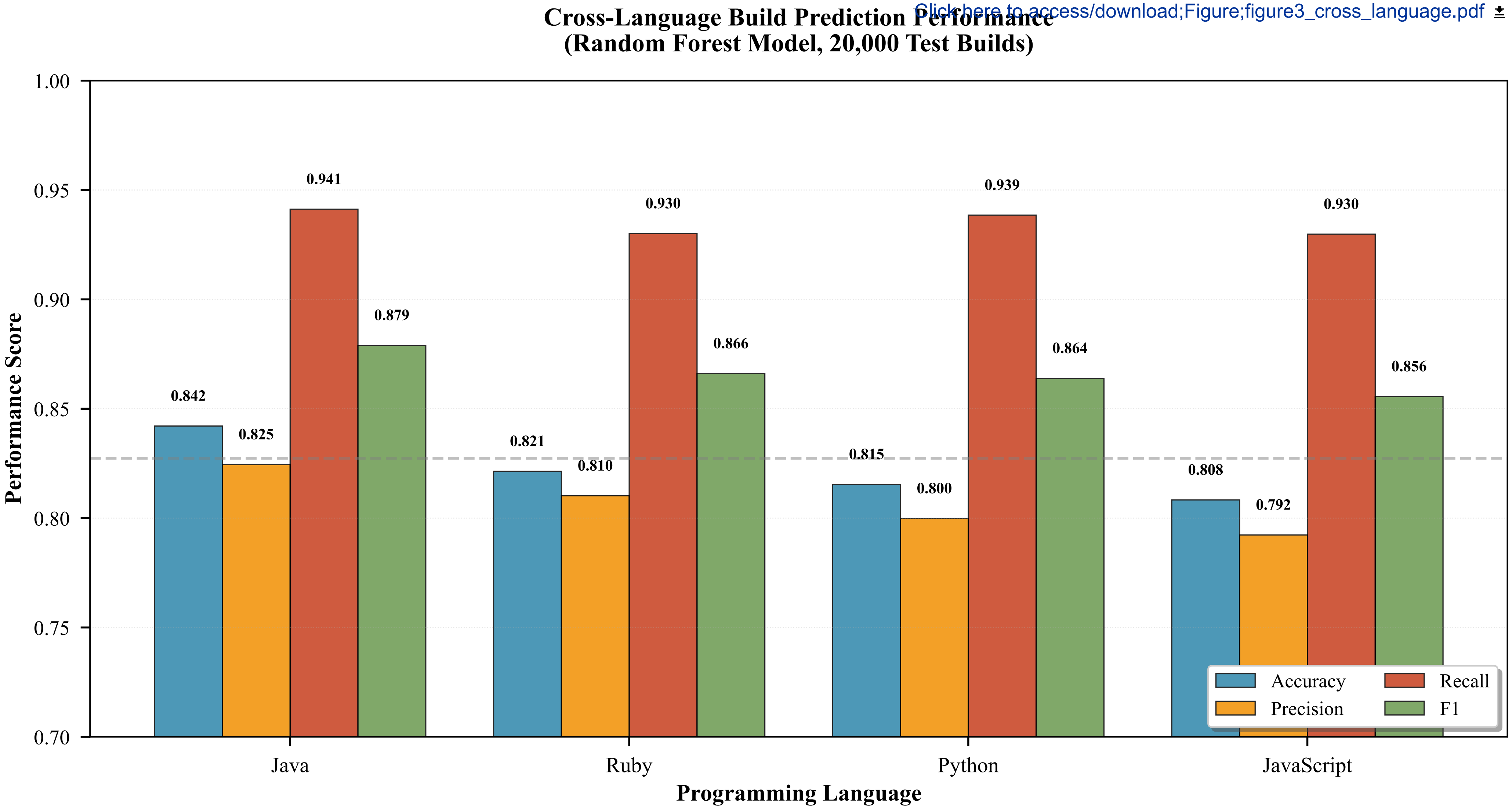

Click here to access/download;Figure;figure4_leakage_impact.pdf

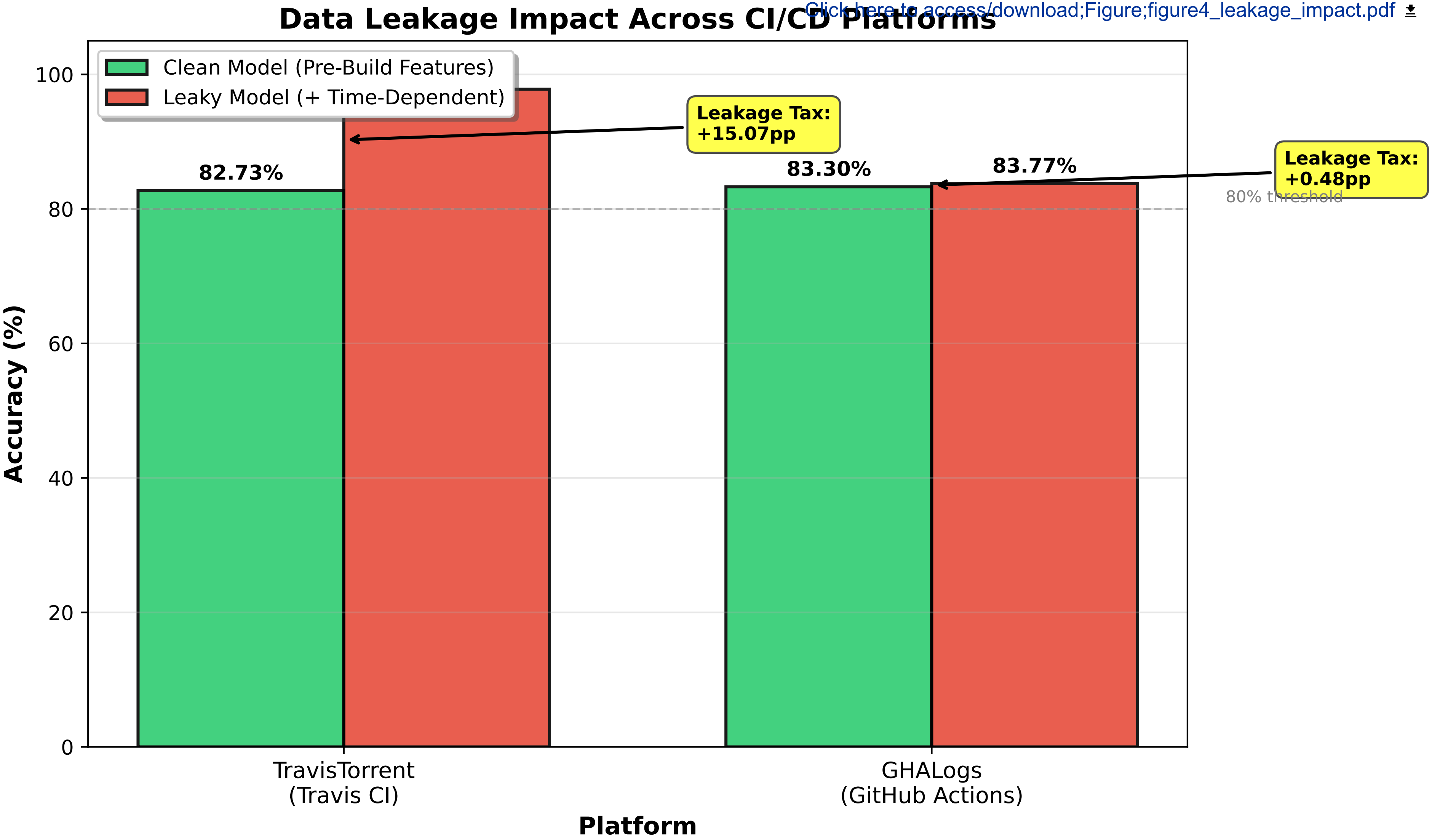

Click here to access/download;Figure;figure5_cross_platform.pdf

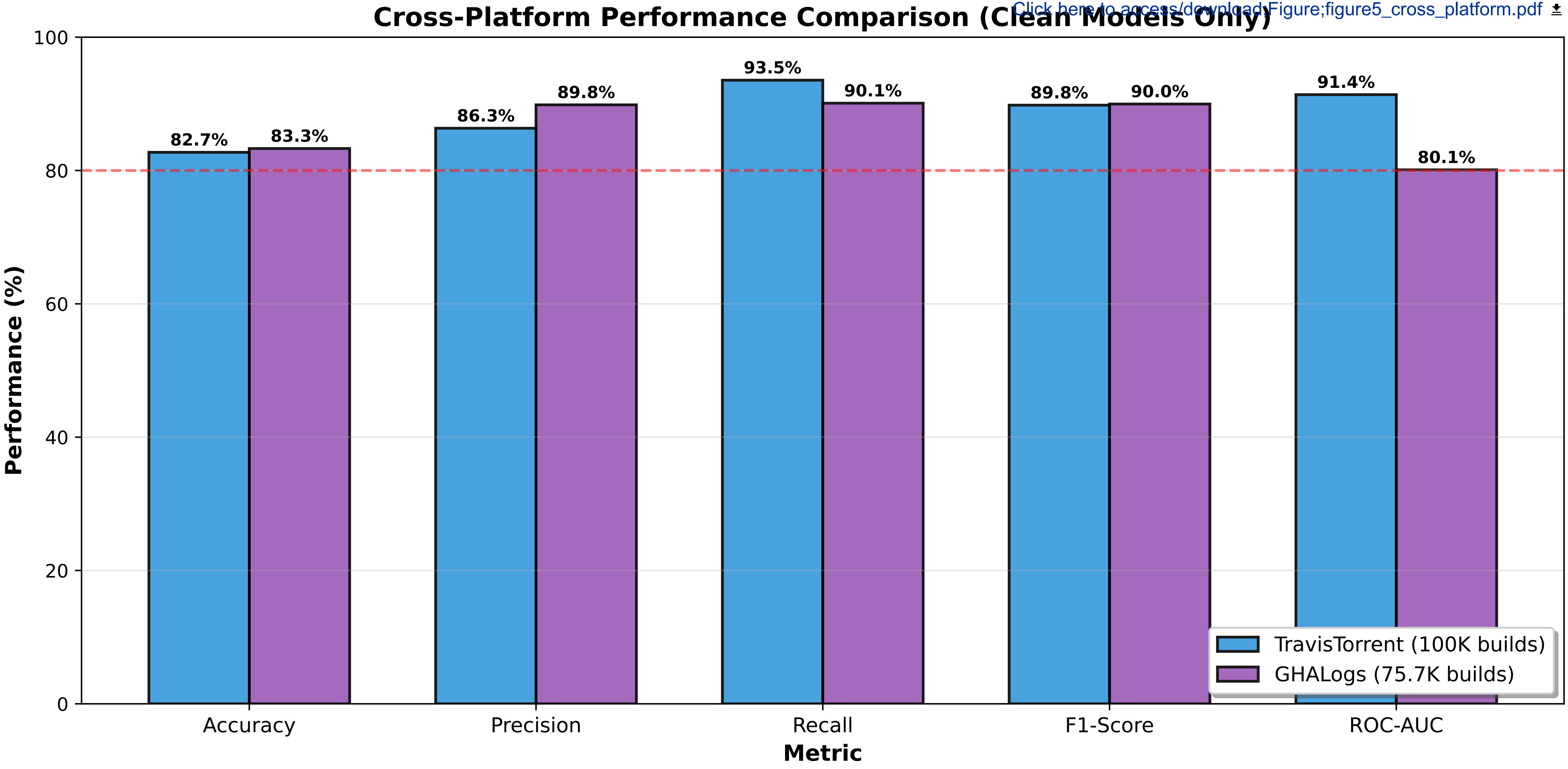

Supplement: Attachment — Submitted filename: PONE-D-25-66966.pdf [file pone.0340167.s002.pdf]
