# Supplementary material for: A taxonomy for detecting and preventing temporal data leakage in machine learning-based build prediction: A dual-platform empirical validation
Source: PLoS One. 2026 May 27;21(5):e0340167. doi: 10.1371/journal.pone.0340167 (PMC13215522; doi:10.1371/journal.pone.0340167)
Supplement: Attachment — Submitted filename: response_to_reviewers.pdf [file pone.0340167.s003.pdf]

Response to Reviewers

Manuscript ID: PONE-D-25-66966

A taxonomy for detecting and preventing temporal data leakage in machine learning-based build prediction: A dual-platform empirical validation

Lalit Narayan Mishra, Amit Rangari, Sandesh Nagrare, Saroj Kumar Nayak

February 20, 2026

Dear Editor and Reviewers,

Thank you for the thorough and constructive review of our manuscript PONE-D-25-66966. The feedback from both reviewers has substantially improved the manuscript's conciseness, rigor, and presentation quality. All comments have been addressed in full.

This response letter is organized as follows:

1. A summary of major changes
2. Point-by-point responses to Reviewer #1 (8 comments)
3. Point-by-point responses to Reviewer #2 (48 items)

Line numbers reference the clean manuscript PDF. A tracked-changes version with yellow highlighting and margin annotations mapping each change to its reviewer comment is also provided.

Summary of Major Changes

The revision addresses all 56 reviewer comments (8 from Reviewer #1, 48 from Reviewer #2). The principal changes are:

- **Manuscript reduced from 54 to 31 pages.** Tutorial-style prose, redundant enumerations restating table contents, and detailed statistical re-comparisons in the Conclusion were removed. Five future research paragraphs were converted to a concise bulleted list.
- **9 new tables added (7 original → 16 total).** Tables now present: evaluation metrics, statistical methods, leakage taxonomy, clean vs. leaky features, cost-sensitive analysis, SDLC distribution, key findings, statistical summary, and stakeholder implications. All 8 Results-section enumerate blocks were replaced with concise paragraphs that reference tables.
- **1 new figure added (6 original → 7 total).** Precision-recall curves (Fig 6) with dual-panel comparison: TravisTorrent (4 models, RF AP=0.938) and GHALogs (clean AP=0.946 vs. leaky AP=0.951).
- **MCC and cost-sensitive analysis added.** Matthews Correlation Coefficient added to evaluation metrics and model comparison tables. Cost-sensitive analysis table with optimal thresholds at FN:FP ratios of 1:1, 5:1, 10:1, and 20:1. Both ML pipelines re-run with updated metrics.
- **Introduction restructured from 5 subsections to 4 focused subsections.** New flow: Background and Motivation → The Methodological Gap → Approach → Research Contributions. Tutorial-style

CI/CD explanations and protein folding/medical analogies removed.

- **All research questions removed.** The "Research Questions" subsection (defining RQ1–RQ5) was deleted. All 5 Results headings changed to descriptive titles. All inline RQ references removed.
- **Related Work trimmed.** Tangential citations (generic ML surveys, vulnerability detection, Bazel, BMW Group) removed; focus narrowed to recent, directly relevant studies.
- **Conclusion rewritten for significance and impact.** Restructured from summary-style to three significance-focused paragraphs: systemic problem identification, novel findings with implications, and limitations with future directions.
- **Generalizability claims qualified.** Abstract, Discussion, and Conclusion now explicitly acknowledge limitations to open-source platforms. A new "Platform coverage limitations" paragraph names untested systems (Jenkins, GitLab CI, Azure DevOps, CircleCI).
- **All "we"/"our" converted to passive voice.** Only 1 intentional instance remains: "We cannot conclude" (line 533, scientific caveat distinguishing correlation from causation).

Reviewer #1: Ayuba John (8 Comments)

Reviewer #1 provided 8 comments addressing structural organization, presentation style, evaluation methodology, and generalizability claims. All 8 items have been addressed.

Reviewer Comment: *"Revise the abstract so that it is presented as a single, coherent paragraph."*

Response: The abstract was merged from 4 paragraphs into a single coherent paragraph. The "open-source CI/CD platforms" qualifier was also added per R1-C7.

Location in revised manuscript: Abstract section.

Reviewer Comment: *"The introduction section is overly lengthy and should be revised to more clearly highlight the contributions of previous studies in addressing the identified problem. Clearly articulate the existing gap or prevailing challenge, then introduce the proposed methodology with a logical justification explaining why it is suitable for addressing the problem."*

Response: The Introduction was restructured from 5 subsections into 4 focused subsections, following the requested flow: problem → gap → methodology → contributions.

Removed: Tutorial-style CI/CD explanations, protein folding/medical diagnostic analogies, the "Dual-Dataset Validation Strategy" section, and the "Paper Organization" paragraph.

Location in revised manuscript: Lines 1–59 (Introduction).

Reviewer Comment: *"Explicit research questions are not strictly necessary in a research article. You may remove this section and instead clearly state the main contributions of the paper."*

Response: Three changes were made:

Deleted: The entire "Research Questions" subsection (defining RQ1–RQ5).

Renamed: All 5 Results subsection headings changed from "RQn: ..." to descriptive titles.

Removed: All inline RQ references throughout Principal findings, Interpretation, and Strengths/limitations.

Location in revised manuscript: Results section headings and throughout manuscript. Contributions list at lines 36–59.

Reviewer Comment: *"Revise the related work section to be more concise, focusing primarily on recent and closely related studies published in reputable journals."*

Response: All three Related Work subsections were trimmed.

Removed: generic ML survey citations, vulnerability detection surveys, transformer/GNN citations not directly used, Bazel build speed, flaky tests detail, BMW Group, and generic metric surveys.

Retained: ensemble methods, leakage quantification (Grillmeyer et al., ICPE 2025), TravisTorrent, DL-CIBuild, RavenBuild (FSE 2024), Vassallo, Rausch, DORA metrics, MCC, and precision-recall analysis references.

Location in revised manuscript: Lines 60–105 (Related Work Context).

Reviewer Comment: *"The presentation of several sections resembles a tutorial rather than a research article. Consider replacing lengthy textual explanations with figures, flowcharts, and tables where appropriate to improve clarity and conciseness."*

Response: Two new tables replace tutorial-style prose describing the leakage taxonomy:

Table 3 (`tab:leakage_taxonomy`, line 148): Type/Definition/Example Features/Detection Method columns, replacing the 3 subsubsections that described each type in paragraph form.

Table 4 (`tab:clean_vs_leaky`, line 158): Feature disposition by category with counts and examples.

Combined with 6 tables added during R2 revisions and the cost-sensitive analysis table (R1-C6), the manuscript now contains 16 tables (9 new).

Location in revised manuscript: Lines 145–165 (Temporal data leakage taxonomy, Tables 3–4).

Reviewer Comment: *"There is an overreliance on accuracy as the primary performance metric despite the presence of class imbalance, particularly in the GHALogs dataset. Incorporate cost-sensitive analysis, precision-recall curves, and scenario-based error cost evaluations."*

Response: Both ML pipelines (TravisTorrent and GHALogs) were re-run with expanded metrics. Four specific additions address this comment:

1. **MCC added** to evaluation metrics table (Table 1, line 285) and model comparison table (Table 7, line 375) for all 4 classifiers. RF MCC=0.637, GB MCC=0.605, DT MCC=0.564, LR

MCC=0.116, confirming that accuracy alone obscures Logistic Regression's near-chance discrimination.

2. **Precision-recall curves** (Fig 6, line 420) as a dual-panel figure: TravisTorrent (4 models, RF AP=0.938) and GHALogs (clean AP=0.946 vs. leaky AP=0.951).
3. **Cost-sensitive analysis table** (Table 9, `tab:cost_sensitive`, line 430) showing optimal thresholds at FN:FP cost ratios of 1:1, 5:1, 10:1, and 20:1. At 10:1 ratio, threshold 0.31 reduces false negatives by 66% at only 3pp accuracy cost.
4. **Discussion paragraph** (line 562) interpreting MCC and PR tradeoffs for deployment guidance.

Location in revised manuscript: Table 1 (Evaluation metrics section); Table 7 (Leakage-free build prediction performance); Fig 6 and Table 9 (Precision-recall and cost-sensitive analysis); line 562 (Discussion paragraph).

Reviewer Comment: *"Although strong claims of generalizability are made, the evaluation is limited to only two CI/CD platforms. No validation is provided for self-hosted CI systems such as Jenkins, enterprise CI platforms such as GitLab CI and Azure DevOps, or monorepo-heavy environments."*

Response: Three changes were made to qualify generalizability claims:

1. A new "Platform coverage limitations" paragraph (line 625) explicitly names Jenkins, GitLab CI, Azure DevOps, and CircleCI as untested, and acknowledges that self-hosted, enterprise, and monorepo-heavy environments may exhibit different patterns.
2. The Abstract now qualifies accuracy claims with "on open-source CI/CD platforms."
3. The Conclusion (line 680) frames results as "limited to open-source projects on two platforms" rather than claiming universal generalizability.

Location in revised manuscript: Lines 622–628 (Platform coverage limitations); Abstract section (qualifier); lines 667–692 (Conclusions).

Reviewer Comment: *"The conclusion section currently reads more like a summary. Rewrite it to concisely restate the proposed approach and interpret the results, highlighting the significance of the findings, the impact of the study, and potential directions for future research."*

Response: The Conclusion was rewritten from a summary-style section into three significance-focused paragraphs:

Paragraph 1: The taxonomy addresses a systemic methodological problem affecting prediction validity.

Paragraph 2: Key findings, including platform-dependent leakage tax divergence (14.59pp) and project maturity dominance (6.5:1 ratio), with implications for research methodology and CI/CD practice.

Paragraph 3: Limitations alongside concrete future directions (enterprise validation, multi-class prediction, causal inference).

Detailed statistical re-comparisons from Results were removed.

Location in revised manuscript: Lines 667–692 (Conclusions).

Reviewer #2: Anonymous (48 Items, All Completed)

All 48 items from Reviewer #2 have been addressed. The manuscript was reduced from 54 pages to 31 pages, with 9 new tables added, all "we"/"our" instances converted to passive voice, and all 8 Results-section enumerate blocks replaced with concise prose. Below is the complete point-by-point response.

R2-1

Reviewer Comment: *"put all this comparison in table"*

Response: The leakage comparison prose was condensed to reference Table 5 (`tab:leakage_impact`) and Fig 4 (`fig:leakage_impact`).

Interpretation added: Cohen's h=0.62 (large effect) for the 15.07pp drop, contextualized against rigorous prior studies reporting 75–84% accuracy.

Location in revised manuscript: Lines 345–350.

R2-2

Reviewer Comment: *"this paragraph and table both have the same information, remove this paragraph"*

Response: The enumerate block following the ablation table was replaced with a short paragraph referencing Table 6 (`tab:leakage_ablation`).

Key narrative: Type 1 dominates at 79.2% of inflation from just 3 features.

Practitioner guidance: Prioritize Type 1 elimination first.

Location in revised manuscript: Lines 362–368.

R2-3

Reviewer Comment: *"this paragraph contains the same information as in table"*

Response: The enumerate restating model comparison numbers was replaced with statistical analysis referencing Table 7 (`tab:model_comparison`).

Statistics added: Wilcoxon signed-rank tests (RF vs. LR: $p < 0.001$; RF vs. GB: p=0.032), Cohen's d effect sizes, and Bonferroni correction. Numbers now appear only in the table.

Location in revised manuscript: Lines 374–408.

R2-4

Reviewer Comment: *"better to put in table"*

Response: Statistical validation details were converted to Table 15 (`tab:statistical_summary`) with Method/Purpose/Parameters columns, replacing inline methodology prose.

Location in revised manuscript: Lines 494–498 (Table 15).

R2-5

Reviewer Comment: *"table is sufficient"*

Response: The enumerate interpreting the confusion matrix was replaced with a short paragraph referencing Table 8 (`tab:confusion_matrix`).

Key points: Asymmetric error pattern (93.55% recall vs. 66.26% specificity), practical acceptability of false positives, and class-weighted training effectiveness.

Location in revised manuscript: Lines 413–420.

R2-6

Reviewer Comment: *"looks repetition, better to put in analysis table"*

Response: The feature importance enumerate was replaced with a brief discussion and a new Table 10 (`tab:sdlc_distribution`) showing cumulative importance by SDLC phase (Project Context 49.8%, Test Structure 11.3%, Build History 10.1%, Code Metrics 7.7%).

Location in revised manuscript: Lines 439–446 (Table 10).

R2-7

Reviewer Comment: *"put in comparison table"*

Response: The cross-language comparison prose was condensed to reference Table 12 (`tab:cross_language`) and Fig 5 (`fig:cross_language`): Kruskal-Wallis H=2.34, p=0.504, 3.38pp range within random fluctuation.

Location in revised manuscript: Lines 450–454.

R2-8

Reviewer Comment: *"table is enough"*

Response: Three nested Finding blocks with enumerate sub-lists were replaced with concise prose referencing Table 13 (`tab:platform_comparison`): two-proportion z-test (z=1.41, p=0.159, Cohen's h=0.015) and post-hoc power analysis ($>$0.999 power).

Location in revised manuscript: Lines 488–493.

R2-9

Reviewer Comment: *"put the findings in table, whole paper contains lots of repetitive information"*

Response: The 3 Finding blocks were replaced with Table 14 (`tab:key_findings`) containing rows for divergent leakage tax, equivalent clean performance, and build sequence dominance, each with Evidence and Implication columns.

Location in revised manuscript: Lines 488–493 (Table 14).

R2-10

Reviewer Comment: *"table"*

Response: Converted to Table 15 (`tab:statistical_summary`), same table as R2-4, consolidating all statistical methods.

Location in revised manuscript: Lines 494–498 (Table 15).

R2-11

Reviewer Comment: *"put in table"*

Response: The four stakeholder paragraphs were converted to Table 16 (`tab:implications`) with Stakeholder/Key Recommendation columns for CI/CD providers, development teams, researchers, and tool builders.

Location in revised manuscript: Line 602 (Table 16).

R2-12

Reviewer Comment: *"already provided the comparison, then why the full comparison in conclusion?"*

Response: All detailed statistical re-comparisons were removed from the Conclusion. Key numbers appear once only in Results tables, with the Conclusion focusing on significance and implications (see R1-C8).

Location in revised manuscript: Lines 667–692 (Conclusions).

R2-13

Reviewer Comment: *"each contribution consist on one or two lines only, no need to write full length paragraph for each contribution, its research paper not thesis"*

Response: Each contribution was compressed to 1–2 sentences. For example, Contribution 1 now reads (line 38): "A systematic taxonomy categorizing leaky features as Direct Outcome Encoding, Execution-Dependent Metrics, or Future Information Leakage. Cross-platform validation reveals divergent leakage impact: 15.07pp inflation on TravisTorrent versus 0.48pp on GHALogs."

Location in revised manuscript: Lines 36–59 (Research Contributions).

R2-14

Reviewer Comment: *"be concise, and dnt use,,we"*

Response: The TravisTorrent dataset description was substantially condensed.

Removed: Feature category enumeration and "we analyzed"/"Our sample" phrasing.

Compressed: Power analysis reduced to a single sentence.

Location in revised manuscript: Lines 118–130 (TravisTorrent dataset).

R2-15

Reviewer Comment: *"be concise"*

Response: The GHALogs dataset description was condensed from 4 paragraphs to 2 paragraphs. Per-feature enumerations were removed and API/sampling details were compressed.

Location in revised manuscript: Lines 131–144 (GHALogs dataset).

R2-16

Reviewer Comment: *"be concise"*

Response: Each finding in the Principal Findings subsection was reduced from a full paragraph with re-quoted numbers to 1–2 sentences with table references only.

Location in revised manuscript: Lines 507–526 (Principal findings).

R2-17

Reviewer Comment: *"already mentioned information"*

Response: The Interpretation of findings and Comparison with prior work subsections were condensed. Restated data values and detailed numbers duplicating Results content were removed.

Location in revised manuscript: Lines 527–601 (Interpretation and Comparison).

R2-18

Reviewer Comment: *"be specific and concise"*

Response: The Future Research paragraphs were converted to a bulleted list with 1–2 sentences each, covering fine-grained failure classification, causal inference, transfer learning, multi-objective scheduling, and explainable AI.

Location in revised manuscript: Lines 648–666 (Future research directions).

R2-19

Reviewer Comment: *"is this a story or a research paper, be concise"*

Response: Addressed together with R2-18. The narrative style was trimmed to concise bullet points.

Location in revised manuscript: Lines 648–666 (Future research directions).

Table Caption Placement (R2-20 through R2-22):

R2-20

Reviewer Comment: *"here add table caption"*

Response: All table captions were shortened to a bold title followed by a brief key finding note.

Location in revised manuscript: Tables 1–16 throughout manuscript.

R2-21

Reviewer Comment: *"caption must be with table, not one paragraph before"*

Response: Explanatory paragraphs that previously separated captions from their tables were removed. All captions now immediately precede their tables.

Location in revised manuscript: All table environments throughout the manuscript.

R2-22

Reviewer Comment: *"same mistake, check for all tables, caption must be with the table"*

Response: Checked and corrected across all 16 tables.

Location in revised manuscript: All table environments throughout the manuscript.

Paragraph / Line Breaks (R2-23 through R2-28):

R2-23

Reviewer Comment: *"paragraph must starts with new paragraph"*

Response: Corrected paragraph breaks in the Data preprocessing section.

Location in revised manuscript: Lines 192–230 (Data preprocessing).

R2-24

Reviewer Comment: *"start with new line"*

Response: The ML Pipeline subsection now starts on a new line.

Location in revised manuscript: Line 231 (Machine learning models).

R2-25

Reviewer Comment: *"start with new line"*

Response: The Cross-validation strategy subsection now starts on a new line.

Location in revised manuscript: Line 290 (Cross-validation strategy).

R2-26

Reviewer Comment: *"start with new line"*

Response: The Datasets subsection now starts on a new line.

Location in revised manuscript: Line 115 (Datasets).

R2-27

Reviewer Comment: *"start with next paragraph"*

Response: The Results introduction now has proper paragraph separation.

Location in revised manuscript: Lines 322–326 (Results introduction).

R2-28

Reviewer Comment: *"start with next line"*

Response: The Feature importance section now has proper paragraph separation.

Location in revised manuscript: Line 439 (Feature importance analysis).

Formulas (R2-29 and R2-30):

R2-29

Reviewer Comment: *"its research paper, not thesis, if necessary, put all formulas in a table"*

Response: The 4 inline equation environments for evaluation metrics (Accuracy, Precision, Recall, F1-Score) were replaced with Table 1 (`tab:evaluation_metrics`).

Table format: 7 rows with Metric/Formula/Interpretation columns, including MCC and Average Precision added for R1-C6.

Location in revised manuscript: Lines 283–287 (Table 1).

R2-30

Reviewer Comment: *"better to putt all information in table"*

Response: The statistical analysis description was condensed into Table 2 (`tab:statistical_methods`) with 7 rows covering all statistical methods.

Location in revised manuscript: Lines 288–289 (Table 2).

R2-31

Reviewer Comment: *"be concise, and dnt use„we"*

Response: All "we"/"We" and "our"/"Our" instances were converted to passive voice throughout the manuscript.

Exception: One intentional instance remains at line 533: "We cannot conclude that artificially aging a project. . . would improve build success rates", a scientific caveat distinguishing correlation from causation.

Location in revised manuscript: Global change. Remaining instance at line 533.

R2-32

Reviewer Comment: *"(highlighted "We applied")"*

Response: Changed to "StandardScaler normalization was applied" (line 208).

Location in revised manuscript: Line 208 (Data preprocessing).

R2-33

Reviewer Comment: *"use of we ....?"*

Response: All "we" instances in the Methods section converted to passive voice.

Location in revised manuscript: Lines 106–321 (Materials and methods).

R2-34

Reviewer Comment: *"(highlighted "We" in evaluation metrics)"*

Response: Converted to passive voice in the Evaluation Metrics section: "Standard classification metrics were employed" (line 283).

Location in revised manuscript: Lines 283–289 (Evaluation metrics).

R2-35 through R2-39 (Contributions list)

Reviewer Comment: *"Highlighted: Contributions 1–5 (full paragraphs for each contribution)."*

Response: All 5 contributions compressed to 1–2 sentences each, consistent with R2-13.

Location in revised manuscript: Lines 36–59 (Research Contributions).

R2-40 (Leakage impact results)

Reviewer Comment: *"Highlighted: Severe performance inflation numbered list."*

Response: Enumerate block replaced with a short paragraph referencing Table 5 (`tab:leakage_impact`).

Location in revised manuscript: Lines 345–350.

R2-41 (Model comparison results)

Reviewer Comment: *"Highlighted: Random Forest performance numbered list."*

Response: The enumerate was replaced with a short paragraph referencing Table 7 (`tab:model_comparison`) with added statistical analysis (Wilcoxon tests, effect sizes).

Location in revised manuscript: Lines 374–408.

R2-42 (Feature importance)

Reviewer Comment: *"Highlighted: Feature importance numbered findings."*

Response: The enumerate was replaced with a short paragraph plus new Table 10 (`tab:sdlc_distribution`).

Location in revised manuscript: Lines 439–446.

R2-43 (Table and figure descriptions)

Reviewer Comment: *"Highlighted: Verbose descriptions accompanying tables and figures."*

Response: Table and figure captions shortened to a bold title followed by a key finding note.

Location in revised manuscript: All table and figure captions throughout manuscript.

R2-44 (Cross-language and cross-platform captions)

Reviewer Comment: *"Highlighted: Table descriptions and figure captions for cross-language and cross-platform results."*

Response: Captions for Tables 12–13 and Figs 5–6 shortened to match the same format.

Location in revised manuscript: Tables 12–13 and Figures 5–6 (float elements).

R2-45 (Discussion: Infrastructure hypothesis)

Reviewer Comment: *"Highlighted: Infrastructure investment hypothesis."*

Response: Condensed by removing parenthetical explanations. Key finding retained: 11.3pp difference ($p < 0.001$, Cohen's h=0.24).

Location in revised manuscript: Lines 529–531 (Infrastructure investment hypothesis).

R2-46 (Discussion: Comparison with prior work)

Reviewer Comment: *"Highlighted: Comparison with prior work."*

Response: Condensed from 3+ paragraphs to a single paragraph covering three dimensions: scale and diversity, methodological rigor, and deployment viability.

Location in revised manuscript: Lines 583–601 (Comparison with prior work).

R2-47 (Future work and Conclusion)

Reviewer Comment: *"Highlighted: Future work and conclusion sections."*

Response: Future work converted from full paragraphs to a concise bulleted list. Conclusion restructured per R1-C8.

Location in revised manuscript: Lines 648–692 (Future research and Conclusions).

R2-48 (Conclusion statistics)

Reviewer Comment: *"Highlighted: Detailed statistical comparisons in conclusion."*

Response: All detailed statistical re-comparisons removed. Key numbers appear once only in Results tables; Conclusion provides interpretation only.

Location in revised manuscript: Lines 667–692 (Conclusions).

The accompanying tracked-changes PDF highlights all modifications for convenient review.

Sincerely,
Lalit Narayan Mishra (on behalf of all authors)
