# Supplementary material for: A taxonomy for detecting and preventing temporal data leakage in machine learning-based build prediction: A dual-platform empirical validation
Source: PLoS One. 2026 May 27;21(5):e0340167. doi: 10.1371/journal.pone.0340167 (PMC13215522; doi:10.1371/journal.pone.0340167)
Supplement: Attachment — Submitted filename: Reviewers Comments.pdf [file pone.0340167.s004.pdf]

Title: A taxonomy for detecting and preventing temporal data leakage in machine learning based build prediction: A dual-platform empirical validation

Manuscript ID: PONE-D-25-66966R1

Article type: Research article

PLOS ONE

Reviewer's Comments:

After reviewing the authors' responses and the revised manuscript, I am impressed with the meticulous way they addressed all the reviewers' comments to a commendable extent. I have no doubt that the manuscript has been significantly improved and is now suitable for publication in this esteemed journal for the benefit of its readers.
